# Geometric properties of caudate and putamen mark the progression of Huntington’s disease

**DOI:** 10.1162/IMAG.a.1351

**Published:** 2026-08-24

**Authors:** Dorian Pustina, Haikel Bogale, Sandhitsu Das, Brian Avants, Swati Sathe, Cristina Sampaio, Andrew Wood

**Affiliations:** CHDI Management, Inc., the company that manages the scientific activities of CHDI Foundation, Inc., Princeton, NJ, United States; Rancho BioSciences, Rancho Santa Fe, CA, United States; Paid consultant, Rancho BioSciences, Rancho Santa Fe, CA, United States; Department of Radiology and Medical Imaging, University of Virginia, Charlottesville, VA, United States

**Keywords:** morphometric, HD-ISS, monitoring, biomarker, surrogate, endpoint, prognostic, structural MRI

## Abstract

Volumetric MRI of the caudate and putamen is a robust biomarker of striatal degeneration in Huntington’s disease (HD). Striatal degeneration may be reflected beyond volume alone, in additional shape and intensity alterations. We evaluated five novel morphometric biomarkers of the caudate and putamen—curvature, flatness, eccentricity, elongation, and focal assessment of divergence estimation (FADE; T1-weighted signal shift)—using cross-sectional and longitudinal MRI data from healthy controls (N = 100), individuals with premanifest HD (preHD; N = 100), and individuals with early manifest HD (earlyHD; N = 100). Geometric measures showed significant cross-sectional group differences with large effect sizes, although volume remained the most sensitive individual biomarker. Longitudinally, most geometric measures detected abnormal rates of change, particularly in the caudate, with medium-to-large effect sizes. The direct comparison between preHD and earlyHD proved challenging for all biomarkers; however, elongation yielded the largest effect size and outperformed volume in this comparison. FADE detected abnormal signal increases in HD groups at baseline but not longitudinally. Power analyses confirmed that volume retains the highest sensitivity, while geometric measures follow closely. Combining volume and geometric measures improved the cross-validated classification accuracy starting at low sample sizes. Correlations between scores and results from atrophy simulations confirmed that HD leaves two signatures on the striatum: loss of volume and loss of shape. While volume remains the strongest standalone biomarker, geometric measures provide complementary information that sharpens disease detection and may enhance biomarker frameworks in future HD clinical trials.

## 1 Introduction

Huntington’s disease (HD) is an autosomal dominant neurodegenerative disorder caused by an expanded CAG repeat in exon 1 of the huntingtin (*HTT*) gene. The resulting mutant huntingtin (HTT) protein triggers neuropathologic cascades that preferentially affect the medium spiny neurons of the caudate and putamen, leading to atrophy. Striatal atrophy is the most prominent and consistent neuropathological hallmark of HD. Post-mortem studies (Hedreen et al., 2024; Vonsattel et al., 1985) and numerous MRI investigations have repeatedly confirmed caudate and putamen volume loss very early in disease progression, including decades before clinical motor diagnosis (Aylward, 2014; Kinnunen et al., 2021; Scahill et al., 2020). In the recently developed Huntington’s Disease Integrated Staging System (HD-ISS), caudate and putamen volume are exclusive markers of Stage 1 disease progression, and the only biological markers in the entire range of progression (Tabrizi et al., 2022). Following the trail of decade-long evidence, clinical trial sponsors have included striatal volume as endpoint in clinical trials, expecting the candidate treatment to slow the rate of volume loss (e.g., GENERATION-HD, LEGATO-HD, PIVOT-HD, PRECREST, SELECT-HD, SIGNAL-HD, REV-HD, TRIHEP3).

Biological markers of early HD progression have become increasingly important in recent years. Evidence from clinical trials show that disease-modifying treatments may be more effective in earlier, rather than advanced, stages of the disease (see press releases regarding GENERATION-HD and PIVOT-HD trials). These results are not surprising. In principle, it is easier to slow disease progression before irreparable damage occurs rather than reverse neuronal damage or grow new neurons. By the time of clinical motor diagnosis, striatal atrophy is already profound. Estimates from MRI studies show that caudate has lost ~52–70% of the normal volume, while putamen has lost ~43–67% (Aylward et al., 2004; Kinnunen et al., 2021; Vonsattel et al., 1985). At the same time, cognitive and clinical scores remain largely unaffected until clinical motor diagnosis, leaving imaging biomarkers as the best option to monitor early HD progression and assess treatment efficacy.

As mentioned above, volume loss is the most established biomarker of structural degeneration in HD. However, structural degeneration is reflected in other measures besides volume. For example, Vonsattel et al. (1985) noticed that HD progression is marked by a change in the curvature of the lateral ventricle wall, which might be related to the degeneration of the adjacent caudate nucleus. Observing post-mortem brains, Vonsattel et al. described how lateral ventricle wall looks convex in healthy brains, flat in early stages of HD progression, and then concave in later stages. Radiologists have used other geometric measures, such as the bicaudate index (Dhok et al., 2020) or the ratio of distances between bilateral ventricle horns and bilateral caudates (Ho et al., 1995). Researchers have also analyzed the inward or outward deformation of the boundaries of caudate and putamen (i.e., surface displacement) and reported more dorso-medial degeneration rather than ventro-lateral, although the pattern of surface displacement has not been consistent between studies (Kim et al., 2017; Tang et al., 2019; van den Bogaard et al., 2011; Wilkes et al., 2023; Younes et al., 2014).

Most of the findings from surface and geometric measures are not developed as concrete biomarkers for potential use in clinical trials. In part, this is due to technical challenges. For example, surface measures create a virtual mesh of thousands of vertices that must be reduced or combined to single endpoints. Dimensionality reduction poses several questions: Should only one part of the structure be used as biomarker? Which part should be selected if studies are inconsistent? Should vertices be averaged together? etc. The findings reported in the literature are also impacted by limitations. Sometimes the study does not investigate longitudinal changes (van den Bogaard et al., 2011), other times the study has small sample sizes (Kim et al., 2017; Younes et al., 2014), and in other cases the study relies on manual segmentations that are not feasible for clinical trials (Wilkes et al., 2023). The most important limitation, however, is the lack of direct comparison of new candidate biomarkers with existing robust biomarkers, such as volume. One study compared the effect sizes of surface displacement vs. volume and found that surface displacement can outperform volume measurements, providing preliminary evidence of the value of other, non-volumetric markers of degeneration (Tang et al., 2019).

Our research group has processed and reviewed MRI neuroimaging data from nearly 7,000 visits in 8 HD natural history studies (Pustina et al., 2024). During visual inspections of the segmentations, we noticed patterns of morphologic anomalies that seem to be related to HD progression. For example, we noticed that caudate not only becomes smaller in HD individuals but also looks thinner and flatter. Occasionally, it looks like a thin strip of gray matter barely recognizable next to the ventricle wall. Putamen also seems to become thinner, although this is less noticeable to the naked eye. We also noticed that caudate and putamen look somewhat “faded” in T1-weighted scans of HD individuals. That is, the structure looks brighter than other gray matter structures and more similar to white matter. This “fading” effect may be related to the structure’s internal degradation that is not captured by volume or geometric measures.

In this study, we aimed to quantify and rigorously investigate the biomarking value of five new morphometric measures of the caudate and putamen: (1) curvature, (2) flatness, (3) eccentricity, (4) elongation, and (5) focal assessment of divergence estimation (FADE, T1-intensity shift). To evaluate their biomarking sensitivity, we compared group differences between healthy controls (HC), premanifest HD (preHD), and early manifest HD (earlyHD) participants. We computed cross-sectional and longitudinal effect sizes and benchmarked each new candidate biomarker against volume as the established biomarker of reference. We also generated statistical power curves and cross-validated discrimination performance at various sample sizes. We hypothesized that the new geometric and intensity scores would show significant differences when comparing groups, reflecting our informal observations, but, given the novel nature of the scores and the imperfect FreeSurfer segmentations, we had no specific hypothesis on how the effect sizes would compare with volume.

## 2 Methods

### 2.1 Population

We analyzed data from 300 participants from the Track-HD and TrackOn-HD studies: N = 100 HC, N = 100 preHD, and N = 100 earlyHD (Kloppel et al., 2015; Tabrizi et al., 2009). The studies were approved by the local ethics committees, and written informed consent was obtained from each participant. Data from participants who enrolled in both studies were concatenated to form longer longitudinal follow-ups and then formatted in the Brain Imaging Data Structure as part of data harmonization (Pustina et al., 2024). Data harmonization was applied only to the file and folder structure, not the voxel intensities or derived biomarker values. We maintained the original grouping criteria applied during study conduct (see “Track-HD Study Protocol,” 2010) and avoided the use of new HD-ISS grouping criteria because HD-ISS uses caudate and putamen volume for assignment to Stage 1, which would make subsequent group comparisons circular. The 100 participants of each group were randomly selected from the available pool of Track participants. Table 1 shows demographic information for each group. Groups were balanced for sex, education, CAG repeat length, and study site. The preHD group was significantly younger than HC and earlyHD, while the latter two groups had similar ages. The earlyHD group did not participate in the subsequent TrackOn-HD study, and, for this reason, it had less visits in average than the other two groups.

**Table 1.** Demographic characteristics of healthy controls (HC), premanifest HD (preHD), and early manifest HD (earlyHD).

	HC N = 100	preHD N = 100	earlyHD N = 100
Age	45.92 (9.83)	39.63 (8.22)* ^†^	48.28 (9.99)^†^
Education	4.05 (1.32)	3.91 (1.23)	3.70 (1.33)
Sex (% female)	59.0%	57.0%	55.0%
Visits/participant	4.83 (2.01)	5.09 (1.92)	3.38 (0.96)*
CAG	NA	43.72 (2.17)	44.03 (3.51)
Study site 1/2/3/4	25/30/17/28	25/22/21/32	27/22/24/27

Education was labeled on a 7-point scale using “International Standard Classification of Education, ISCED 1997” (2003).

* Significant difference of the group showing the asterisk compared with HC (p < 0.01). ^†^Significant difference between preHD and earlyHD (p < 0.01)

### 2.2 MRI data collection

MRI data were collected at four sites using similar contrast and scan parameters. Details on the specific data collection parameters are described in the supplementary file of Tabrizi et al. (2009). Briefly, T1-weighted image volumes were acquired using a 3D MPRAGE acquisition sequence on 3.0 T Siemens and Phillips scanners with the following imaging parameters: TR = 2200 ms (Siemens)/7.7 ms (Philips), TE = 2.2 ms (S)/3.5 ms (P), FA = 10(S)/8(P), FOV = 28 cm (S)/24cm (P), matrix size 256 x 256(S)/224 x 224(P), 208(S)/164(P) with sagittal slices to cover the entire brain, a slice thickness of 1.0 mm and no gap. MRI scans underwent manual quality assessment during study conduct, and visits without an acceptable MPRAGE scan were not included in this study.

### 2.3 MRI volumetric segmentation

The FreeSurfer v6 longitudinal pipeline was used for whole brain parcellation, as described in Pustina et al. (2024). The FreeSurfer’s volumetric scores have been used in several other research projects, including the development of the HD-ISS (Tabrizi et al., 2022). The first author (D.P.) inspected FreeSurfer segmentations using a 5-point rating scale, as described in Pustina et al. (2024). Only visits that had an acceptable segmentation (QC = 3-5) were included in this study, totaling 1330 visits for the 300 participants. In the current study, we used the segmentations of caudate, putamen, cortical ribbon, and white matter from the FreeSurfer’s whole brain parcellation file (*aparc.DKTatlas+aseg.mgz*).

### 2.4 Regions of interest (ROIs)

We investigated the caudate and putamen separately for the left and right hemispheres. In addition to caudate and putamen, we also ran exploratory analyses of the caudate–ventricle wall to investigate the value of this region as suggested by the seminal post-mortem study of Vonsattel et al. (1985). The caudate–ventricle wall was computed by dilating the lateral ventricle segmentation by one voxel and selecting the overlap with the caudate segmentation (see example in “Supplementary Video—Caudate Ventricle Wall.mp4”). Note that Vonsattel et al. described the ventricle wall convexity on 2D coronal slices, while our geometric descriptors use the entire 3D shape of the caudate–ventricle wall.

### 2.5 Morphometric measures

We computed six morphometric scores for each region: volume, curvature, flatness, elongation, eccentricity, and FADE. Volume was obtained from the tables produced by FreeSurfer as tabulated in the Volume_mm3 column of aseg.stats file. To measure geometric properties, we developed a new tool named Curvanato (https://github.com/stnava/curvanato, v0.1.0) (Avants & Gee, 2003; Ibáñez et al., 2019). Curvanato relies on other software for image manipulation and other computations, among which are ANTs v0.5.4 and python v3.10. Before computing the geometric measures, Curvanato resamples the binary mask at 0.5 mm isotropic voxels and applies a modest amount of smoothing with kernel size of the order of one voxel. The following measures were computed with Curvanato:

- **Curvature** (kappa) is quantified as a local scalar measure of surface bending derived from the regional boundary, with higher values indicating stronger convex or concave deformation and lower values approaching planarity. For each structure, we exported curvature maps and computed a mean kappa value as a summary measure.
- **Flatness** characterizes how closely a region approximates a planar form. It is defined as the ratio of the smallest to the largest principal axis eigenvalue, with values closer to 1 reflecting more planar geometry and lower values reflecting more compact shapes.
- **Elongation** measures the degree to which a structure is stretched along its major axis. It is calculated as the ratio of the longest to the intermediate principal axis, with higher values denoting more rod-like forms and values near 1 indicating more isotropic geometry.
- **Eccentricity** describes deviation from spherical symmetry based on the relative lengths of the principal axes of a fitted ellipsoid. Values near 0 correspond to near sphericity, whereas values approaching 1 indicate more elongated or flattened structures.

The Curvanato tool also computes values of surface area and thickness, which were not of interest for this study.

#### 2.5.1 FADE (focal assessment of divergence estimation)

**Fig. 1.**
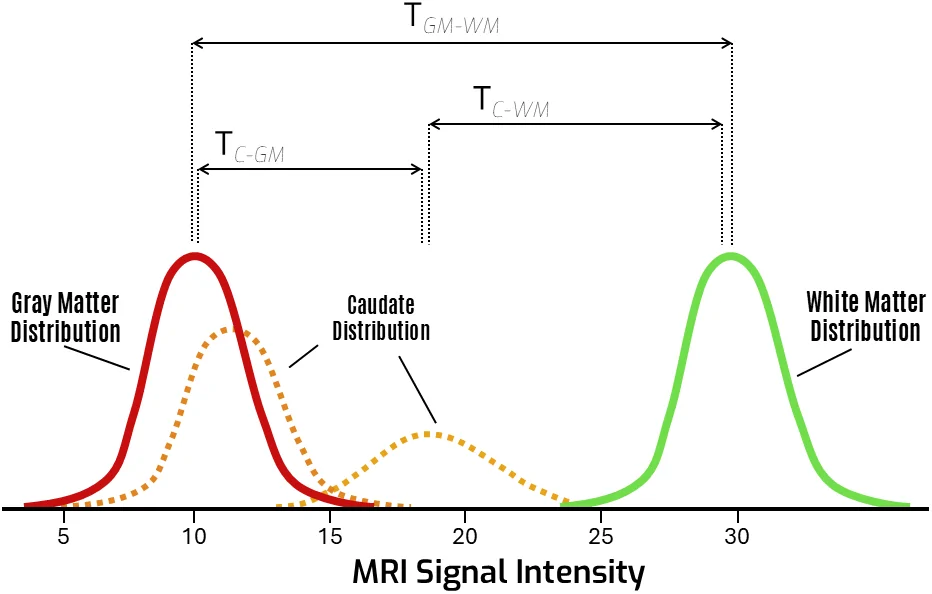
Conceptual depiction of voxel intensity distribution of GM (red), WM (green), and two caudate examples (orange).

The FADE score was calculated outside the Curvanato tool. FADE captures the divergence of voxel intensities in caudate or putamen away from typical gray matter (GM) and toward white matter (WM) values. Figure 1 shows the rationale behind the FADE formula using hypothetical voxel intensity distributions of GM, WM, and caudate.

$$\begin{array}{c} FADE=T_{C-GM}\times \;\frac{T_{GM-WM}}{T_{C-WM}}. \end{array}$$ (1)

Distances between distributions were measured with T-scores obtained from the stats.ttest_ind function of the stats package within the scipy python library (equal_var=False option). In Figure 1, the distance denoted by T_C-GM_ captures the shift of striatal voxel intensity away from typical gray matter (GM) intensity, T_C-WM_ captures the distance between the caudate and white matter (WM) distributions, and T_GM-WM_ captures the distance between GM and WM distributions. Thus, the T_GM-WM_/T_C-WM_ ratio serves as a weighting factor to capture the “fading effect”. If the caudate distribution is similar to the GM distribution, the T_GM-WM_/T_C-WM_ ratio would be around 1, whereas if the caudate distribution gets closer to the WM distribution, the numerator will be larger and the T_GM-WM_/T_C-WM_ ratio will be > 1, amplifying the fading effect.

Voxel intensities were obtained from the *norm.mgz* file generated by Freesufer, which contains the MPRAGE scan of the participant after applying only bias correction and intensity scaling. We aimed to keep image manipulation to a minimum and avoided more complex processing. The masks used to compute regional voxel intensities were taken from the *aparc.DKTatlas+aseg.mgz* file. GM intensity values were obtained from the entire cortical ribbon without the somatosensory and occipital areas, which are known to be affected in HD (Johnson et al., 2015; Rosas et al., 2008; Stoebner et al., 2023). We removed the top and bottom 10^th^ percentile of GM values to avoid outlier voxels or mis-segmentations. WM intensity values were obtained from WM segmentation mask after eroding the mask by one voxel and removing the top and bottom 5^th^ percentiles to avoid outlier values. For caudate and putamen, we conducted a search for the optimal way to remove mis-segmentations. We were initially concerned of dark peri-vascular spaces in putamen, which do not constitute neural tissue (Chan et al., 2021). We noticed that FreeSurfer correctly removed large peri-vascular spaces from the putamen segmentation. Further removal of small peri-vascular spaces requires complex processing that did not align with our aim to keep the FADE score easy to compute. Thus, we simply removed the bottom 10^th^ percentile of values for putamen and caudate voxel intensity distributions to remove eventual perivascular spots and keep a more representative set of gray matter voxels.

Example code to derive the scores described above is given in “Supplementary File—Example Code”.

### 2.6 Statistical analyses

First, we tested whether geometric measures are related to intracranial volume using Pearson correlations on all 300 participants using only baseline visit data. ICV correction was deemed necessary (see Section 3) and was done by fitting a linear regression on all available data, including longitudinal timepoints, and obtaining the residual scores (i.e., residualization method of ICV correction, see J. Wang et al. (2024)).

Since the preHD group was younger than the other two groups, we removed the effect of age by fitting a linear regression model on control participants and removing the effect from all participants (i.e., residualizing). This process was done separately for all morphometric measures in this study.

The main analyses focused on running pairwise group comparisons between HC, preHD, and earlyHD and computing the effect size. We tested first the cross-sectional (baseline) comparisons to characterize the anomalies accumulated in HD individuals until the day of the first scan. After this, we compared groups for the rate of longitudinal change. The rate of longitudinal change was computed by calculating the percent change between baseline and each follow-up timepoint, calculating the annualized value, and then averaging these scores. For example, if a participant had a baseline and six follow-up timepoints, we would compute the six annualized longitudinal changes and average them together.

Preliminary tests showed that several measures did not have a normal distribution or equal variance between groups (see “Supplementary File—Normality and Variance Tests”). For this reason, we computed the non-parametric rank-biserial effect size rather than the parametric Cohen’s D measure. The rank-biserial effect sizes range from 0 to 1. Effect sizes >0.11 are considered small, >0.28 are considered medium, and 0.43 are considered large (Vargha & Delaney, 2000). We used non-parametric Wilcoxon rank-sum tests to compute p-values and label the significant group differences (p < 0.05, corrected with the false discovery rate method, FDR, (Benjamini & Hochberg, 1995)). For compatibility with existing literature, we provide also tables with Cohen’s D effect sizes in “Supplementary File—Cohens D Effect Sizes”.

Besides the effect sizes, we computed also statistical power curves. Power (the probability of correctly rejecting the null hypothesis with alpha = 0.05) was calculated at increasing sample sizes from N = 3 to N = 150 with 1,000 repetitions for each sample size using bootstrapped sampling (with replacement). The results were plotted as power curves (y-axis) that vary by sample size (x-axis) and include the standard deviation of the 1,000 repetitions at each sample size.

We used random forest models to check whether combining geometric measures with volume improves the classification accuracy compared with using volume alone. Group classification was obtained for HC vs. preHD or HC vs. earlyHD using random forest models with 500 trees, and cross-validated accuracy scores were obtained from out-of-bag data. Similar to the power curves, these random forests were repeated 1,000 times for each sample size (N = 5-150) using bootstrapping with replacement, and discriminatory curves were plotted. The out-of-bag accuracy is a reliable cross-validated estimate similar to k-fold or cross-study validation both in our experience and in the literature (see Breiman, 2001).

To provide a comprehensive report of the new morphometric scores, Supplementary Files include descriptive statistics (“Supplementary File—Descriptive Statistics”), correlations between morphometric scores (“Supplementary File—Correlation Matrices”), and variance partition into group, age, site, and sex factors (“Supplementary File—Variance Partition”).

All statistical analyses were conducted in R v4.3.1 [R Core Team (2025)].

## 3 Results

### 3.1 Correction for intracranial volume

All measures showed significant correlation with ICV, except curvature in the right caudate that showed a trend (p = 0.074, Table 2). Volume had generally a stronger relationship with ICV than geometric measures. Detailed scatterplots of the relationship of each measure with ICV are given in “Supplementary File—ICV_correlations”. Given the widespread relationship with ICV on most measures, we corrected all measures, except FADE, for ICV and used residualized, age corrected, scores for the remaining analyses.

**Table 2.** Pearson correlations and corresponding p-values of each morphometric measure with ICV.

	Correlations with intracranial volume			
	Caudate		Putamen	
	Left	Right	Left	Right
Volume	r = 0.281 p < 0.001*	r = 0.299 p < 0.001*	r = 0.285 p < 0.001*	r = 0.300 p < 0.001*
Curvature	r = -0.118 p = 0.041*	r = -0.103 p = 0.074	r = -0.131 p = 0.023*	r = -0.156 p = 0.007*
Flatness	r = 0.121 p = 0.036*	r = 0.138 p = 0.017*	r = 0.207 p < 0.001*	r = 0.181 p = 0.002*
Elongation	r = 0.132 p = 0.023*	r = 0.134 p = 0.020*	r = 0.187 p = 0.001*	r = 0.155 p = 0.007*
Eccentricity	r = 0.122 p = 0.035*	r = 0.139 p = 0.016*	r = 0.208 p < 0.001*	r = 0.182 p = 0.002*

FADE was not tested or corrected for ICV because MRI signal intensity is not expected to depend on head size.

Asterisks mark significant correlations with p < 0.05.

### 3.2 Group comparisons at baseline (cross-sectional)

**Fig. 2.**
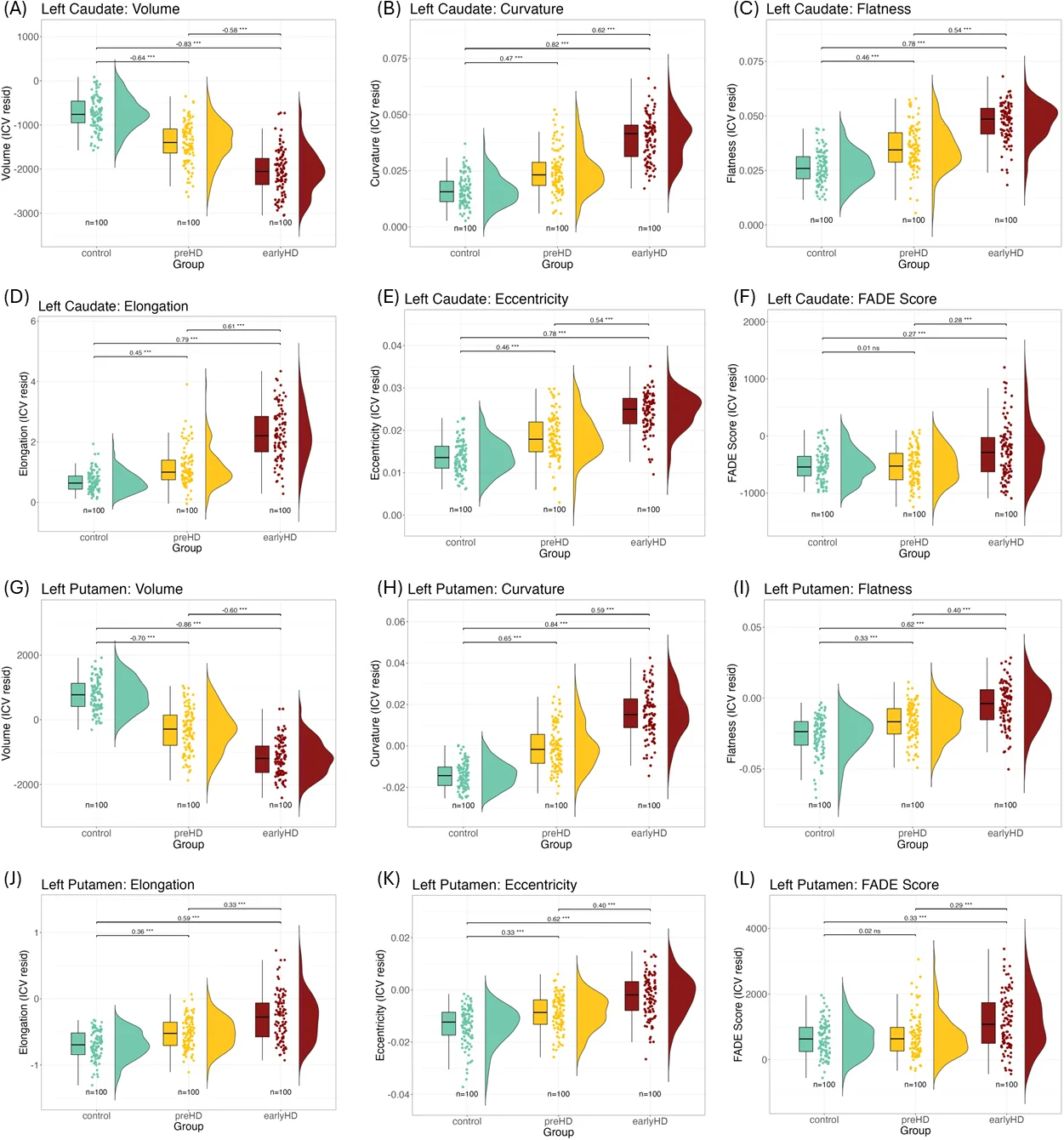
Violin plots with overlaid boxplots illustrate group differences in six morphometric measures of the striatum: volume, curvature, flatness, elongation, eccentricity, and FADE score. Measures are adjusted for age and intracranial volume (ICV). Panels (A–F) show left caudate, panels (G–L) show left putamen. Groups include healthy controls (green), preHD, (yellow), and earlyHD (red). Boxplots indicate the median and interquartile range, while the violin shapes represent kernel density distributions of individual participant values (n = 100 per group). Horizontal bars above the plots show the effect size for each pairwise comparison, marked by asterisks for Wilcoxon tests with *p < 0.05, **p < 0.01, and ***p < 0.001 (uncorrected, see FDR corrected in Table 3).

Figure 2 shows representative violin plots of cross-sectional measures in left caudate and left putamen. The violin plots for the right hemispheric structures are given in “Supplementary File—RH Violin Plots”. Figure 2 illustrates the decrease of volume from healthy controls to preHD and earlyHD groups, while geometric scores increase (curvature, flatness, elongation, and eccentricity). The FADE score remains similar in HC and preHD but is increased in earlyHD.

Table 3 shows effect sizes for all structures, all imaging features, and all group comparisons, both for cross-sectional and longitudinal comparisons. Cross-sectional comparisons were dominated by large and moderate effect sizes for volume and geometric measures, while FADE showed small or insignificant effect sizes. Volume and curvature showed similar effect sizes overall, and differences were often marginal. The other three geometric measures (flatness, elongation, eccentricity) had a mix of large and moderate effect sizes. Importantly, all group comparisons were significant with all volume and geometric measures (p < 0.05, FDR corrected).

**Table 3.** Aggregate overview of absolute rank biserial effect sizes for each pairwise group comparison, each morphometric measure, and each brain region.

		Cross-sectional rank-biserial				Longitudinal rank-biserial			
		Caudate		Putamen		Caudate		Putamen	
		Left	Right	Left	Right	Left	Right	Left	Right
HC vs. earlyHD	Volume	**0.83**	**0.83**	**0.86**	**0.86**	**0.72**	**0.72**	**0.52**	**0.57**
	Curvature	**0.82**	**0.81**	**0.84**	**0.85**	**0.32**	**0.18**	**0.32**	**0.29**
	Flatness	**0.78**	**0.79**	**0.62**	**0.68**	**0.39**	**0.55**	0.07	**0.29**
	Elongation	**0.79**	**0.80**	**0.59**	**0.66**	**0.53**	**0.65**	0.09	**0.31**
	Eccentricity	**0.78**	**0.79**	**0.62**	**0.68**	**0.38**	**0.56**	0.06	**0.29**
	FADE	**0.27**	**0.26**	**0.33**	**0.22**	0.04	0.06	0.04	0.02
HC vs. preHD	Volume	**0.64**	**0.66**	**0.70**	**0.69**	**0.78**	**0.74**	**0.61**	**0.65**
	Curvature	**0.47**	**0.45**	**0.65**	**0.66**	**0.40**	**0.43**	**0.45**	**0.35**
	Flatness	**0.46**	**0.49**	**0.33**	**0.36**	**0.51**	**0.49**	**0.18**	**0.16**
	Elongation	**0.45**	**0.45**	**0.36**	**0.41**	**0.59**	**0.57**	0.13	**0.22**
	Eccentricity	**0.46**	**0.49**	**0.33**	**0.36**	**0.51**	**0.49**	**0.18**	**0.16**
	FADE	0.01	0.03	0.02	0.02	0.03	0.09	0.10	0.05
preHD vs. earlyHD	Volume	**0.58**	**0.57**	**0.60**	**0.61**	**0.23**	**0.19**	0.03	0.02
	Curvature	**0.62**	**0.62**	**0.59**	**0.63**	0.02	0.12	0.11	0.01
	Flatness	**0.54**	**0.53**	**0.40**	**0.49**	0.08	0.13	**0.24**	0.16
	Elongation	**0.61**	**0.61**	**0.33**	**0.43**	0.09	**0.31**	**0.21**	0.16
	Eccentricity	**0.54**	**0.53**	**0.40**	**0.49**	0.08	0.13	**0.24**	0.17
	FADE	**0.28**	**0.25**	**0.29**	**0.22**	0.08	0.12	0.04	0.04

Cross-sectional effect sizes are shown in the left columns while longitudinal effect sizes are shown in the right columns. Bold fonts indicate comparisons with p < 0.05 (FDR corrected).

For HC vs. earlyHD, the average effect sizes for volume were r = 0.85 ± 0.02 (mean ± standard deviation) while the combined geometric measures had effect size of r = 0.74 ± 0.09. For HC vs. preHD, volume’s effect sizes were r = 0.67 ± 0.03, while geometric effect sizes were r = 0.45 ± 0.10. For the preHD vs. earlyHD, volume’s effect sizes were r = 0.59 ± 0.02, while geometric effect sizes were 0.52 ± 0.09. Despite the small effect size, FADE was significant in HC vs. earlyHD and preHD vs. earlyHD comparisons, suggesting that striatal degeneration is reflected not only in volume and geometry but also in the internal MRI signal of the structure.

### 3.3 Group comparisons for longitudinal change

**Fig. 3.**
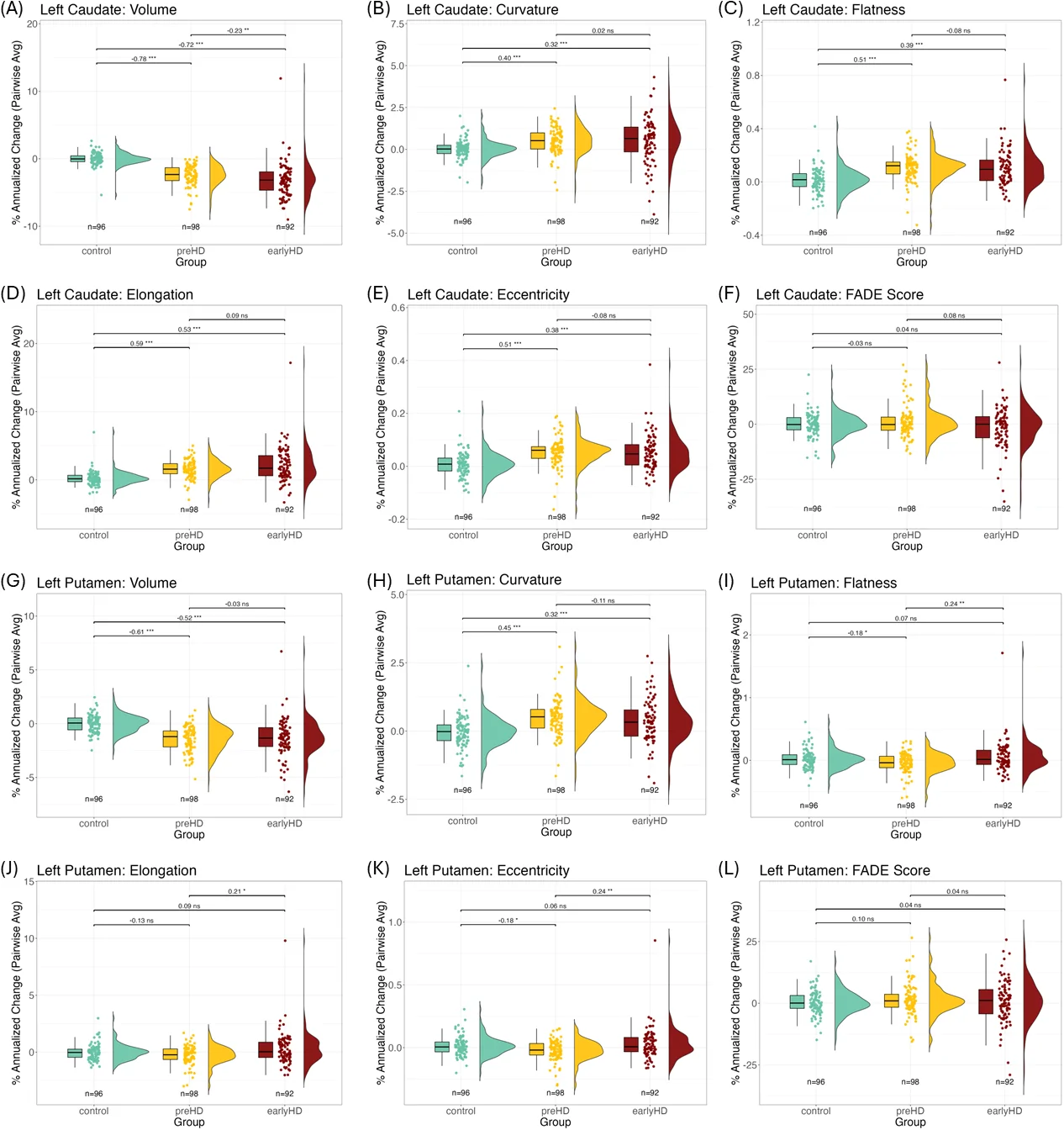
Violin plots with overlaid boxplots illustrate longitudinal group differences in six morphometric measures of the striatum: volume, curvature, flatness, elongation, eccentricity, and FADE score. Panels (A–F) show left caudate, panels (G–L) show left putamen. Groups include healthy controls (green), preHD (yellow), and earlyHD (red). Boxplots represent the median and interquartile range, while the surrounding violin distributions show kernel density estimation of individual data points (group size indicated under each distribution). Horizontal bars on top of each plot show the effect size for each pairwise comparison, marked by asterisks for Wilcoxon tests with *p < 0.05, **p < 0.01, and ***p < 0.001 (uncorrected, see FDR corrected in Table 3).

Figure 3 displays violin plots of annualized longitudinal change in left caudate and left putamen. The pattern of group differences suggests that preHD and earlyHD have somewhat similar longitudinal rates of change, which leads to their comparison showing non-significant results and tiny effect sizes. However, both HD groups showed substantial rates of longitudinal change when compared with controls, supported by large effect sizes above 0.43 in many comparisons. Flatness, elongation, and eccentricity emerged as sensitive measures of caudate change, but their sensitivity to putamen changes was more limited. Volume was clearly the most sensitive biomarker among all measures, as demonstrated by the highest effect sizes overall.

**Fig. 4.**
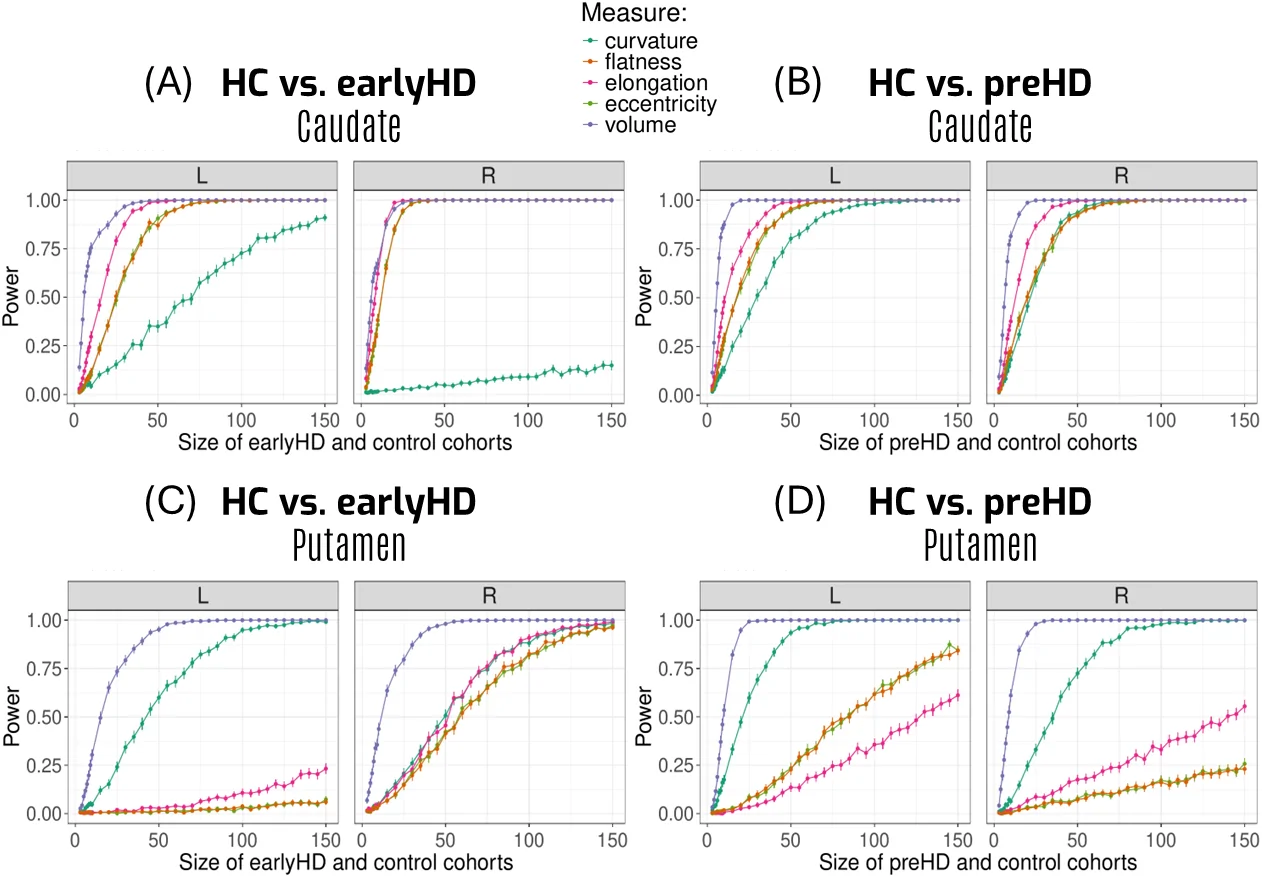
Power (probability of detecting an effect) at α = 0.05 across sample sizes per group. Sample sizes ranged from 3 to 9 subjects per group, then 10 to 150 in increments of 5. At each N, 1,000 participant-level bootstrap replicates (with replacement, balanced by group) were run; error bars show the SD across replicates. Power curves are shown for the caudate (A, B) and putamen (C, D), left (L) and right (R) hemispheres.

Figure 4 shows the statistical power curves for the key comparisons of HD groups against healthy controls in longitudinal change. The plots confirm that volume carries the highest statistical power across the range of sample sizes. The next best measure is elongation in the caudate (indicated by red lines) and curvature in putamen (indicated by green lines). Elongation and volume had equal statistical power in the HC vs. earlyHD comparison, as seen from their overlapping curves. Curvature was the more sensitive geometric measure in putamen and the least sensitive in the caudate. These results show that elongation is more sensitive to caudate changes while curvature is more sensitive to putamen changes in HD.

### 3.4 Combined biomarker potential of volume and geometric measures

**Fig. 5.**
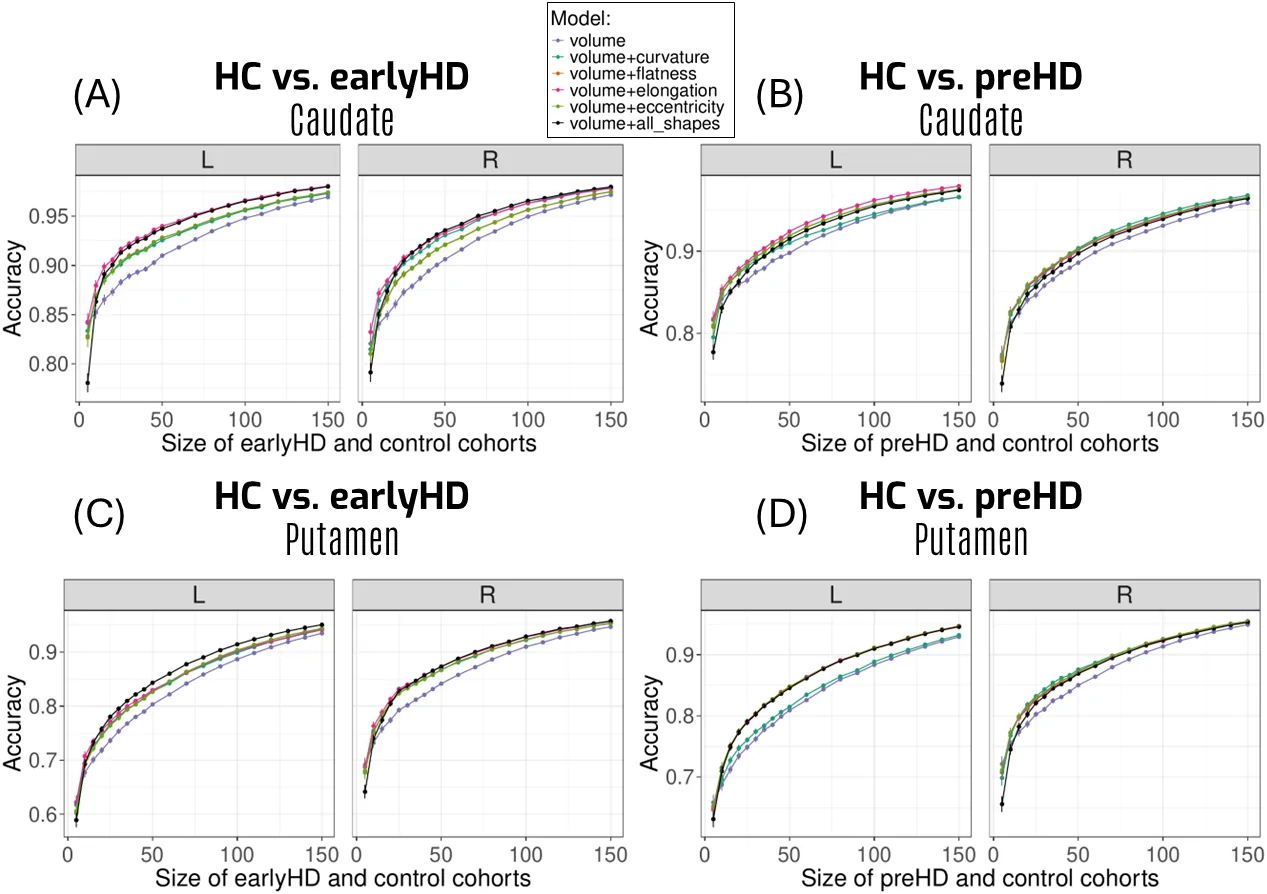
Random forest classification accuracy distinguishing HC vs preHD and HC vs earlyHD. Accuracy is obtained from out-of-bag samples of random forest models. At each sample size, from N = 5 to N = 150, random forest models were repeated 1,000 times using bootstrapping with replacement to select the groups. Sample sizes ranged from 5 to 150 subjects per group in increments of 5 up to 50, then increments of 10. Accuracy curves are shown for the caudate (A, B) and putamen (C, D), for left (L) and right (R) hemispheres.

Since volume still leads the biomarker rankings, we asked whether geometric measures could bring additional value when combined with volume. To test this, we obtained cross-validated accuracy scores from random forest models where the predictor was either volume alone or volume combined with geometric measures. Figure 5 depicts the accuracy curves obtained from HC vs. earlyHD and HC vs. preHD classification tasks.

Cross-validated accuracy improved systematically when combining volume with geometric measures. The largest improvement in the caudate emerged from the combination of volume with elongation (red lines in Fig. 5), while in putamen the largest gain was from different geometric measures, depending on the task. Combining volume with all geometric measures did not show a substantial benefit over the combination with individual scores (black lines in Fig. 5). While the gain in accuracy is not dramatic (e.g., from 90% to 95%), it can translate into smaller sample sizes in practice. For example, the HC vs. earlyHD discrimination requires N = 45/group when using left caudate volume and N = 15/group when using volume + elongation (Fig. 5A). These results indicate that geometric measures carry additional variance related to HD progression that is not captured by volume and further helps distinguish neurodegenerative processes.

### 3.5 Correlation matrices between morphometric measures

To understand the relationship between morphometric measures, we computed Pearson correlations (see “Supplementary File—Correlation Matrices”). We noticed that baseline correlations were generally higher in preHD and earlyHD than in HC, reflecting the increased collinearity due to a common degenerative process driving all striatal measures. We also noticed an extremely high correlation between flatness, elongation, and eccentricity in all plots, both cross-sectional and longitudinal (always r > 0.94). These measures, therefore, convey redundant information and may not be worth to investigate separately in the future. The FADE score was the least correlated measure with the other measures, which is not surprising given it is the only measure to rely on signal intensity rather than spatial morphometry. Lastly, we noticed that longitudinal changes in volume and geometric measures have relatively low correlation (range r = 0.17–0.40), which indicates that volume and geometric changes depend on different aspects of HD progression.

### 3.6 Isomorphic atrophy simulations

Despite the low correlation between volume and geometry in longitudinal change, one can think that geometric changes are still dependent on the size of the structure. As the structure becomes smaller, geometric properties might automatically change even when the shape is not altered. To investigate this hypothesis, we conducted controlled simulations of structural shrinkage without changing the shape, that is, isomorphic atrophy simulations. We considered the real baseline segmentation as 0% and scaled the segmentation at three levels of volume loss: 5%, 10%, and 20%. This corresponds to the amount of volume loss observed in HD participants in 1–2 years, 3–4 years, and 6–8 years, respectively (modeled from the real data used in this study). Scaling was achieved by resampling the image to 0.1 mm and changing the affine scaling factor in the file header. Resampling was an important step to achieve the desired volume shrinkage since regular 1 mm segmentations produce inconsistent volume reductions. After scaling each structure, we then measured the percent change for curvature and elongation.

**Fig. 6.**
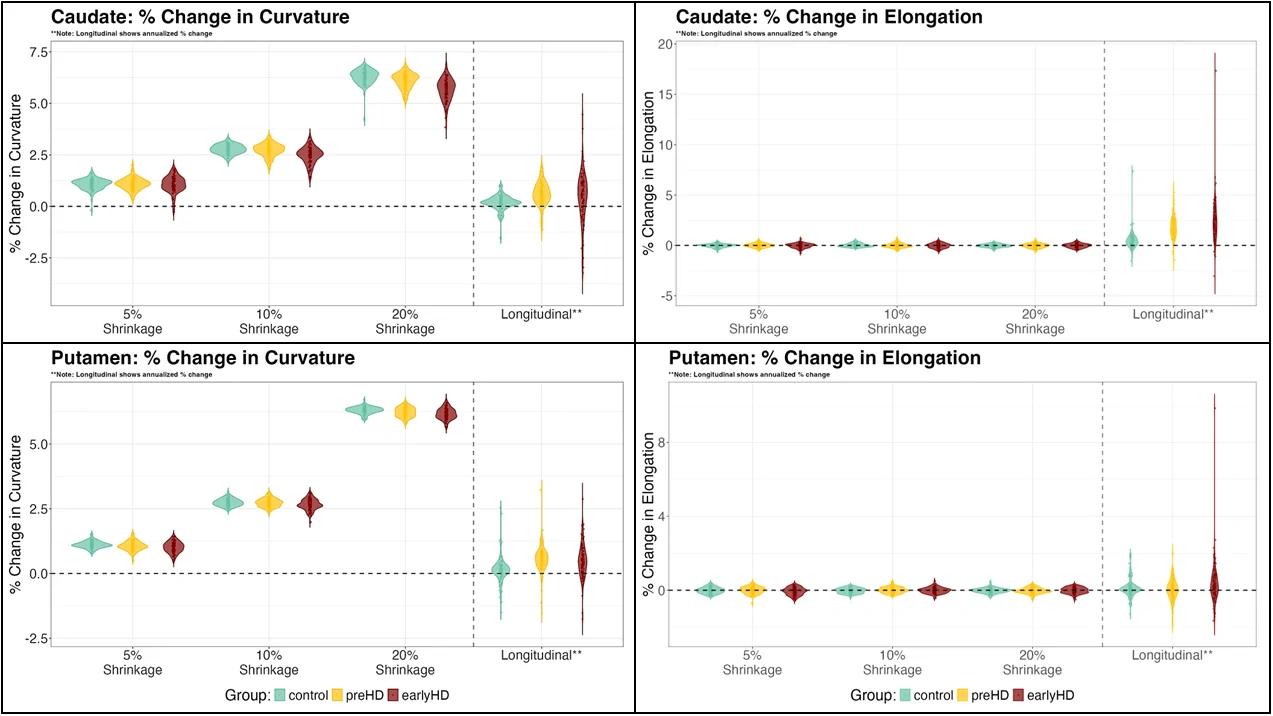
Violin plots of the change in curvature and elongation after applying controlled, isomorphic, volume shrinkage at 5%, 10%, and 20%. The “Longitudinal” section to the right of each panel shows the real annual change of the participants in the study.

Results showed that curvature increases automatically as the structure shrinks in size with a ratio of about 3.5:1 between volume decrease and curvature increase (Fig. 6). Thus, changes in curvature can depend on the size of structure even when the general shape is the same. In contrast, elongation remained static and unchanged in caudate and putamen, in all groups and at all shrinkage levels. This means that the changes in elongation observed in the real longitudinal data cannot be the result of simple shrinkage of the structure but reflect deformation of the shape of striatum of HD individuals.

### 3.7 Is the caudate–ventricle wall more useful than the full caudate?

The comparison of effect sizes from the caudate–ventricle and whole caudate is shown in “Supplementary File—Caudate vs. CaudVentWall”. There was a general loss in effect size when switching from whole caudate to caudate–ventricle wall. In cross-sectional comparisons, the average loss was r = -0.07 ± 0.10, while in longitudinal comparisons the average loss was r = -0.11 ± 0.10). In longitudinal comparisons, the loss in effect size was prominent and widespread on all comparisons. These results show that the caudate–ventricle does not provide more sensitive markers of degeneration than the whole caudate itself, particularly in longitudinal measurements, which are the most valuable in clinical trials.

### 3.8 Additional plots and analyses

To obtain a better visualization of the data, we created spaghetti plots that show the evolution of each measure by age or by CAP score, colored by group. These plots are given in “Supplementary File—Spaghetti Plots by Age” and “Supplementary File—Spaghetti Plots by CAP score”. They show a clear separation between groups for most of the measures, although some measures show more noise in a particular group (i.e., see curvature in the caudate of earlyHD). The FADE score showed poor visual separation between groups, which explains the low effect sizes reported in Table 3.

We report also linear mixed models (LMMs) as an alternative to Wilcoxon tests. LMMs are well suited to account for the different number of visits between groups, as in our data, but they do not give the opportunity to compare effect sizes. Moreover, longitudinal Wilcoxon tests compare the amount of percent change, while LMMs compare the slopes of raw scores regardless of the baseline level. Two groups may have the same apparent volume loss (e.g., 100ml) but the amount of percent loss would be larger in the group with a smaller structure. Regardless of the differences between these tests, we report the LMM results for future reference in “Supplementary File—Linear Mixed Models”.

Finally, the file “Supplementary Video—3D rendering.mp4” shows 3D visualizations of structures at the 5^th^ and 95^th^ percentile of the distribution to provide a clear understanding on how low and high volume, curvature, and elongation look like.

## 4 Discussion

In this study, we show that simple geometric descriptors of striatal shape—curvature, flatness, elongation, eccentricity—exhibit systematic relationships with HD progression, confirming our informal observations. When appended to volume, these geometric features improve group discrimination accuracy, especially in modest sample size settings. Using correlation matrices and atrophy simulations, we demonstrated that striatal degeneration in HD is not a purely volumetric process, but also a geometric one. The striatum does not simply shrink, it changes shape.

Volume loss has become the most robust imaging biomarker in HD, detectable even in premanifest stages and associated with longitudinal clinical metrics (e.g. UHDRS, CAP) (Kinnunen et al., 2021). However, volume scores face limitations: they carry no information about *where* loss occurs or *how* shape deforms. Once the atrophy becomes severe, additional volume loss becomes increasingly more difficult to measure reliably due to limitations in image resolution. Shape and deformation analysis has proved useful in other neurodegenerative diseases—for example, hippocampal shape modeling in Alzheimer’s disease—at times delivering discriminative value beyond volume (Márquez & Yassa, 2019). In HD studies, prior vertex-wise deformation studies have mapped localized inward displacements in the caudate and putamen (Kim et al., 2017; van den Bogaard et al., 2011; Wilkes et al., 2023; Younes et al., 2014), exhibiting even higher sensitivity than volume in one study (Tang et al., 2019). However, this approach yields outputs that cannot be easily reduced to single scalar biomarkers. Unlike displacements of single vertices, global geometric metrics provide a single, interpretable value that can be incorporated directly in clinical trials. Most importantly, geometric measures can be obtained from the same segmentation used to measure volume with minimal computational cost.

Through various analyses, we proved that geometric changes are not a mere reflection of volume loss. The low longitudinal correlations and the isomorphic atrophy simulations revealed that structural flattening is an independent process from volume loss. Various mechanisms might be responsible for these geometric deformations. Neuropathological studies highlight early degeneration in the dorsal caudate (Hedreen et al., 2024; Vonsattel et al., 1985), which may contribute to the collapse of the medial portion of this structure, giving the caudate a flatter, more elongated shape. The preferential degeneration of the dorsomedial sections of the caudate is reported also in voxel-based morphometry (Douaud et al., 2006; Kassubek et al., 2004) and surface displacement studies (Kim et al., 2017; van den Bogaard et al., 2011). The tail of the caudate is also selectively vulnerable in HD (Vonsattel, 2008; Vonsattel et al., 1985) although it is rather difficult to segment in MRI scans. The degeneration of the caudate tail may lead to gradually more spherical shape of posterior caudate in neuroimaging segmentations, increasing the curvature. In putamen, studies have shown both medial and lateral deflation that may contribute to make the putamen flatter and more elongated in HD individuals (Looi et al., 2012; Tang et al., 2019; van den Bogaard et al., 2011). Our simulations showed that changes in curvature can be attributed to volume loss. However, curvature change did not correlate well with volume change longitudinally (see “Supplementary File—Correlation Matrices”), adding to the evidence that other mechanical forces are responsible for giving caudate and putamen a rounder shape.

The detection of pathologic longitudinal change is a key feature required in clinical trials. We found that geometric measures are well suited to distinguish longitudinal anomalies in HD with large effect sizes > 0.43 (Table 3). In contrast, the direct comparison of preHD vs. earlyHD was challenging and was characterized by low effect sizes. This indicates that longitudinal degeneration in HD occurs at a similar pace both before and after clinical motor diagnosis. In general, volume showed higher longitudinal effect sizes than geometric measures, but not always. For example, elongation, flatness, and eccentricity had higher effect sizes (r = 0.27–0.30) than volume (r = 0.08–0.15) when comparing preHD vs. earlyHD in the right caudate.

The reason why a geometric measure is better capable of distinguishing different states of HD is probably related to the same reason why volume and geometric scores do not correlate well with each other. Unlike volume, which reflects only the degeneration of the measured structure, the geometry of a structure reflects a combination of mechanical forces that include the intrinsic structural consistency and the extrinsic push–pull forces of surrounding structures. More advanced HD stages are characterized by more widespread and more severe anomalies across the brain, including white-matter degeneration, loss of whole brain volume, altered CSF flow, and increase in ventricular volume (Hett et al., 2023; Hobbs et al., 2010; Tan et al., 2021; Wild et al., 2010). Together, these extra-striatal anomalies may contribute to modify the external mechanical forces exercised on the striatum, leading to geometric changes that cannot simply be explained by striatal volume loss. In other words, geometric changes may reflect not just striatal degeneration but also wider anomalies beyond the striatum itself.

The investigation of FADE showed abnormalities in earlyHD at baseline but no abnormalities in longitudinal change. While weak, these results demonstrate for the first time that direct quantification of MRI intensity may reveal processes of internal structural degeneration that are not found in volumetric measurements. Similar to FADE, VBM analyses also rely on MRI signal intensity and have shown good sensitivity in HD monitoring (Castro et al., 2018; Scahill et al., 2013; X. Wang et al., 2024). However, numerous VBM studies apply a step called “gray matter modulation” that alters VBM maps to reflect volume anomalies rather than signal intensity (Henley et al., 2010). Our findings invite researchers to reconsider the value of VBM without GM modulation for detecting the internal structural degeneracy rather than volume anomalies. The two types of striatal anomalies—volume loss and signal fading—are likely to reflect different, orthogonal signals that might be worth investigating in tandem. Our implementation of FADE was somewhat sensitive to site effects and may benefit from better intensity normalization in the future, such as WhiteStripe (Shinohara et al., 2014) or RAVEL (Fortin et al., 2016).

The inspection of effect sizes and power curves did not show systematic asymmetries. In the HC vs. earlyHD comparison, the right putamen had somewhat larger effect sizes than the left putamen for longitudinal changes in flatness, elongation, and eccentricity. Power curves showed a mixed picture of inconsistent asymmetries. To check whether one hemisphere shows stronger effect sizes, we ran paired t-tests on left and right columns of Table 3 but found no statistical difference. Thus, the two hemispheres produce similar effect sizes on average. Researchers often average left and right scores in HD, and asymmetry reports are rare (Lila et al., 2025; Minkova et al., 2018). Despite not finding asymmetries, we report left and right structures separately to provide a full picture of each individual region. In future studies, once the lack of asymmetries is confirmed with other segmentation pipelines and different datasets, left and right scores can be averaged into a single score to improve the signal-to-noise ratio.

This study constitutes the first step toward the development of geometric biomarkers. Among the five measures investigated, only two are worthy of further development. One of them is *curvature*, which showed distinct sensitivity and acceptable collinearity with other measures. The other one must be chosen from the *flatness/elongation/eccentricity* triad, which showed good biomarker sensitivity but extremely high collinearity. We recommend using elongation because it showed the best biomarking power among the three. FADE showed weak results and we do not recommend investigating it further, at least not with our computational approach.

There are three directions the development of geometric biomarkers can continue. First, it is important to investigate the relationship of geometric markers with cognitive and clinical scores. This is central for understanding to what extent geometric changes are related to clinical decline. If geometric scores reflect more than just striatal degeneration, as hypothesized above, there is a chance that geometric scores may correlate with clinical scores better than volume. Second, it is important to replicate the current findings with different segmentation pipelines. FreeSurfer segmentations have both advantages and disadvantages. The main advantage is the longitudinal pipeline that reduces sudden changes in striatal segmentation and increases the sensitivity to longitudinal change (Reuter et al., 2012). This approach has the disadvantage of needing to re-process over every time the participant undergoes a new MRI visit, updating the morphometric values and adding uncertainty in clinical-trial settings. On another note, the quality of subcortical segmentations with FreeSurfer is less than optimal. We have noticed that portions of the claustrum are routinely mis-segmented as putamen, creating an artificial bulge in the lateral surface of the putaminal shape (Knights et al., 2024; Pustina et al., 2024). Mansoor et al. (2021) conducted a thorough comparison of various pipelines and found that FreeSurfer segments healthy brains more accurately than HD brains, likely due to the use of a healthy population template. Segmentations from other pipelines may improve the sensitivity of geometric measures further. Third, while the data used in this study are of good quality, is important to replicate the findings with other datasets, some of which have more heterogeneous scans across a larger number of sites (e.g., PREDICT-HD).

This study provided robust evidence but still carries several limitations. First, one of the groups, earlyHD, had less longitudinal timepoints and potentially more noise in longitudinal measurements than the other two. Despite this, earlyHD showed a clear separation from healthy controls both in violin plots and in statistical results, suggesting that the expected increase in noise is not dramatic. Second, we used baseline as reference to compute longitudinal change for all future timepoints. Any noise or mis-segmentation at baseline would propagate to all longitudinal changes, creating a heavy influence of baseline scores. The FreeSurfer longitudinal pipeline helps to avoid this issue by regularizing longitudinal segmentations to prevent unreasonable changes in the segmentation mask. The plots in “Supplementary File—Spaghetti Plots by Age” show gradual longitudinal progression for most subjects. Averaging longitudinal changes may also miss non-linear changes over time, that is, accelerations at specific timepoints. Third, groups were defined based on the participant’s baseline status without considering whether some preHD participants progressed to earlyHD. However, the different initial starting point in the disease progression spectrum should still anchor group comparisons on different segments of the progression. Fourth, we did not harmonize the data across sites or explicitly model the scanner-related effects, which could have reduced the measurement variability further. Lastly, we corrected geometric scores for intracranial volume, while a more principled approach would be to correct local geometry (striatum) for the global geometry (whole brain). Yet, the whole brain geometry is likely a moving target given that HD affects the entire brain (Kinnunen et al., 2021). Future studies can test more rigorously the influence of whole brain shape (or perhaps intracranial shape) on caudate and putamen shapes alongside better site harmonization approaches.

## 5 Conclusions

Huntington’s disease leaves two signatures on the striatum: loss of volume and alteration of shape. While volume remains the strongest standalone biomarker, geometric measures provide additional information that sharpens disease detection and tracking. Measuring both may offer a more complete picture of neurodegeneration than either alone.

## Supplementary Material

Supplementary Material

Supplementary Video - 3D rendering

Supplementary Video - Caudate Ventricle Wall

## Data Availability

The source datasets used in this study are available upon request, please send requests to info@chdifoundation.org. Dataset references: DATA-00000929, DATA-00000931, DATA-00001095.
