# Supplementary material for: Geometric properties of caudate and putamen mark the progression of Huntington’s disease
Source: Imaging Neurosci (Camb). 2026 Aug 24;4:IMAG.a.1351. doi: 10.1162/IMAG.a.1351 (PMC13504955; doi:10.1162/IMAG.a.1351)
Supplement: Supplementary file 1 [file IMAG.a.1351_supp.pdf]

Supplementary File - Example Code

```
####################################################
##  EXAMPLE CODE TO CALCULATE GEOMETRIC MEASURES   ##
####################################################


# Requires antspy 0.5.4 , curvanato 0.1.0
# Load freesurfer segmentation
mri_convert norm.mgz norm.nii.gz
mri_convert aparc.DKTatlas+aseg.mgz aparc.DKTatlas+aseg.nii.gz

origseg = aparc.DKTatlas+aseg.nii.gz
# In python
# Resample
segmentation = ants.resample_image( origseg, [0.5,0.5,0.5], interp_type=1 )

targetlabel = [replace with appropriate DKT label value]
# Extract label
binaryimage = ants.threshold_image(segmentation, target_label,
target_label).iMath("FillHoles").iMath("GetLargestComponent")

# Compute curvature
curvit = compute_curvature( binaryimage, smoothing=smoothing, distance_map = True )

# Create dataframe
mydf = make_label_dataframe( binaryimage )

# Add curvature to dataframe
bindescriptor = antspyt1w.map_intensity_to_dataframe( mydf, curvit, binaryimage )

# Compute other shape measures and add to dataframe
# Compute flatness and add
bindescriptor = compute_geom_per_label( binaryimage, bindescriptor, flatness, 'Flatness')

# Compute volume, surface area, eccentricity and add
gdesc = ants.label_geometry_measures( binaryimage )
geos = ['Mean', 'VolumeInMillimeters', 'SurfaceAreaInMillimetersSquared', 'Eccentricity',
'Elongation','Flatness']
bindescriptor = pd.concat([bindescriptor.reset_index(drop=True), gdesc.reset_index(drop=True)],
axis=1)
bindescriptor = pd_to_wide( bindescriptor, column_values=geos, prefix='CaudKAPWhole_')

# Write output CSV file with shape measures
bindescriptor.to_csv('shape_measures.csv')



############################################
##  EXAMPLE CODE TO CALCULATE FADE SCORE   ##
############################################

# Requires c3d

# Define parameters
MINPERC=10
MAXPERC=100
MINSTDBG=2
MINSTDWM=5
MINSTDGM=10
MAXSTDBG=0
MAXSTDWM=95
MAXSTDGM=90
FILTERMODE=prc


# Load freesurfer segmentation
mri_convert norm.mgz norm.nii.gz
mri_convert aparc.DKTatlas+aseg.mgz SEG.nii.gz

targetlabel = [replace with appropriate DKT label value]
```

```
# Extract label
c3d SEG.nii.gz -thresh targetlabel targetlabel -o ROI.nii.gz

# Create modified mask and compute intensity distribution of the basal ganglia region
filter_seg norm.nii.gz SEG.nii.gz ROI.nii.gz ROI.nii.gz SEG.nii.gz $MINSTDBG $MAXSTDBG $ROI
$FILTERMODE
python roiint.py T1.nii.gz ROI.nii.gz ${ROI}_intensity.txt ${ROI}_hist.png $ROI

# Get eroded white matter mask
c3d SEG.nii.gz -replace 2 41 -o SEG.nii.gz -thresh 41 41 1 0 -erode 1 1x1x1vox -o WM.nii.gz
# Create modified mask and compute intensity distribution of the basal ganglia region
filter_seg T1.nii.gz SEG.nii.gz WM.nii.gz WM.nii.gz SEG.nii.gz $MINSTDWM $MAXSTDWM 41 prc
python roiint.py T1.nii.gz WM.nii.gz WM_intensity.txt WM_hist.png WM

# Get selective cortical GM mask
CTXLBLREPCMD="-replace 1002 10000 1003 10000 1005 10000 1006 10000 1007 10000 1008 10000 1009
10000 1010 10000 1011 10000 1012 10000 1013 10000 1014 10000 1015 10000 1016 10000 1017 10000
1018 10000 1019 10000 1020 10000 1023 10000 1025 10000 1026 10000 1027 10000 1028 10000 1029
10000 1030 10000 1031 10000 1034 10000 1035 10000 2002 10000 2003 10000 2005 10000 2006 10000
2007 10000 2008 10000 2009 10000 2010 10000 2011 10000 2012 10000 2013 10000 2014 10000 2015
10000 2016 10000 2017 10000 2018 10000 2019 10000 2020 10000 2023 10000 2025 10000 2026 10000
2027 10000 2028 10000 2029 10000 2030 10000 2031 10000 2034 10000 2035 10000"
c3d SEG.nii.gz $CTXLBLREPCMD -o SEG.nii.gz -thresh 10000 10000 1 0 -o CTX.nii.gz
# Create modified mask and compute intensity distribution of cortical GM
filter_seg T1.nii.gz SEG.nii.gz CTX.nii.gz CTX.nii.gz SEG.nii.gz $MINSTDGM $MAXSTDGM 10000 prc
python roiint.py T1.nii.gz CTX.nii.gz CTX_intensity.txt CTX_hist.png CTX

# Compute FADE score
python pilotscore.py CTX_intensity.txt ${ROI}_intensity.txt WM_intensity.txt > fadescore.txt
```

Supplementary File - Normality and Variance Tests

Tables of Shapiro-Wilk tests of normality (above) and Levene's tests of variance heteroscedasticity (below). Cells in red mark significant tests that violate the assumptions of typical parametric group comparisons. Cells in green mark tests that are not significant.

		Cross-sect. Shapiro W (Normality)				Longitudinal Shapiro W (Normality)			
		Caudate		Putamen		Caudate		Putamen	
		Left	*Right*	*Left*	*Right*	*Left*	*Right*	*Left*	*Right*
**HC**	Volume	0.988	0.994	0.986	0.99	0.861	0.971	0.988	0.972
	Curvature	0.981	0.98	0.983	0.992	0.933	0.967	0.951	0.957
	Flatness	0.987	0.98	0.95	0.977	0.951	0.98	0.953	0.904
	Elongation	0.955	0.973	0.977	0.991	0.798	0.976	0.933	0.937
	Eccentricity	0.987	0.98	0.948	0.976	0.951	0.98	0.954	0.904
	FADE	0.974	0.99	0.989	0.992	0.936	0.976	0.981	0.986
**preHD**	Volume	0.987	0.975	0.989	0.994	0.953	0.945	0.979	0.97
	Curvature	0.96	0.96	0.974	0.963	0.961	0.989	0.945	0.98
	Flatness	0.979	0.985	0.986	0.986	0.933	0.911	0.951	0.796
	Elongation	0.903	0.926	0.992	0.981	0.985	0.957	0.952	0.849
	Eccentricity	0.979	0.985	0.985	0.986	0.933	0.911	0.951	0.794
	FADE	0.987	0.981	0.926	0.91	0.903	0.889	0.931	0.905
**earlyHD**	Volume	0.981	0.984	0.984	0.984	0.87	0.934	0.922	0.913
	Curvature	0.984	0.991	0.992	0.985	0.971	0.911	0.971	0.992
	Flatness	0.963	0.963	0.989	0.984	0.908	0.919	0.754	0.927
	Elongation	0.993	0.995	0.972	0.983	0.855	0.924	0.763	0.91
	Eccentricity	0.963	0.964	0.988	0.984	0.907	0.919	0.755	0.927
	FADE	0.96	0.956	0.98	0.973	0.93	0.956	0.971	0.96

		Cross-sect. Levene's F (unequal variances)				Longitudinal Levene's F (unequal variances)			
		Caudate		Putamen		Caudate		Putamen	
		Left	*Right*	*Left*	*Right*	*Left*	*Right*	*Left*	*Right*
**HC vs. earlyHD**	Volume	6.436	5.181	0.201	1.044	31.537	27.879	17.797	16.569
	Curvature	16.427	18.861	28.427	49.721	32.836	33.156	5.758	13.463
	Flatness	1.22	1.395	2.694	8.786	7.359	1.783	4.359	4.058
	Elongation	57.732	47.127	24.899	46.833	29.392	26.195	12.195	15.033
	Eccentricity	1.104	1.305	2.316	7.885	7.378	1.765	4.387	4.048
	FADE	20.1	31.751	24.397	20.646	10.065	12.427	15.957	11.647
**HC vs. preHD**	Volume	4.602	2.199	5.074	4.855	18.281	18.157	11.934	11.151
	Curvature	8.064	5.911	18.454	20.102	10.602	12.326	0.882	3.61
	Flatness	5.939	6.864	0.002	0.629	0.552	1.316	0.592	0.028
	Elongation	10.994	11.859	1.298	8.578	6.543	10.793	2.196	0.395
	Eccentricity	5.825	6.808	0	0.522	0.54	1.303	0.605	0.028
	FADE	2.332	6.515	0.901	2.192	2.694	0.912	1.678	2.5
**preHD vs. earlyHD**	Volume	0.27	0.557	3.053	1.57	9.25	3.721	3.209	1.139
	Curvature	0.854	2.924	0.868	5.526	12.401	17.512	2.115	3.432
	Flatness	1.6	2.101	3.114	5.537	3.575	0.027	2.478	2.894
	Elongation	13.348	7.387	16.434	22.813	15.859	7.747	6.653	10.072
	Eccentricity	1.703	2.185	2.833	5.097	3.613	0.026	2.471	2.878
	FADE	11.841	12.05	12.66	7.372	2.432	6.639	6.675	2.963

Supplementary File - Cohens D Effect Sizes

Tables with parametric (Cohen's D) effect sizes.

Please excercise caution when comparing these values with other studies. The assumptions of normality and variance homogeneity may be violated for certain group contrasts (see other supplementary tables of Shapiro and Levene tests). Bold fonts show significant Wilcoxon tests at $p < 0.05$ (FDR corrected).

		Cross-sect.Cohen's D			
		Caudate		Putamen	
		Left	*Right*	*Left*	*Right*
HC vs. earlyHD	Volume	**2.99**	**3.00**	**3.74**	**3.67**
	Curvature	**2.77**	**2.61**	**3.21**	**3.16**
	Flatness	**2.57**	**2.64**	**1.50**	**1.84**
	Elongation	**2.39**	**2.57**	**1.43**	**1.67**
	Eccentricity	**2.56**	**2.62**	**1.49**	**1.83**
	FADE	**0.64**	**0.64**	**0.76**	**0.51**
HC vs. preHD	Volume	**1.58**	**1.64**	**1.85**	**1.81**
	Curvature	**1.03**	**1.02**	**1.58**	**1.61**
	Flatness	**1.01**	**1.09**	**0.72**	**0.81**
	Elongation	**0.92**	**0.99**	**0.83**	**0.94**
	Eccentricity	**1.01**	**1.09**	**0.71**	**0.80**
	FADE	*0.04*	*0.02*	*0.12*	*0.04*
preHD vs. earlyHD	Volume	**1.37**	**1.34**	**1.45**	**1.47**
	Curvature	**1.52**	**1.51**	**1.44**	**1.53**
	Flatness	**1.27**	**1.22**	**0.87**	**1.12**
	Elongation	**1.47**	**1.49**	**0.78**	**0.98**
	Eccentricity	**1.25**	**1.21**	**0.87**	**1.12**
	FADE	**0.65**	**0.59**	**0.62**	**0.44**

		Longitudinal Cohen's D			
		Caudate		Putamen	
		Left	*Right*	*Left*	*Right*
HC vs. earlyHD	Volume	**1.58**	**1.66**	**1.00**	**1.15**
	Curvature	**0.49**	**0.19**	**0.62**	**0.58**
	Flatness	**0.80**	**1.23**	*0.17*	**0.52**
	Elongation	**1.00**	**1.54**	*0.25*	**0.57**
	Eccentricity	**0.80**	**1.23**	*0.17*	**0.52**
	FADE	*0.16*	*0.09*	*0.08*	*0.01*
HC vs. preHD	Volume	**1.97**	**1.76**	**1.44**	**1.47**
	Curvature	**0.73**	**0.90**	**0.87**	**0.69**
	Flatness	**0.96**	**0.90**	**0.44**	**0.23**
	Elongation	**1.17**	**1.17**	*0.35*	**0.33**
	Eccentricity	**0.96**	**0.90**	**0.44**	**0.23**
	FADE	*0.19*	*0.24*	*0.23*	*0.23*
preHD vs. earlyHD	Volume	**0.32**	**0.26**	*0.08*	*0.13*
	Curvature	*0.00*	*0.23*	*0.16*	*0.03*
	Flatness	*0.03*	*0.29*	**0.50**	*0.29*
	Elongation	*0.29*	**0.65**	**0.47**	*0.31*
	Eccentricity	*0.03*	*0.29*	**0.50**	*0.29*
	FADE	*0.29*	*0.26*	*0.10*	*0.17*

Supplementary File - Descriptive Statistics

Tables of descriptive statistics per measure, region, hemisphere, and group.

**Column**	**Description**
Measure	Measure
Region	caudate or putamen
Hemisphere	L (left) or R (right)
Group	control, preHD, or earlyHD
N	Number of subjects in this group/region/hemisphere combination
Mean	Arithmetic mean of the measure across subjects
SD	Standard deviation; spread of values around the mean
Median	Middle value when subjects are ranked
Min	Smallest observed value
Max	Largest observed value
Q1	25th percentile; value below which 25% of subjects fall
Q3	75th percentile; value below which 75% of subjects fall
Skewness	Asymmetry of the distribution. Zero = symmetric. Positive = long right tail. Negative = long left tail. Values above 1 or below −1 can indicate meaningful departure from symmetry
Kurtosis	Heaviness of the tails relative to a normal distribution. Normal distribution = 3. Values substantially above 3 can indicate heavier tails and more extreme outliers than expected
N_outliers	Total number of outliers detected using the Tukey 1.5 × IQR rule (values beyond Q1 − 1.5×IQR or Q3 + 1.5×IQR)
N_outliers_low	Number of outliers below the lower fence
N_outliers_high	Number of outliers above the upper fence
Outlier_pct	Outliers as a percentage of N

Cross-Sectional Scores - Descriptive Statistics

Measure	Region	Hemisphere	Group	N	Mean	SD	Median	Min	Max	Q1	Q3	Skewness	Kurtosis	N_outliers	N_outliers_low	N_outliers_high	Outlier_pct
Volume	caudate	L	control	100	-739.1704	369.3936	-760.4815	-1573.84	85.5575	-952.5016	-459.2087	0.026	2.644	0	0	0	0
Volume	caudate	L	preHD	100	-1396.506	459.4597	-1397.823	-2625.587	-350.7756	-1636.512	-1089.353	-0.345	2.933	2	2	0	2
Volume	caudate	L	earlyHD	100	-2053.402	499.9797	-2054.666	-3050.532	-726.3992	-2353.23	-1757.236	0.299	3.102	3	0	3	3
Volume	caudate	R	control	100	-630.0274	375.5224	-613.724	-1530.906	351.5575	-872.2879	-328.5226	-0.017	2.823	0	0	0	0
Volume	caudate	R	preHD	100	-1306.011	445.995	-1207.928	-2369.187	-201.0756	-1543.706	-1033.828	-0.335	2.824	3	2	1	3
Volume	caudate	R	earlyHD	100	-1933.884	487.1458	-1953.736	-2972.032	-574.7992	-2222.565	-1579.997	0.212	2.984	1	0	1	1
Volume	putamen	L	control	100	767.5796	497.7112	772.8005	-307.4058	1918.1575	410.4584	1128.2842	0.102	2.267	0	0	0	0
Volume	putamen	L	preHD	100	-306.6354	654.5983	-288.7666	-1874.007	1045.765	-790.5289	146.6439	-0.084	2.461	0	0	0	0
Volume	putamen	L	earlyHD	100	-1175.625	541.3622	-1194.648	-2414.132	335.3862	-1630.687	-809.3633	0.331	2.871	0	0	0	0

Volume	putamen	R	control	100	825.5706	505.7096	821.2094	-278.7058	2271.5097	483.6396	1189.9426	0.242	2.659	1	0	1	1
Volume	putamen	R	preHD	100	-237.5574	656.99	-214.1708	-1894.607	1238.7244	-683.7052	163.1863	-0.078	2.705	0	0	0	0
Volume	putamen	R	earlyHD	100	-1134.876	560.0765	-1160.4	-2292.432	402.3862	-1580.676	-708.99	0.235	2.653	0	0	0	0
Curvature	caudate	L	control	100	0.0161	0.0065	0.0156	0.0027	0.037	0.0112	0.0203	0.494	3.103	1	0	1	1
Curvature	caudate	L	preHD	100	0.0246	0.0096	0.0231	0.0059	0.0521	0.0184	0.0287	0.669	3.327	5	0	5	5
Curvature	caudate	L	earlyHD	100	0.0396	0.01	0.0415	0.0171	0.0661	0.0313	0.0453	-0.001	2.501	0	0	0	0
Curvature	caudate	R	control	100	0.0124	0.0065	0.0109	-0.0016	0.0306	0.0076	0.0172	0.396	2.803	0	0	0	0
Curvature	caudate	R	preHD	100	0.0204	0.009	0.0188	0.0042	0.0477	0.0144	0.0253	0.676	3.051	1	0	1	1
Curvature	caudate	R	earlyHD	100	0.0351	0.0105	0.0349	0.0092	0.062	0.0268	0.0417	0.125	2.668	0	0	0	0
Curvature	putamen	L	control	100	-0.0141	0.0062	-0.0144	-0.0253	3.00E-04	-0.0192	-0.0102	0.211	2.442	0	0	0	0
Curvature	putamen	L	preHD	100	-4.00E-04	0.0106	-0.0017	-0.023	0.0284	-0.0084	0.0056	0.475	2.745	1	0	1	1
Curvature	putamen	L	earlyHD	100	0.0156	0.0116	0.0151	-0.0145	0.0425	0.0088	0.0227	-0.045	2.703	1	1	0	1
Curvature	putamen	R	control	100	-0.0165	0.0059	-0.0165	-0.0323	-0.0034	-0.0201	-0.0132	0.022	2.92	1	1	0	1
Curvature	putamen	R	preHD	100	-0.003	0.0103	-0.0045	-0.0219	0.0306	-0.0109	0.0032	0.72	3.5	2	0	2	2
Curvature	putamen	R	earlyHD	100	0.0147	0.0126	0.014	-0.0123	0.0436	0.0049	0.0234	0.19	2.402	0	0	0	0
Flatness	caudate	L	control	100	0.0264	0.0074	0.0259	0.0116	0.0442	0.0212	0.0313	0.248	2.702	0	0	0	0
Flatness	caudate	L	preHD	100	0.0353	0.01	0.0344	0.0055	0.058	0.0288	0.0423	0.044	3.332	1	1	0	1
Flatness	caudate	L	earlyHD	100	0.0471	0.0087	0.0486	0.0183	0.0681	0.0417	0.0535	-0.737	3.849	1	1	0	1
Flatness	caudate	R	control	100	0.0265	0.0066	0.0264	0.0142	0.0475	0.0218	0.0319	0.29	3.064	1	0	1	1
Flatness	caudate	R	preHD	100	0.0353	0.0091	0.0342	0.0131	0.0594	0.0286	0.0404	0.265	3.06	1	0	1	1
Flatness	caudate	R	earlyHD	100	0.0456	0.0077	0.0463	0.0197	0.0602	0.041	0.0517	-0.743	3.451	1	1	0	1
Flatness	putamen	L	control	100	-0.0263	0.0135	-0.0237	-0.0704	-0.0032	-0.0332	-0.0166	-0.891	3.779	3	3	0	3
Flatness	putamen	L	preHD	100	-0.017	0.0126	-0.0167	-0.0491	0.0113	-0.0254	-0.0075	-0.29	2.589	0	0	0	0
Flatness	putamen	L	earlyHD	100	-0.0047	0.0155	-0.0039	-0.0503	0.0285	-0.0153	0.006	-0.323	2.921	1	1	0	1
Flatness	putamen	R	control	100	-0.0307	0.0119	-0.0292	-0.0682	-0.006	-0.0379	-0.0209	-0.54	3.171	1	1	0	1
Flatness	putamen	R	preHD	100	-0.0209	0.0124	-0.0205	-0.0533	0.0052	-0.0308	-0.0109	-0.185	2.414	0	0	0	0
Flatness	putamen	R	earlyHD	100	-0.0047	0.0161	-0.0014	-0.0445	0.0301	-0.0154	0.0063	-0.223	2.507	0	0	0	0
Elongation	caudate	L	control	100	0.6798	0.3462	0.6367	0.1201	1.9342	0.436	0.8731	0.848	3.975	3	0	3	3
Elongation	caudate	L	preHD	100	1.1461	0.6243	0.9999	-0.0452	3.9088	0.7474	1.3977	1.347	5.98	4	0	4	4
Elongation	caudate	L	earlyHD	100	2.2496	0.8622	2.2033	0.2841	4.3417	1.6699	2.8474	0.13	2.565	0	0	0	0
Elongation	caudate	R	control	100	0.6836	0.308	0.6772	0.1765	1.511	0.4674	0.866	0.455	2.653	1	0	1	1
Elongation	caudate	R	preHD	100	1.1197	0.538	0.9909	0.1221	2.836	0.7215	1.3042	1.025	3.979	6	0	6	6
Elongation	caudate	R	earlyHD	100	2.0281	0.6725	2.0317	0.3575	3.6424	1.5771	2.4498	-0.067	2.704	0	0	0	0
Elongation	putamen	L	control	100	-0.7008	0.2098	-0.6952	-1.3064	-0.3219	-0.8444	-0.5163	-0.415	2.832	0	0	0	0
Elongation	putamen	L	preHD	100	-0.5182	0.2315	-0.5226	-1.1095	0.0683	-0.7055	-0.3535	0.074	2.563	0	0	0	0
Elongation	putamen	L	earlyHD	100	-0.2856	0.3541	-0.2763	-0.9293	0.7306	-0.5759	-0.0624	0.5	2.923	1	0	1	1
Elongation	putamen	R	control	100	-0.7679	0.1724	-0.764	-1.2629	-0.3445	-0.8817	-0.6617	-0.263	3.004	1	1	0	1
Elongation	putamen	R	preHD	100	-0.5835	0.2179	-0.5792	-0.9735	-0.0848	-0.7528	-0.4268	0.076	2.23	0	0	0	0
Elongation	putamen	R	earlyHD	100	-0.286	0.3692	-0.2934	-1.0876	0.788	-0.5857	-0.0386	0.36	2.858	1	0	1	1
Eccentricity	caudate	L	control	100	0.0138	0.0038	0.0136	0.0061	0.0229	0.0111	0.0163	0.247	2.707	0	0	0	0
Eccentricity	caudate	L	preHD	100	0.0183	0.0051	0.0179	0.003	0.0298	0.0149	0.022	0.035	3.336	1	1	0	1
Eccentricity	caudate	L	earlyHD	100	0.0243	0.0045	0.0249	0.0096	0.0351	0.0215	0.0276	-0.731	3.85	1	1	0	1
Eccentricity	caudate	R	control	100	0.0138	0.0034	0.0137	0.0075	0.0247	0.0114	0.0166	0.299	3.092	1	0	1	1

Eccentricity	caudate	R	preHD	100	0.0183	0.0047	0.0178	0.0069	0.0307	0.0149	0.021	0.262	3.062	1	0	1	1
Eccentricity	caudate	R	earlyHD	100	0.0235	0.004	0.0239	0.0104	0.0311	0.0212	0.0267	-0.728	3.426	1	1	0	1
Eccentricity	putamen	L	control	100	-0.0137	0.0071	-0.0123	-0.0372	-0.0016	-0.0173	-0.0086	-0.916	3.845	3	3	0	3
Eccentricity	putamen	L	preHD	100	-0.0088	0.0067	-0.0086	-0.0257	0.006	-0.0132	-0.0038	-0.305	2.599	0	0	0	0
Eccentricity	putamen	L	earlyHD	100	-0.0024	0.0081	-0.0019	-0.0265	0.0148	-0.0079	0.0032	-0.351	2.961	1	1	0	1
Eccentricity	putamen	R	control	100	-0.016	0.0063	-0.0152	-0.036	-0.003	-0.0199	-0.0108	-0.556	3.19	1	1	0	1
Eccentricity	putamen	R	preHD	100	-0.0109	0.0066	-0.0106	-0.0281	0.0028	-0.0161	-0.0056	-0.2	2.43	0	0	0	0
Eccentricity	putamen	R	earlyHD	100	-0.0024	0.0084	-6.00E-04	-0.0233	0.0157	-0.008	0.0033	-0.239	2.514	0	0	0	0
FADE Score	caudate	L	control	100	-513.9068	271.6336	-543.7143	-978.4263	103.4245	-700.2896	-356.508	0.301	2.511	0	0	0	0
FADE Score	caudate	L	preHD	100	-526.3531	303.0184	-527.6958	-1242.709	100.8973	-766.5797	-304.9042	0.033	2.308	0	0	0	0
FADE Score	caudate	L	earlyHD	100	-264.7128	479.1892	-287.5884	-1091.821	1199.9475	-628.0767	-26.1554	0.716	3.375	3	0	3	3
FADE Score	caudate	R	control	100	-465.9522	264.2673	-484.853	-1132.108	132.4401	-628.314	-300.7088	0.028	2.853	1	1	0	1
FADE Score	caudate	R	preHD	100	-459.016	337.021	-525.8807	-1173.773	553.144	-682.2536	-205.3093	0.357	2.811	1	0	1	1
FADE Score	caudate	R	earlyHD	100	-208.5771	499.9774	-282.5437	-1000.772	1543.8126	-586.6235	122.3881	0.742	3.799	2	0	2	2
FADE Score	putamen	L	control	100	634.8784	514.377	630.4631	-558.5683	1963.3495	247.3456	987.2377	0.233	2.662	0	0	0	0
FADE Score	putamen	L	preHD	100	705.6938	633.3263	635.1052	-336.7406	3060.8762	262.3245	984.5629	1.137	4.643	4	0	4	4
FADE Score	putamen	L	earlyHD	100	1167.3764	847.689	1080.3232	-439.0486	3377.709	495.1268	1739.5265	0.318	2.484	0	0	0	0
FADE Score	putamen	R	control	100	566.7417	430.6367	592.776	-610.056	1489.276	255.0771	863.8192	-0.067	2.682	0	0	0	0
FADE Score	putamen	R	preHD	100	584.9845	561.1522	542.7328	-390.5428	3550.8355	202.671	867.5725	1.521	9.28	1	0	1	1
FADE Score	putamen	R	earlyHD	100	863.2794	698.4353	853.7499	-330.6301	3368.8108	308.9574	1388.4974	0.472	3.392	1	0	1	1

Longitudinal Scores - Descriptive Statistics

Measure	Region	Hemisphere	Group	N	Mean	SD	Median	Min	Max	Q1	Q3	Skewness	Kurtosis	N_outliers	N_outliers_low	N_outliers_high	Outlier_pct
Volume	caudate	L	control	96	-0.005	0.9272	-0.0239	-5.3691	2.6585	-0.433	0.4383	-1.574	13.661	4	1	3	4.2
Volume	caudate	L	preHD	98	-2.4422	1.4764	-2.3347	-7.4938	0.2274	-3.2385	-1.319	-0.869	4.189	3	3	0	3.1
Volume	caudate	L	earlyHD	92	-3.1182	2.6442	-3.1559	-9.0051	11.9041	-4.6663	-1.9265	1.937	12.864	3	1	2	3.3
Volume	caudate	R	control	96	-0.0507	0.8944	-0.0916	-2.5432	3.0486	-0.5313	0.4073	0.321	4.462	6	2	4	6.2
Volume	caudate	R	preHD	98	-2.3698	1.6302	-2.2852	-7.8763	3.8359	-3.3543	-1.2566	-0.313	5.776	4	3	1	4.1
Volume	caudate	R	earlyHD	92	-2.8828	2.2683	-2.9571	-8.0883	4.6247	-3.9298	-1.9441	0.576	5.247	9	5	4	9.8
Volume	putamen	L	control	96	-0.0023	0.7968	0.0559	-2.4818	2.4502	-0.5777	0.5271	0.021	3.715	2	1	1	2.1
Volume	putamen	L	preHD	98	-1.4405	1.1659	-1.2075	-5.1601	1.2376	-2.1672	-0.6771	-0.488	3.121	1	1	0	1
Volume	putamen	L	earlyHD	92	-1.3297	1.7196	-1.3442	-6.3277	6.7128	-2.1321	-0.3649	0.6	7.696	5	3	2	5.4
Volume	putamen	R	control	96	0.058	0.8117	-0.0094	-2.15	2.8568	-0.3862	0.5718	0.431	4.456	3	1	2	3.1
Volume	putamen	R	preHD	98	-1.5427	1.3087	-1.4339	-5.7763	3.3231	-2.2356	-0.899	-0.019	4.98	4	3	1	4.1
Volume	putamen	R	earlyHD	92	-1.3583	1.5529	-1.5384	-5.0226	6.2776	-2.2453	-0.4857	1.349	8.418	3	1	2	3.3
Curvature	caudate	L	control	96	0.0218	0.548	0.024	-1.9684	1.9953	-0.2502	0.2505	-0.022	6.245	6	2	4	6.2

Curvature	caudate	L	preHD	98	0.516	0.7756	0.5214	-2.4223	2.4443	0.0214	0.9872	-0.669	4.943	3	2	1	3.1
Curvature	caudate	L	earlyHD	92	0.5159	1.3325	0.6533	-3.8729	4.3124	-0.1496	1.3339	-0.347	4.421	5	3	2	5.4
Curvature	caudate	R	control	96	-0.016	0.4476	0.0482	-1.4673	1.5108	-0.2677	0.2346	-0.156	4.593	4	2	2	4.2
Curvature	caudate	R	preHD	98	0.5018	0.6824	0.5016	-1.1779	2.4762	0.0565	0.8316	0.14	3.216	2	1	1	2
Curvature	caudate	R	earlyHD	92	0.214	1.6365	0.3039	-6.1001	5.6807	-0.3839	0.9807	-0.428	6.731	8	5	3	8.7
Curvature	putamen	L	control	96	-0.0481	0.5711	-0.0195	-1.6483	2.3844	-0.3507	0.2275	0.427	6.055	7	4	3	7.3
Curvature	putamen	L	preHD	98	0.4879	0.6595	0.5219	-1.649	3.0869	0.1094	0.7962	0.458	6.04	6	2	4	6.1
Curvature	putamen	L	earlyHD	92	0.3712	0.779	0.3279	-1.906	2.7463	-0.1884	0.7762	0.25	4.202	4	2	2	4.3
Curvature	putamen	R	control	96	0.0424	0.5309	0.0054	-2.0314	1.7017	-0.2112	0.2678	-0.229	5.503	8	3	5	8.3
Curvature	putamen	R	preHD	98	0.4489	0.6422	0.3851	-1.5408	2.175	0.0845	0.7993	0.171	3.742	4	2	2	4.1
Curvature	putamen	R	earlyHD	92	0.4247	0.7759	0.3538	-1.6589	2.3124	-0.0913	0.933	-0.125	3.001	1	1	0	1.1
Flatness	caudate	L	control	96	0.0126	0.0904	0.0161	-0.197	0.4165	-0.0356	0.0621	0.759	6.33	4	2	2	4.2
Flatness	caudate	L	preHD	98	0.1072	0.1058	0.1211	-0.3247	0.3806	0.0597	0.1488	-0.619	6.175	10	4	6	10.2
Flatness	caudate	L	earlyHD	92	0.1031	0.1319	0.094	-0.1427	0.7667	0.0093	0.1642	1.543	8.657	3	0	3	3.3
Flatness	caudate	R	control	96	0.0041	0.089	0.0059	-0.2906	0.2192	-0.0418	0.0525	-0.316	4.002	5	3	2	5.2
Flatness	caudate	R	preHD	98	0.0945	0.1097	0.096	-0.4233	0.3375	0.0415	0.1581	-1.263	7.75	4	3	1	4.1
Flatness	caudate	R	earlyHD	92	0.1264	0.1093	0.134	-0.0875	0.6737	0.0558	0.181	1.287	8.546	2	0	2	2.2
Flatness	putamen	L	control	96	0.0241	0.1504	0.0105	-0.4033	0.6116	-0.0675	0.0897	0.766	5.281	5	1	4	5.2
Flatness	putamen	L	preHD	98	-0.0438	0.1609	-0.0389	-0.6004	0.3031	-0.118	0.0652	-0.762	4.862	3	3	0	3.1
Flatness	putamen	L	earlyHD	92	0.0594	0.2444	0.0129	-0.3273	1.7119	-0.0667	0.1617	3.381	23.945	1	0	1	1.1
Flatness	putamen	R	control	96	-0.0408	0.1722	-0.04	-0.8818	0.5487	-0.1039	0.0309	-0.799	9.098	8	4	4	8.3
Flatness	putamen	R	preHD	98	0.0023	0.1975	0.0042	-1.2026	0.4705	-0.0674	0.0735	-2.262	16.637	10	4	6	10.2
Flatness	putamen	R	earlyHD	92	0.065	0.2296	0.0638	-0.6402	1.0728	-0.0632	0.1722	0.798	7.252	4	1	3	4.3
Elongation	caudate	L	control	96	0.2034	1.0454	0.1441	-2.0133	6.9814	-0.2789	0.6637	2.732	19.975	5	3	2	5.2
Elongation	caudate	L	preHD	98	1.5755	1.2842	1.5632	-2.9348	4.9981	0.8877	2.3722	-0.268	4.111	3	2	1	3.1
Elongation	caudate	L	earlyHD	92	2.1566	2.5775	1.7125	-3.314	17.1713	0.5874	3.5569	2.143	13.889	1	0	1	1.1
Elongation	caudate	R	control	96	0.0889	0.9213	0.0711	-3.153	2.5652	-0.3542	0.6056	-0.267	4.423	5	2	3	5.2
Elongation	caudate	R	preHD	98	1.4064	1.3012	1.3129	-3.8131	4.2102	0.621	2.2608	-0.667	4.854	2	2	0	2
Elongation	caudate	R	earlyHD	92	2.5001	2.028	2.4371	-1.7988	12.5915	1.357	3.5154	1.205	8.301	1	0	1	1.1
Elongation	putamen	L	control	96	8.00E-04	0.6739	-0.0291	-1.3517	2.9769	-0.459	0.2602	1.16	6.28	4	0	4	4.2
Elongation	putamen	L	preHD	98	-0.2617	0.8034	-0.2316	-2.9929	1.6927	-0.648	0.2838	-0.766	4.784	4	3	1	4.1
Elongation	putamen	L	earlyHD	92	0.2788	1.4307	0.0311	-2.0151	9.7918	-0.475	0.8794	3.258	22.539	2	0	2	2.2
Elongation	putamen	R	control	96	-0.2931	0.706	-0.2609	-3.6005	1.9024	-0.5959	0.0477	-0.71	7.554	5	2	3	5.2
Elongation	putamen	R	preHD	98	-0.0268	0.8817	-0.0297	-4.4498	2.2266	-0.3562	0.2632	-1.467	10.682	11	4	7	11.2
Elongation	putamen	R	earlyHD	92	0.3367	1.4062	0.3123	-3.8181	7.0587	-0.3884	0.9507	1.145	8.503	4	1	3	4.3
Eccentricity	caudate	L	control	96	0.0063	0.0452	0.0081	-0.0986	0.2079	-0.0178	0.0311	0.752	6.307	4	2	2	4.2
Eccentricity	caudate	L	preHD	98	0.0535	0.0529	0.0606	-0.163	0.1898	0.0298	0.0742	-0.626	6.196	10	4	6	10.2
Eccentricity	caudate	L	earlyHD	92	0.0515	0.0661	0.0468	-0.0714	0.3844	0.0046	0.0821	1.551	8.703	3	0	3	3.3
Eccentricity	caudate	R	control	96	0.0021	0.0445	0.0031	-0.1451	0.1097	-0.0209	0.0263	-0.314	3.996	5	3	2	5.2
Eccentricity	caudate	R	preHD	98	0.0472	0.0548	0.048	-0.2123	0.1682	0.0208	0.0791	-1.272	7.785	4	3	1	4.1
Eccentricity	caudate	R	earlyHD	92	0.0632	0.0546	0.067	-0.043	0.3366	0.028	0.0907	1.286	8.531	2	0	2	2.2
Eccentricity	putamen	L	control	96	0.012	0.0752	0.0052	-0.2019	0.3053	-0.0338	0.0449	0.76	5.269	5	1	4	5.2
Eccentricity	putamen	L	preHD	98	-0.022	0.0806	-0.0195	-0.3015	0.1515	-0.0591	0.0327	-0.77	4.881	3	3	0	3.1

Eccentricity	putamen	L	earlyHD	92	0.0296	0.122	0.0066	-0.1638	0.8531	-0.0333	0.0811	3.36	23.776	1	0	1	1.1
Eccentricity	putamen	R	control	96	-0.0204	0.0862	-0.02	-0.4421	0.2739	-0.052	0.0155	-0.811	9.126	8	4	4	8.3
Eccentricity	putamen	R	preHD	98	0.001	0.099	0.002	-0.6052	0.2351	-0.0338	0.0367	-2.292	16.84	10	4	6	10.2
Eccentricity	putamen	R	earlyHD	92	0.0324	0.1148	0.0318	-0.3208	0.5359	-0.0316	0.0861	0.792	7.24	4	1	3	4.3
FADE Score	caudate	L	control	96	-0.1784	5.406	-0.1594	-15.4408	22.5066	-2.5951	3.0058	0.257	6.45	6	4	2	6.2
FADE Score	caudate	L	preHD	98	1.0294	7.1905	-0.1635	-13.3747	26.9725	-2.7764	3.2085	1.232	5.282	10	2	8	10.2
FADE Score	caudate	L	earlyHD	92	-1.3321	8.8956	0	-35.1611	27.9937	-6.2216	3.4571	-0.749	6.281	4	3	1	4.3
FADE Score	caudate	R	control	96	-0.3205	5.2158	-0.6339	-18.1863	16.189	-2.8975	2.251	-0.108	4.558	5	3	2	5.2
FADE Score	caudate	R	preHD	98	1.0745	6.4425	0.9291	-14.1126	34.4639	-2.8686	3.3926	1.662	9.895	4	1	3	4.1
FADE Score	caudate	R	earlyHD	92	-0.9639	9.2456	-1.3889	-32.3526	31.1001	-5.0292	4.6754	-0.219	5.444	3	2	1	3.3
FADE Score	putamen	L	control	96	0.2585	4.8766	0.0772	-14.8563	17.0171	-2.0847	3.1558	0.12	4.424	4	2	2	4.2
FADE Score	putamen	L	preHD	98	1.5608	6.4335	0.9718	-15.5499	26.5348	-1.6144	3.6431	0.684	5.664	8	3	5	8.2
FADE Score	putamen	L	earlyHD	92	0.8126	8.6771	1.1427	-29.0332	25.768	-4.306	5.5713	-0.216	4.701	4	2	2	4.3
FADE Score	putamen	R	control	96	0.2404	4.3852	0.3871	-14.5789	11.7738	-2.63	2.8103	-0.013	3.927	3	1	2	3.1
FADE Score	putamen	R	preHD	98	1.4396	5.9575	0.2929	-13.397	25.0815	-2.3764	3.6419	1.282	5.736	7	1	6	7.1
FADE Score	putamen	R	earlyHD	92	0.3202	7.3158	0.9176	-24.8323	21.1323	-3.7696	3.6887	-0.399	5.051	4	2	2	4.3

Supplementary File - Correlation Matrices

Cross-sectional (Baseline) correlation matrices

By region and participant group

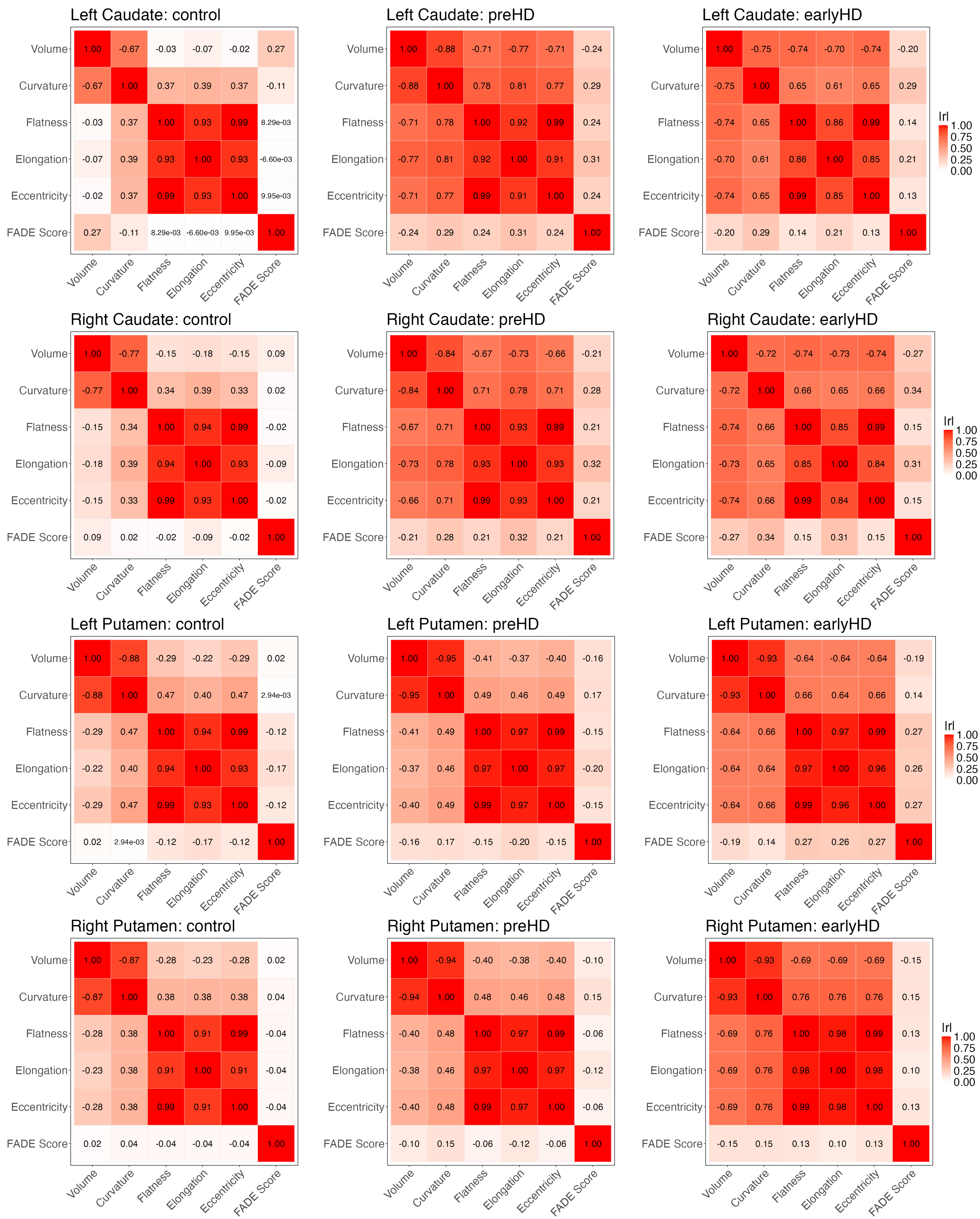

Longitudinal (annualized change) correlation matrices
By region and participant group

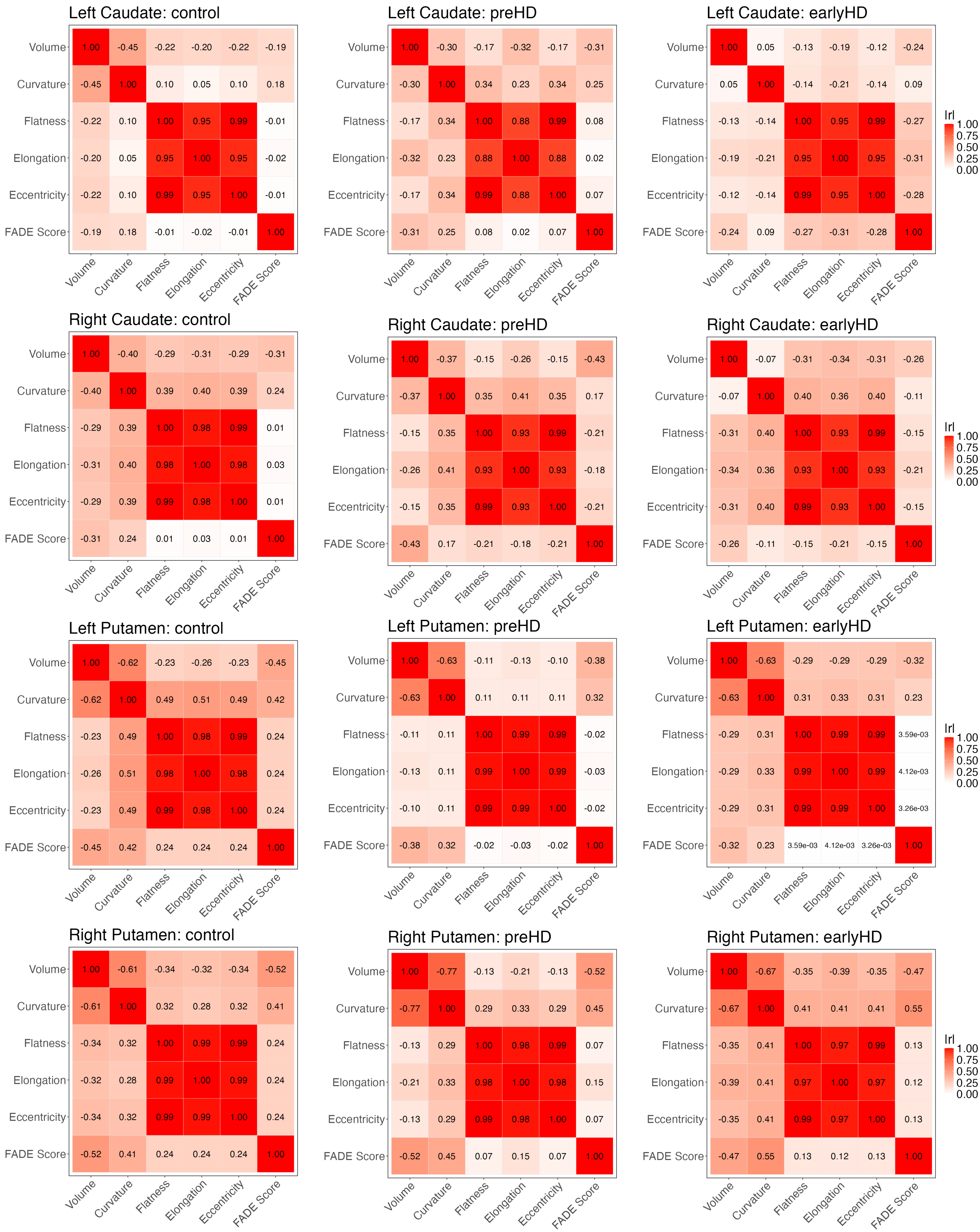

Supplementary File - Variance Partition

Tables of variance partition for Group, Age, Site, and Sex.

Variance partitioning was performed using the variancePartition R package (Hoffman & Schadt, 2016, BMC Bioinformatics). All predictors (i.e., group, age, site, and sex) were modeled as fixed effects. For each measure × region × hemisphere combination, the package fits a linear model and decomposes the total variance into proportions attributable to each predictor plus residuals. The five components (group, age, sex, site, and residuals) sum to 1 for each measure × region × hemisphere combination.

Cross-sect. Variance Explained							
Measure	Region	Hemisphere	Group	Age	Site	Sex	Residuals
Volume	caudate	L	60.2%	1.5%	1.3%	0.5%	36.4%
Curvature	caudate	L	54.7%	2.3%	0.5%	0.9%	41.6%
Flatness	caudate	L	51.1%	3.3%	1.7%	0.0%	43.9%
Elongation	caudate	L	51.4%	1.8%	0.9%	0.7%	45.2%
Eccentricity	caudate	L	50.9%	3.4%	1.8%	0.0%	44.0%
FADE Score	caudate	L	8.2%	0.0%	8.9%	1.6%	81.3%
Volume	caudate	R	60.7%	1.6%	1.2%	0.4%	36.1%
Curvature	caudate	R	53.3%	2.4%	4.1%	0.5%	39.7%
Flatness	caudate	R	51.7%	4.1%	0.7%	0.1%	43.3%
Elongation	caudate	R	52.9%	2.8%	0.1%	0.8%	43.3%
Eccentricity	caudate	R	51.6%	4.2%	0.8%	0.0%	43.4%
FADE Score	caudate	R	7.4%	0.0%	4.1%	1.3%	87.2%
Volume	putamen	L	64.3%	4.2%	0.4%	0.3%	30.8%
Curvature	putamen	L	60.4%	2.4%	0.5%	0.1%	36.6%
Flatness	putamen	L	31.0%	0.9%	2.1%	0.0%	66.0%
Elongation	putamen	L	34.4%	0.6%	1.4%	0.1%	63.6%
Eccentricity	putamen	L	30.7%	0.9%	2.1%	0.0%	66.2%
FADE Score	putamen	L	10.6%	0.1%	15.2%	0.0%	74.1%
Volume	putamen	R	64.3%	3.8%	0.6%	0.6%	30.8%
Curvature	putamen	R	62.1%	1.9%	0.3%	0.1%	35.6%
Flatness	putamen	R	39.1%	1.9%	5.8%	0.1%	53.0%
Elongation	putamen	R	41.2%	1.4%	4.1%	0.8%	52.7%
Eccentricity	putamen	R	38.9%	2.0%	5.8%	0.1%	53.2%
FADE Score	putamen	R	4.5%	0.2%	2.1%	0.0%	93.2%

See next page for longitudinal variance explained...

Longitudinal Variance Explained							
Measure	Region	Hemisphere	Group	Age	Site	Sex	Residuals
Volume	caudate	L	38.1%	2.2%	0.4%	0.4%	58.9%
Curvature	caudate	L	5.0%	0.1%	2.3%	0.4%	92.2%
Flatness	caudate	L	18.1%	0.4%	1.5%	0.6%	79.5%
Elongation	caudate	L	22.0%	0.4%	1.1%	0.2%	76.3%
Eccentricity	caudate	L	18.0%	0.4%	1.5%	0.6%	79.6%
FADE Score	caudate	L	2.3%	0.1%	0.2%	0.0%	97.4%
Volume	caudate	R	42.7%	2.2%	1.8%	0.5%	52.8%
Curvature	caudate	R	4.1%	0.7%	0.5%	0.3%	94.4%
Flatness	caudate	R	22.9%	0.5%	0.5%	0.5%	75.6%
Elongation	caudate	R	34.1%	0.4%	0.1%	0.1%	65.3%
Eccentricity	caudate	R	22.9%	0.5%	0.5%	0.5%	75.7%
FADE Score	caudate	R	1.3%	0.0%	2.1%	0.1%	96.5%
Volume	putamen	L	24.4%	2.1%	4.4%	1.2%	67.9%
Curvature	putamen	L	8.2%	0.7%	3.4%	0.1%	87.5%
Flatness	putamen	L	1.9%	0.0%	0.9%	0.2%	97.0%
Elongation	putamen	L	2.9%	0.0%	1.4%	0.1%	95.5%
Eccentricity	putamen	L	1.9%	0.0%	0.9%	0.2%	97.0%
FADE Score	putamen	L	0.6%	0.0%	6.3%	0.0%	93.0%
Volume	putamen	R	28.6%	0.6%	3.6%	0.0%	67.2%
Curvature	putamen	R	11.4%	0.3%	2.2%	0.6%	85.6%
Flatness	putamen	R	6.8%	0.9%	0.8%	0.6%	90.9%
Elongation	putamen	R	9.3%	1.0%	1.2%	0.6%	87.8%
Eccentricity	putamen	R	6.7%	0.9%	0.8%	0.6%	90.9%
FADE Score	putamen	R	1.5%	0.1%	2.9%	1.1%	94.5%

Supplementary File - ICV_correlations

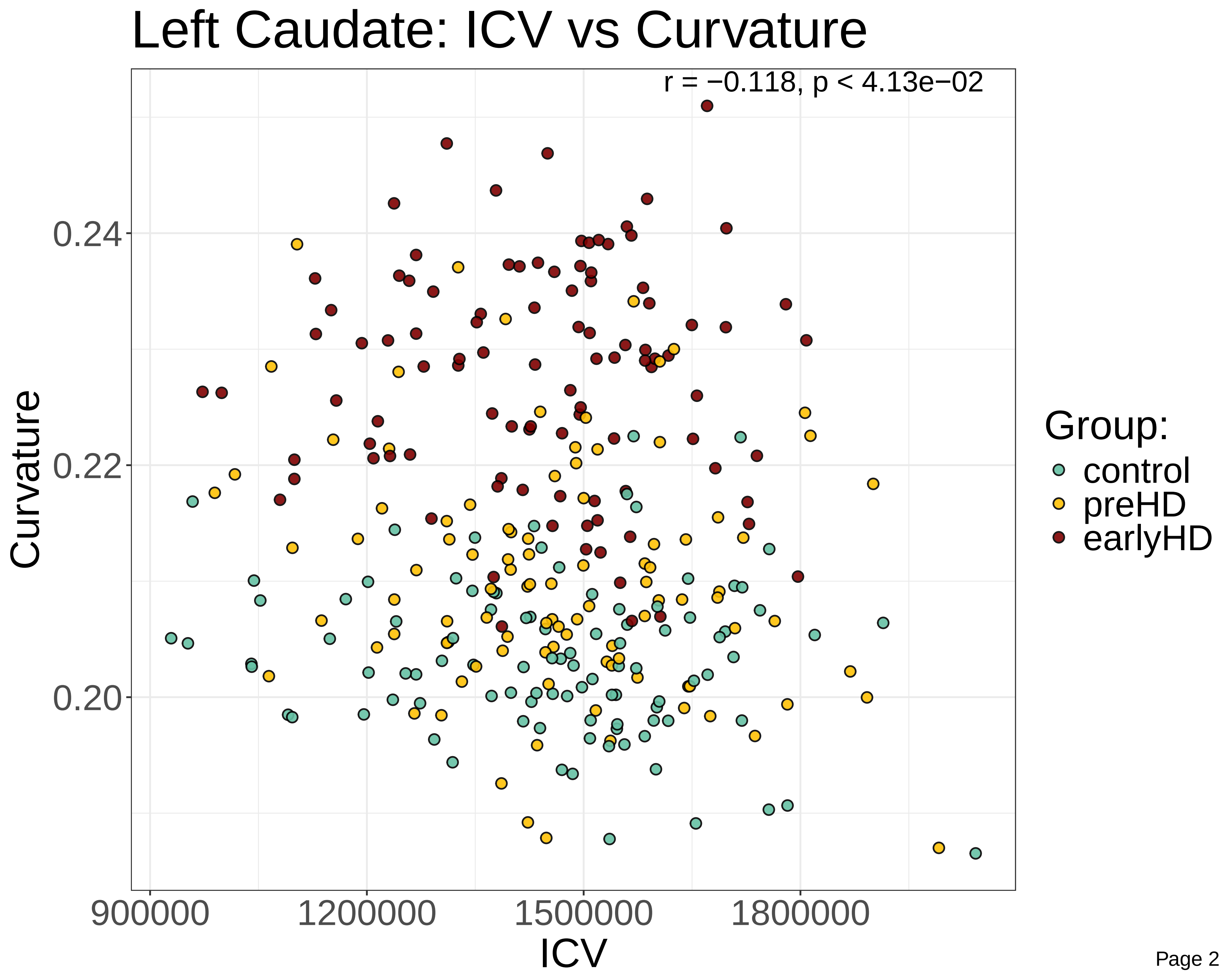
Left Caudate: ICV vs Curvature
r = −0.118, p < 4.13e−02
Curvature
0.24
0.22
0.20
900000
1200000
1500000
1800000
ICV
Group:
control
preHD
earlyHD

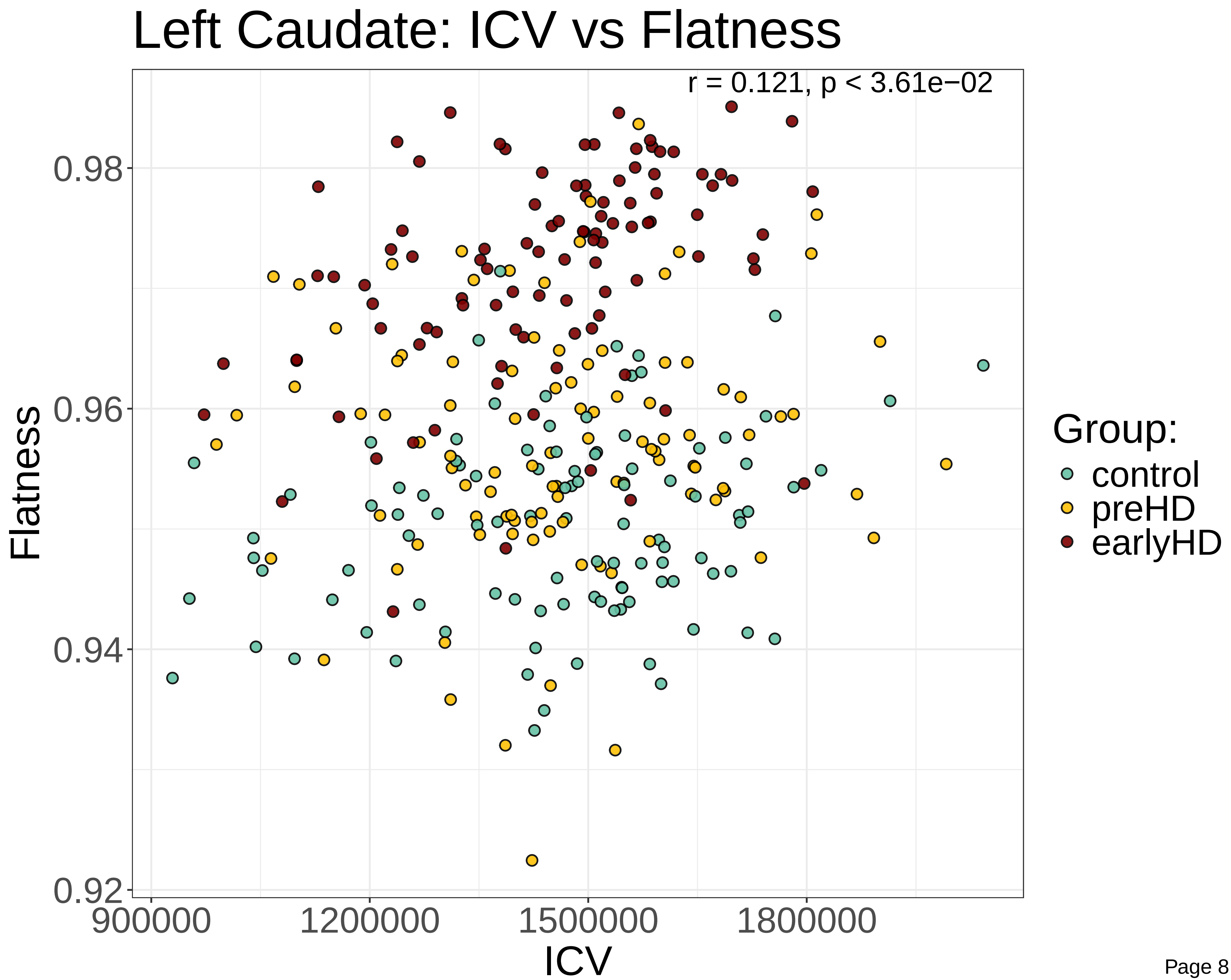
Left Caudate: ICV vs Flatness
r = 0.121, p < 3.61e−02
Flatness
0.98
0.96
0.94
0.92
ICV
900000
1200000
1500000
1800000
Group:
control
preHD
earlyHD

Left Caudate: ICV vs Elongation

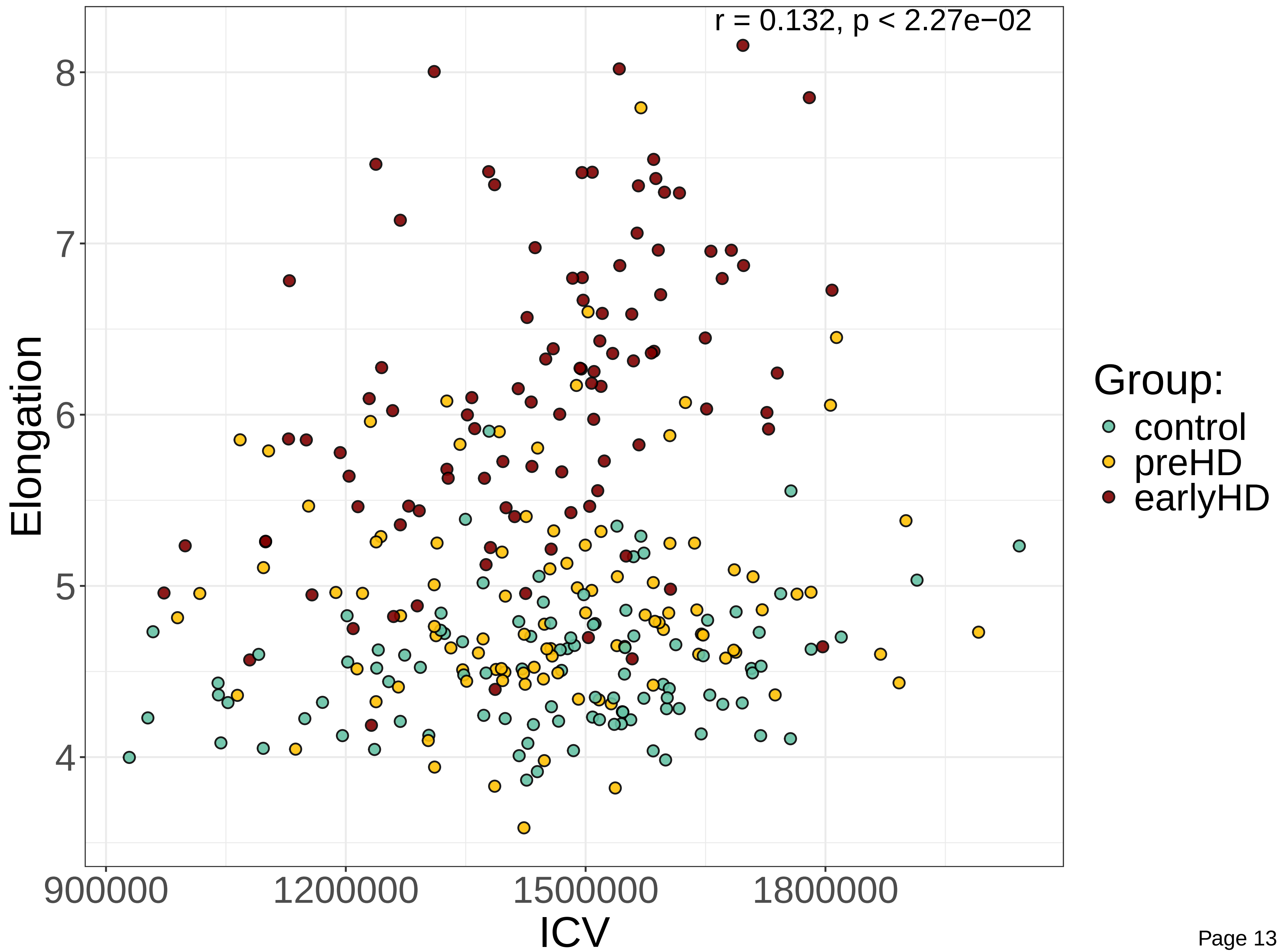

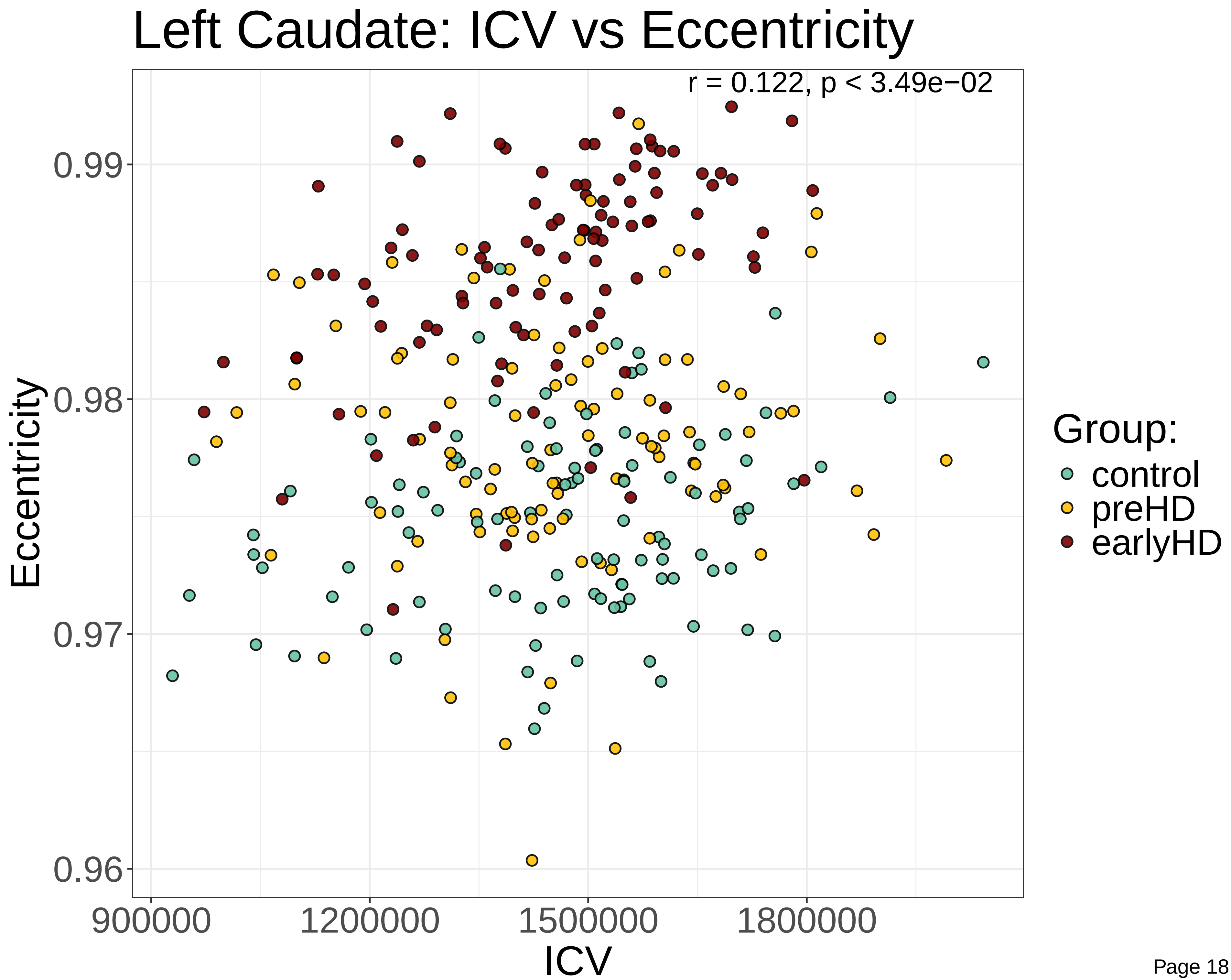
Left Caudate: ICV vs Eccentricity
r = 0.122, p < 3.49e−02
Eccentricity
0.99
0.98
0.97
0.96
ICV
900000
1200000
1500000
1800000
Group:
control
preHD
earlyHD

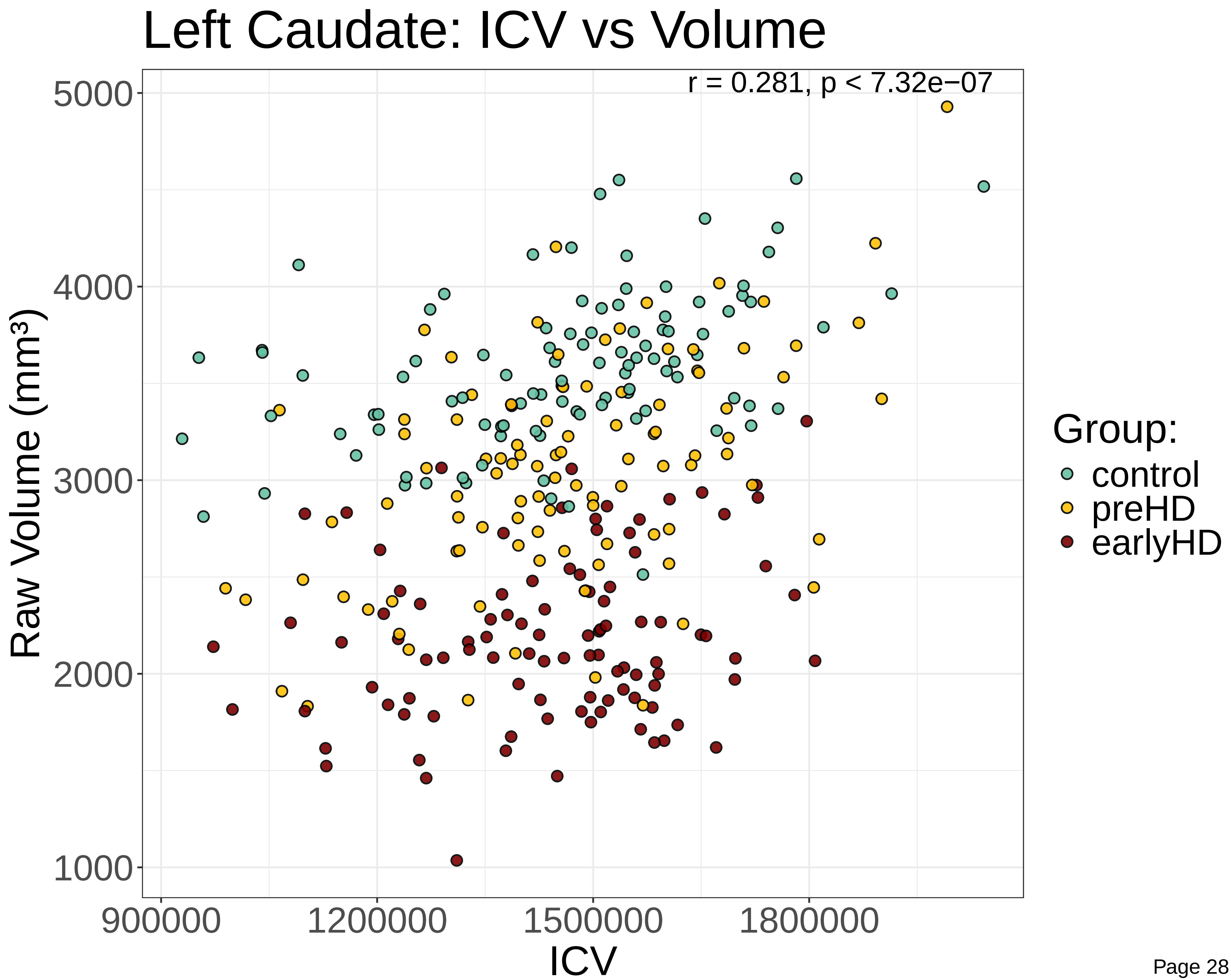

Left Caudate: ICV vs Volume
r = 0.281, p < 7.32e−07
Raw Volume (mm³)
5000
4000
3000
2000
1000
900000
1200000
1500000
1800000
ICV
Group:
control
preHD
earlyHD

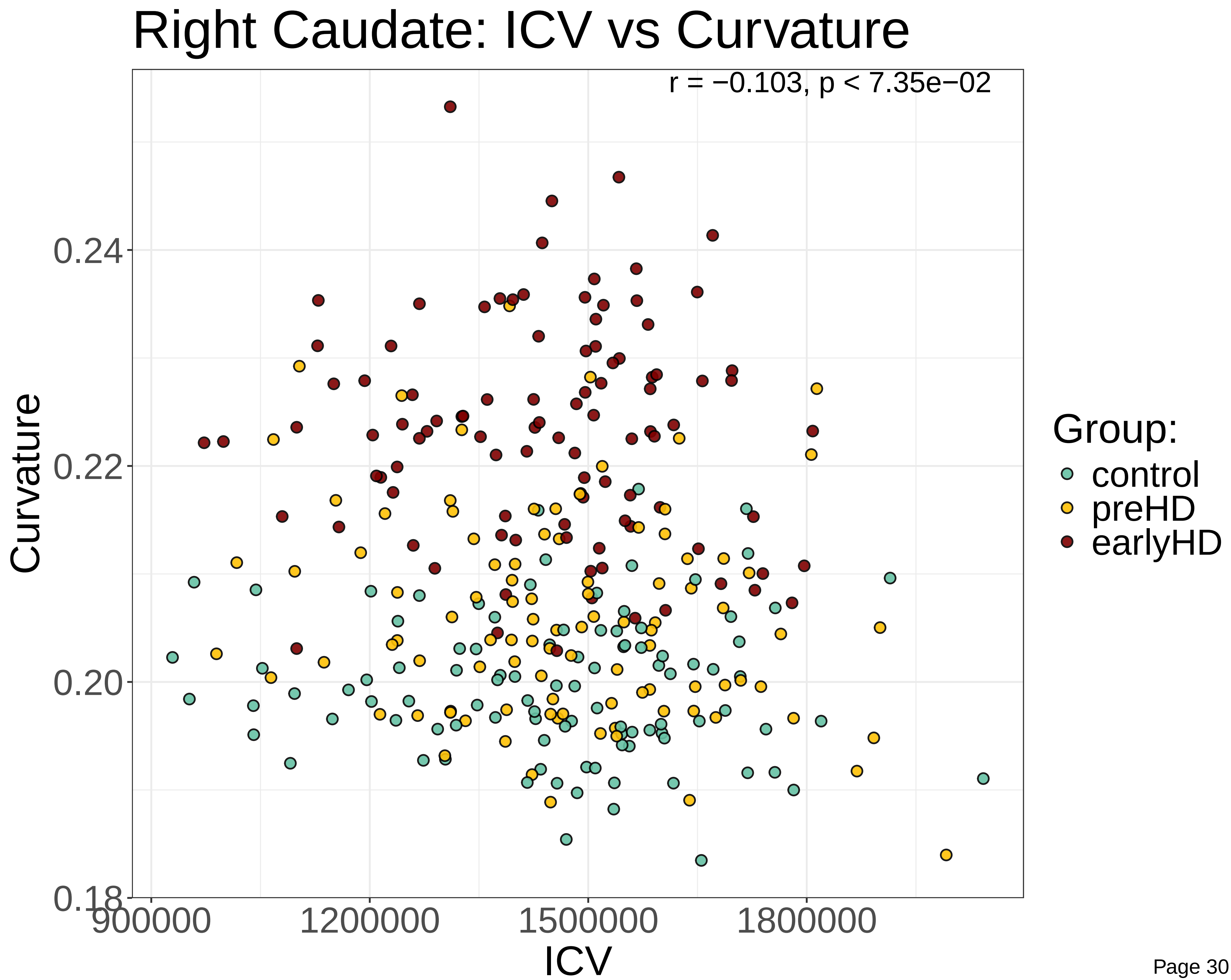
Right Caudate: ICV vs Curvature
r = −0.103, p < 7.35e−02
0.24
0.22
0.20
0.18
Curvature
900000
1200000
1500000
1800000
ICV
Group:
control
preHD
earlyHD

Right Caudate: ICV vs Flatness

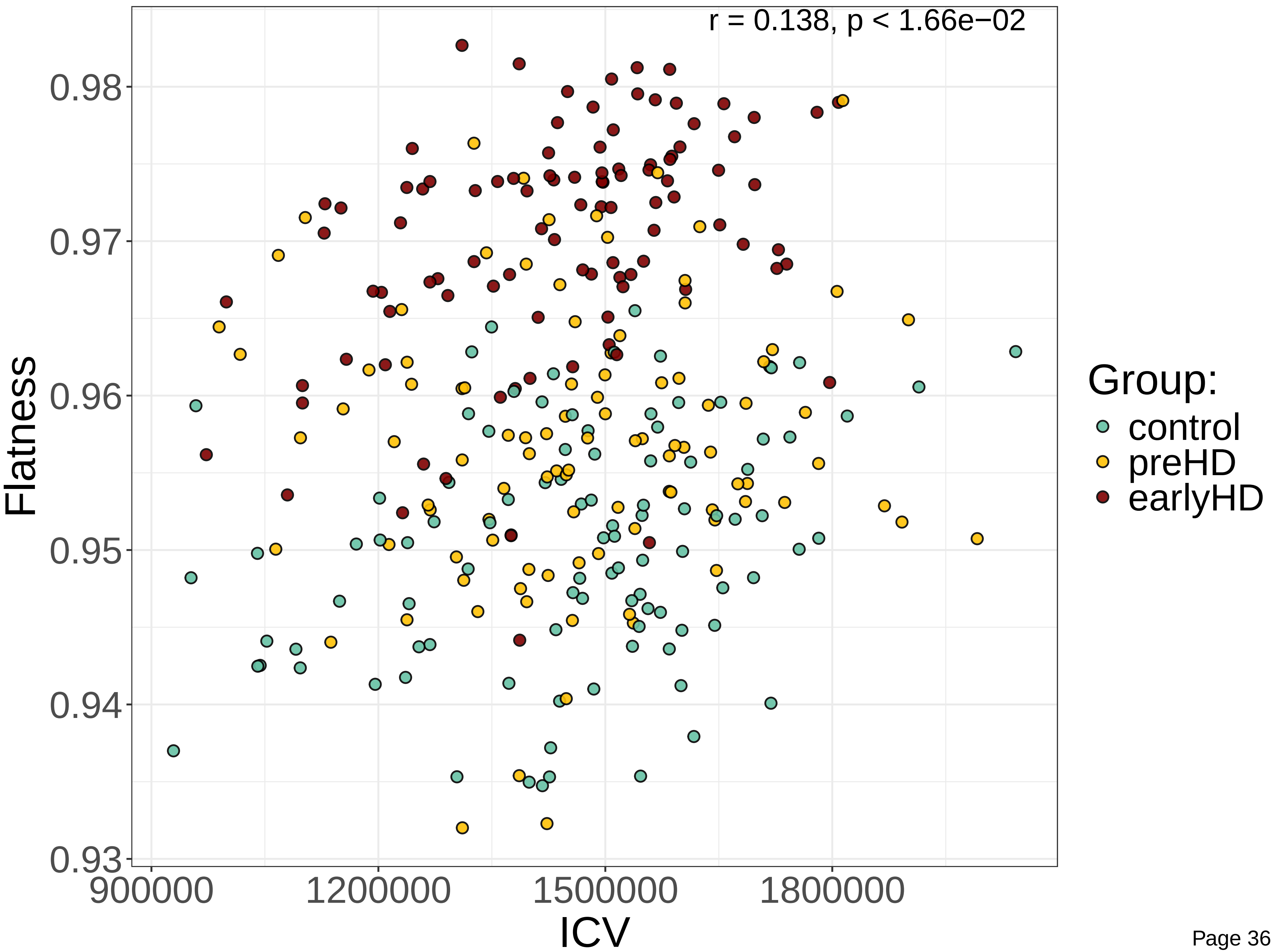

Right Caudate: ICV vs Elongation

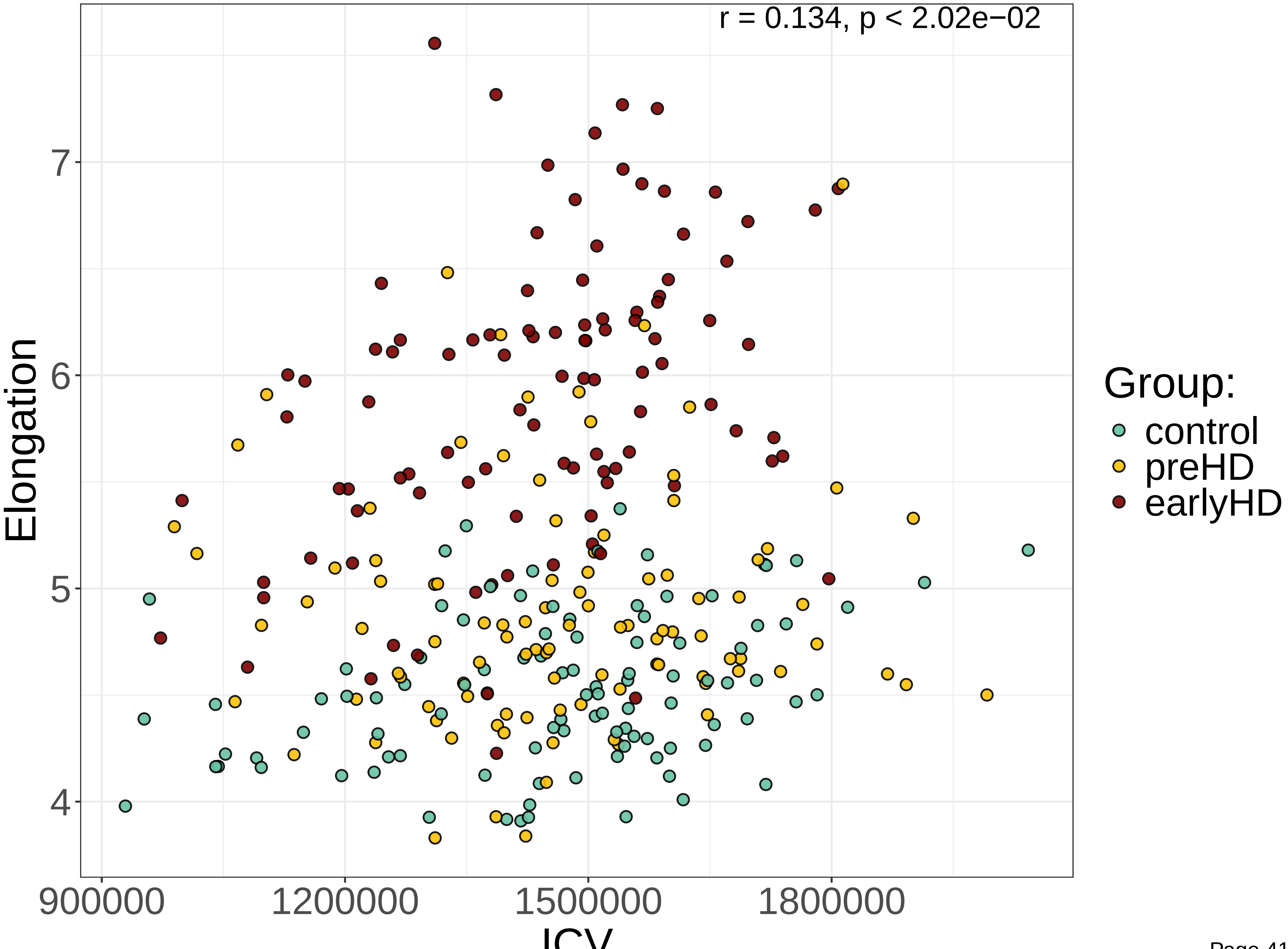

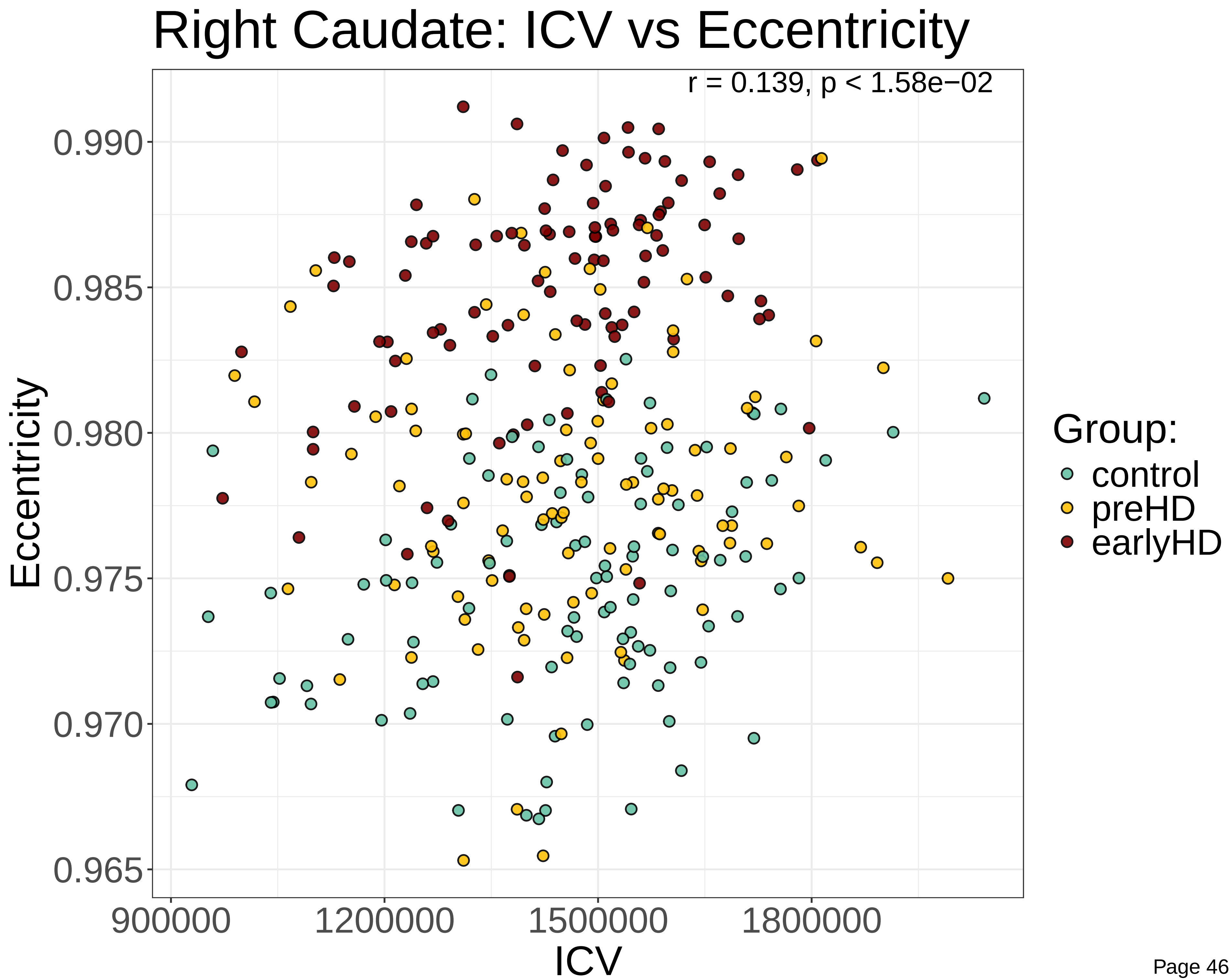
Right Caudate: ICV vs Eccentricity
r = 0.139, p < 1.58e−02
0.990
0.985
0.980
0.975
0.970
0.965
Eccentricity
900000
1200000
1500000
1800000
ICV
Group:
control
preHD
earlyHD

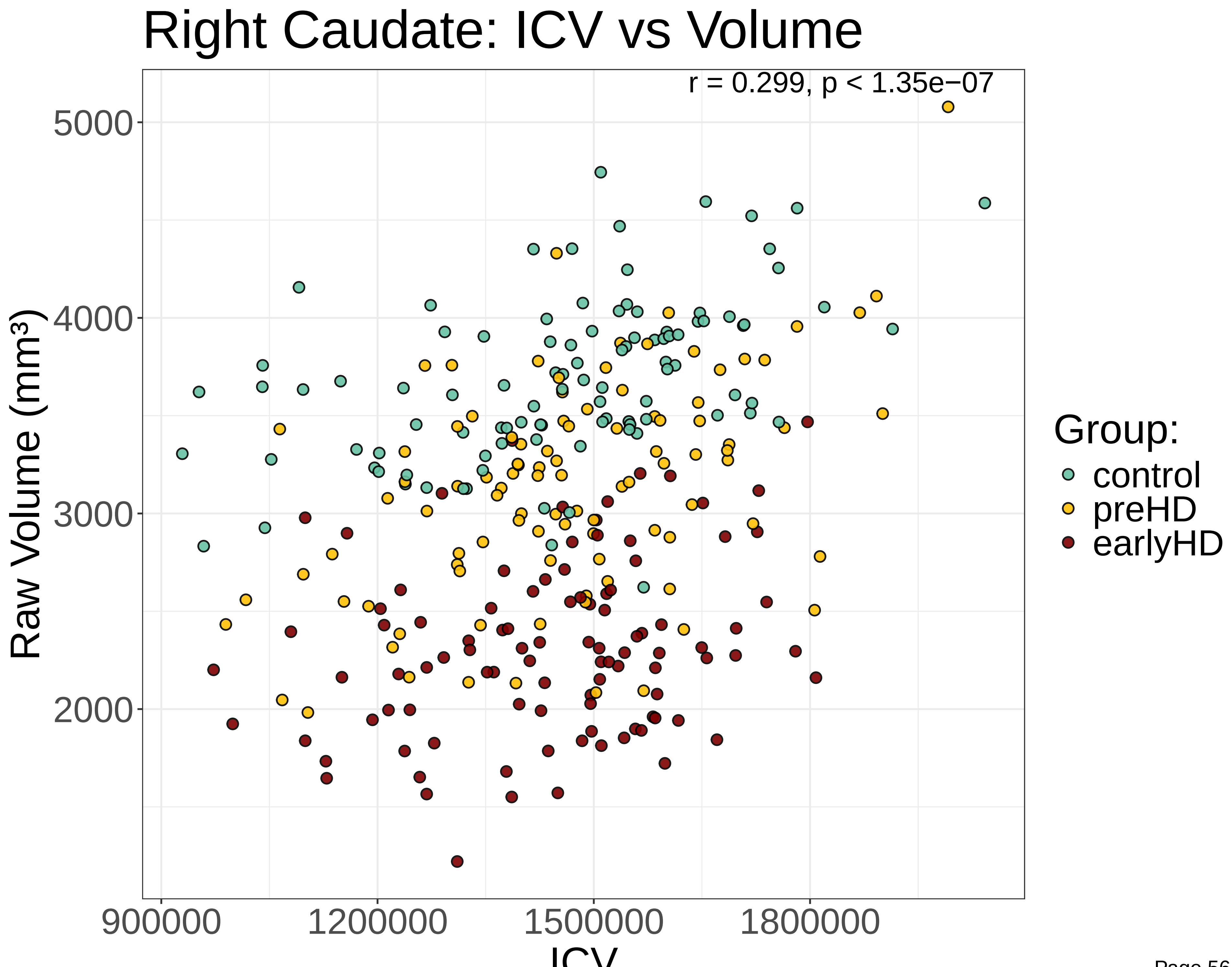
Right Caudate: ICV vs Volume
r = 0.299, p < 1.35e−07
Raw Volume (mm³)
5000
4000
3000
2000
900000
1200000
1500000
1800000
ICV
Group:
control
preHD
earlyHD

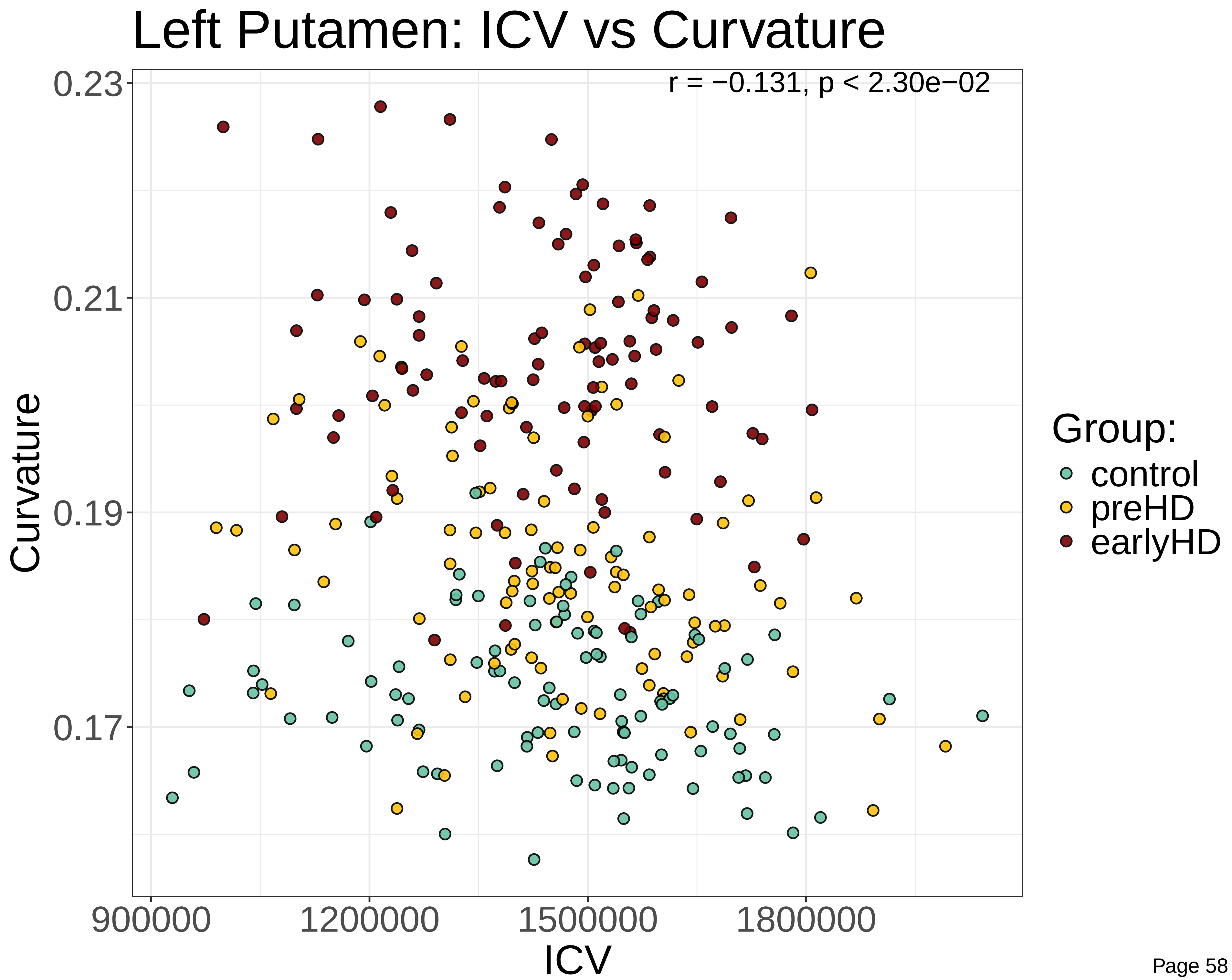
Left Putamen: ICV vs Curvature
r = −0.131, p < 2.30e−02
0.23
0.21
0.19
0.17
Curvature
900000
1200000
1500000
1800000
ICV
Group:
control
preHD
earlyHD

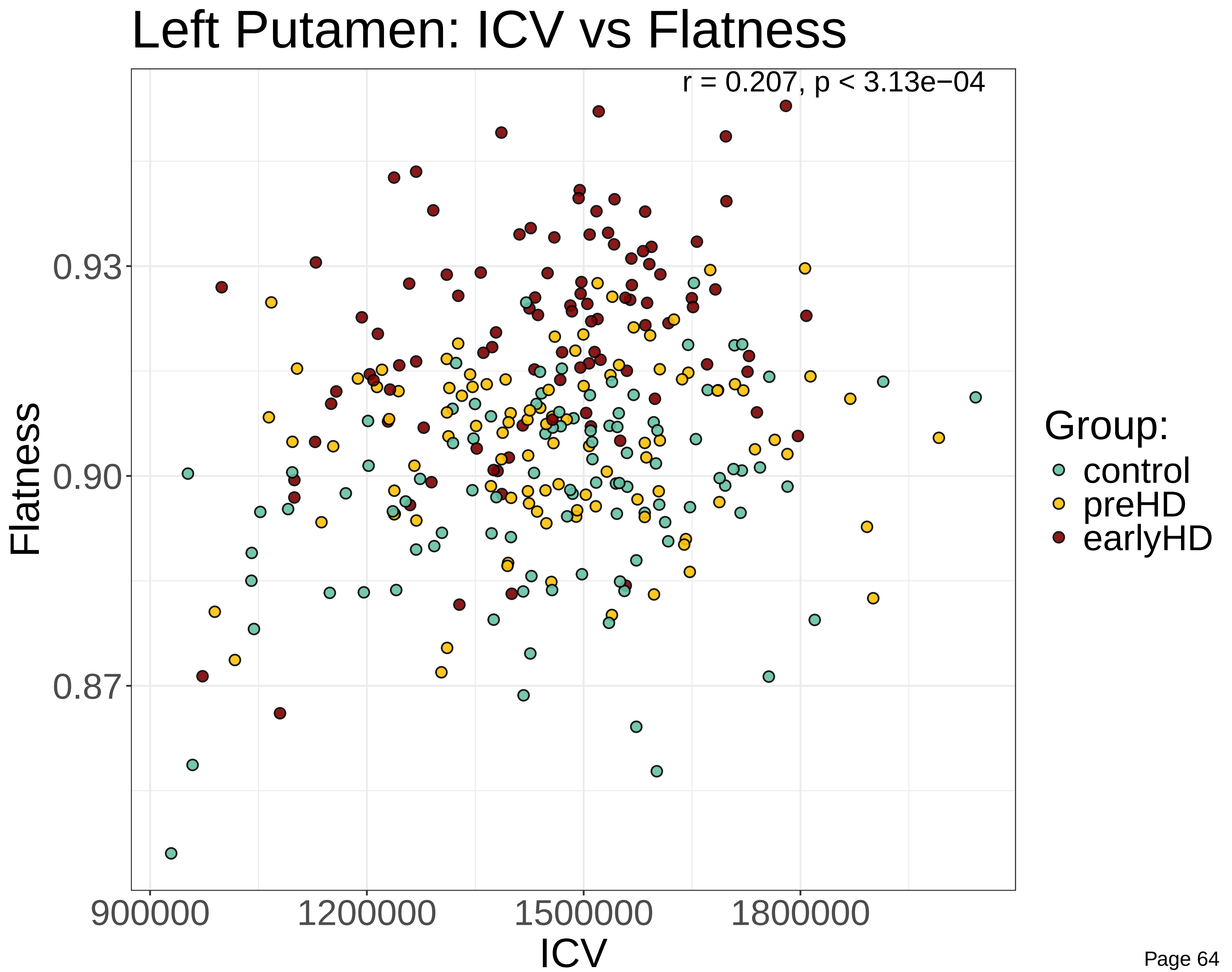
Left Putamen: ICV vs Flatness
r = 0.207, p < 3.13e−04
0.93
0.90
0.87
Flatness
900000
1200000
1500000
1800000
ICV
Group:
control
preHD
earlyHD

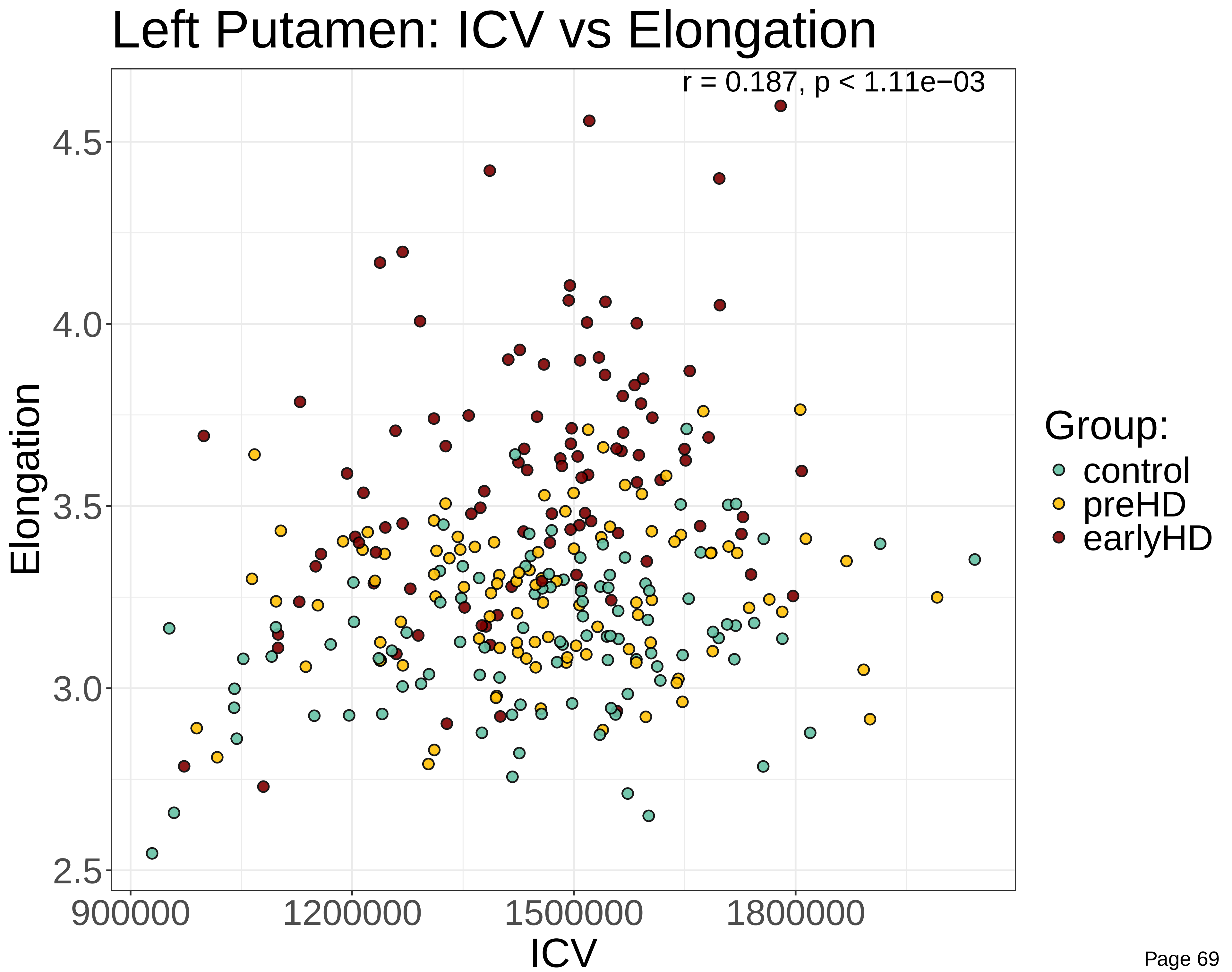
Left Putamen: ICV vs Elongation
r = 0.187, p < 1.11e−03
4.5
4.0
3.5
3.0
2.5
Elongation
900000
1200000
1500000
1800000
ICV
Group:
control
preHD
earlyHD

Left Putamen: ICV vs Eccentricity

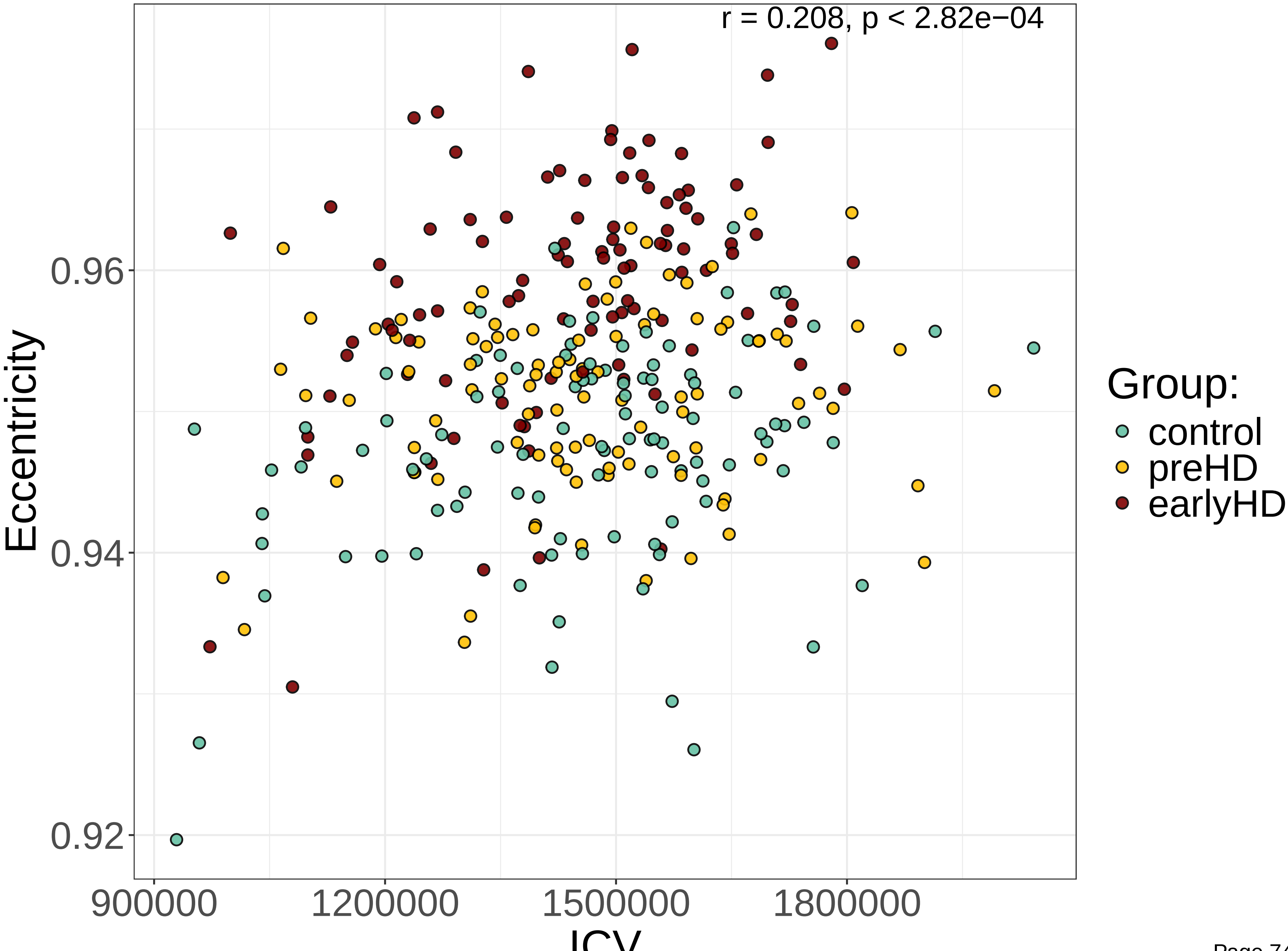

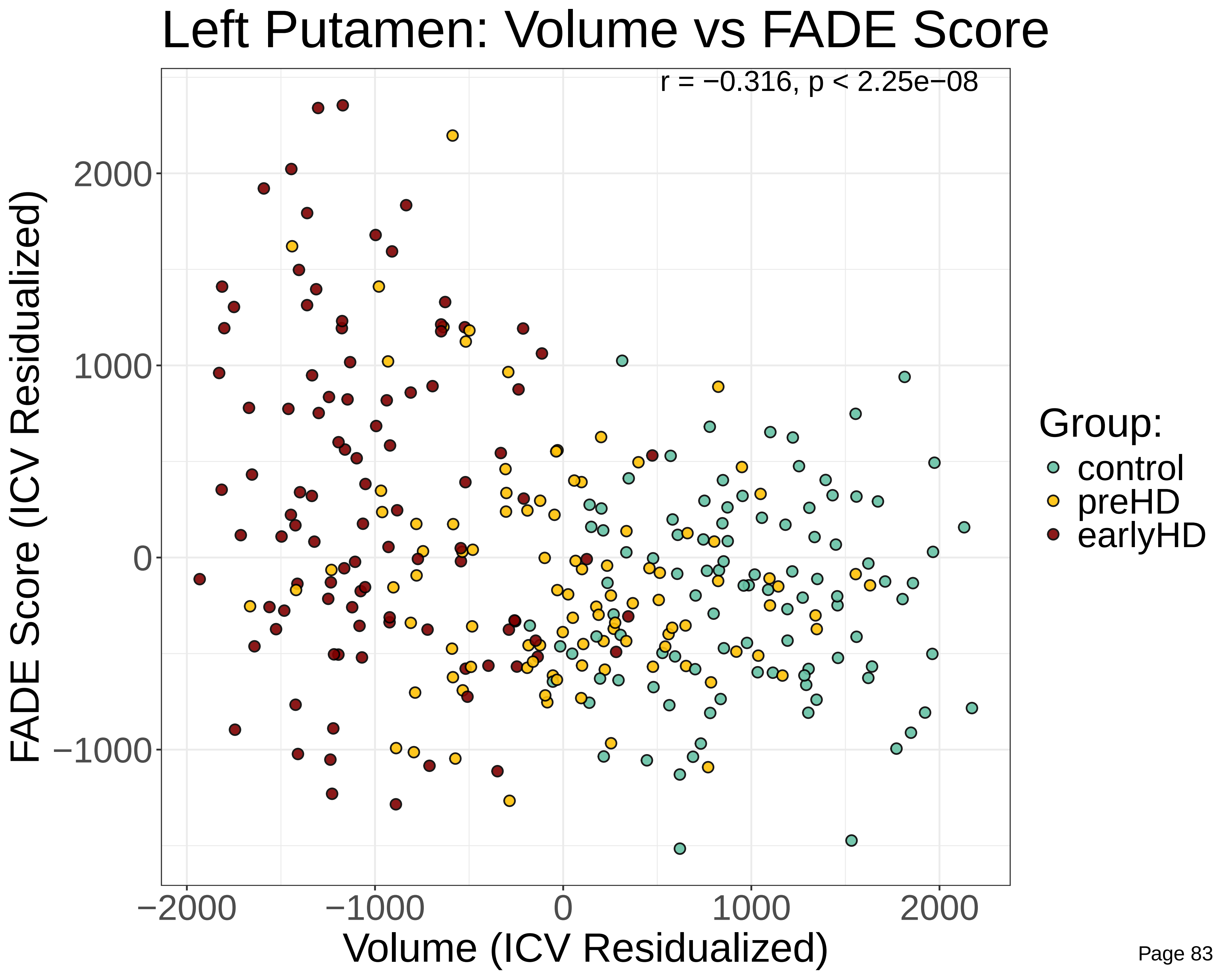

Left Putamen: Volume vs FADE Score
r = −0.316, p < 2.25e−08
FADE Score (ICV Residualized)
2000
1000
0
−1000
−2000
−1000
0
1000
2000
Volume (ICV Residualized)
Group:
control
preHD
earlyHD

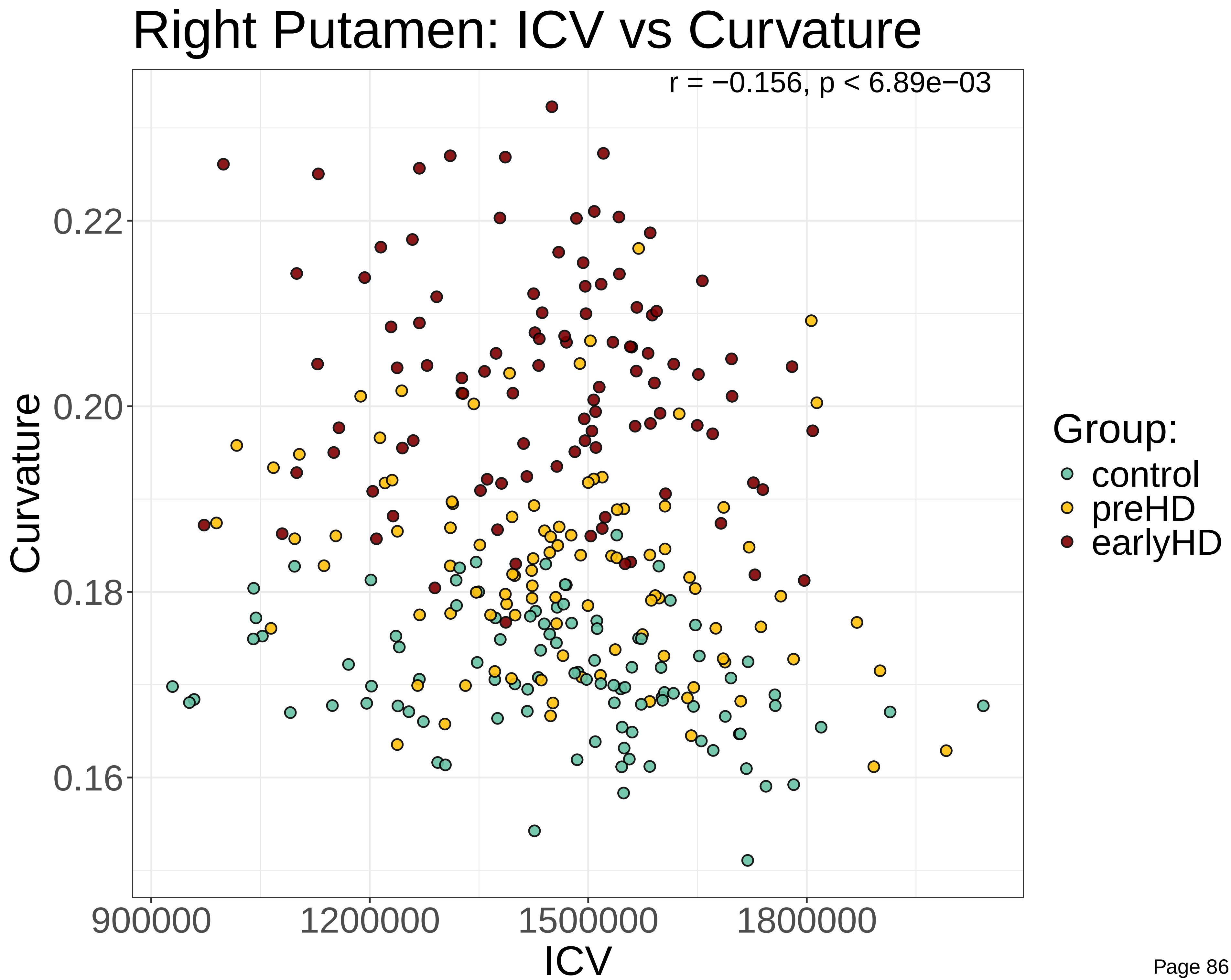

Right Putamen: ICV vs Curvature
r = −0.156, p < 6.89e−03
Curvature
0.22
0.20
0.18
0.16
900000
1200000
1500000
1800000
ICV
Group:
control
preHD
earlyHD

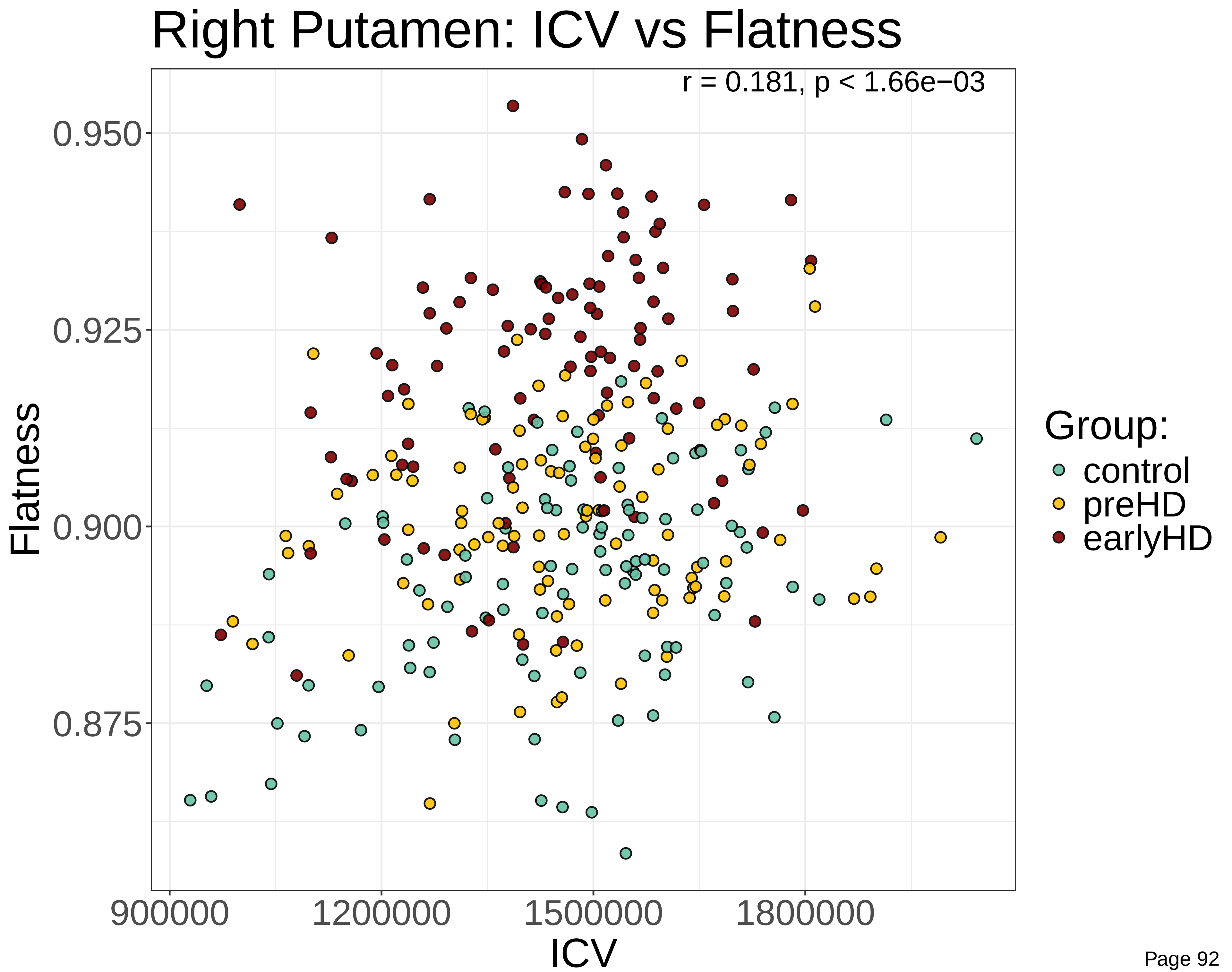
Right Putamen: ICV vs Flatness
r = 0.181, p < 1.66e−03
0.950
0.925
0.900
0.875
Flatness
900000
1200000
1500000
1800000
ICV
Group:
control
preHD
earlyHD

Right Putamen: ICV vs Elongation

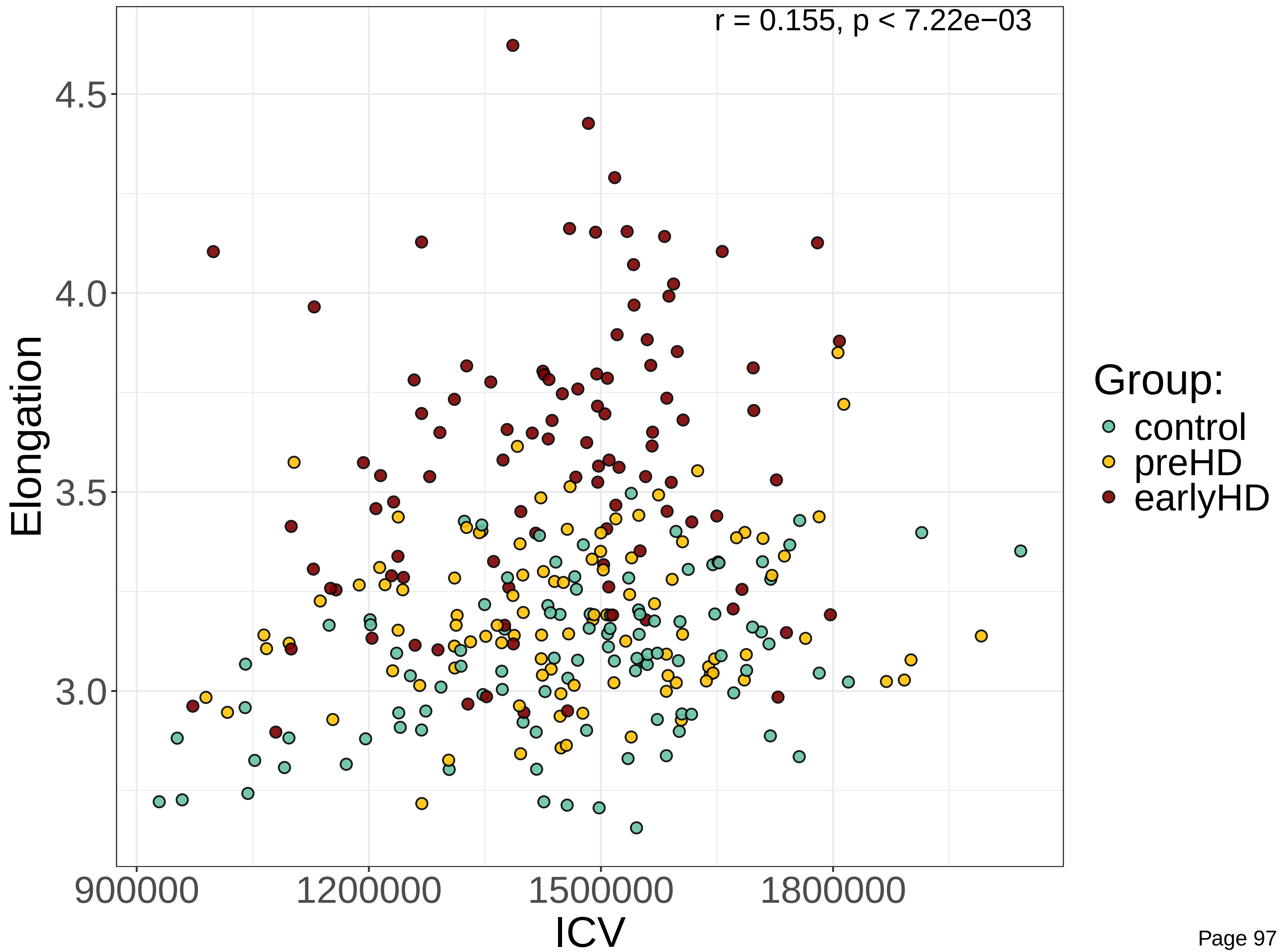

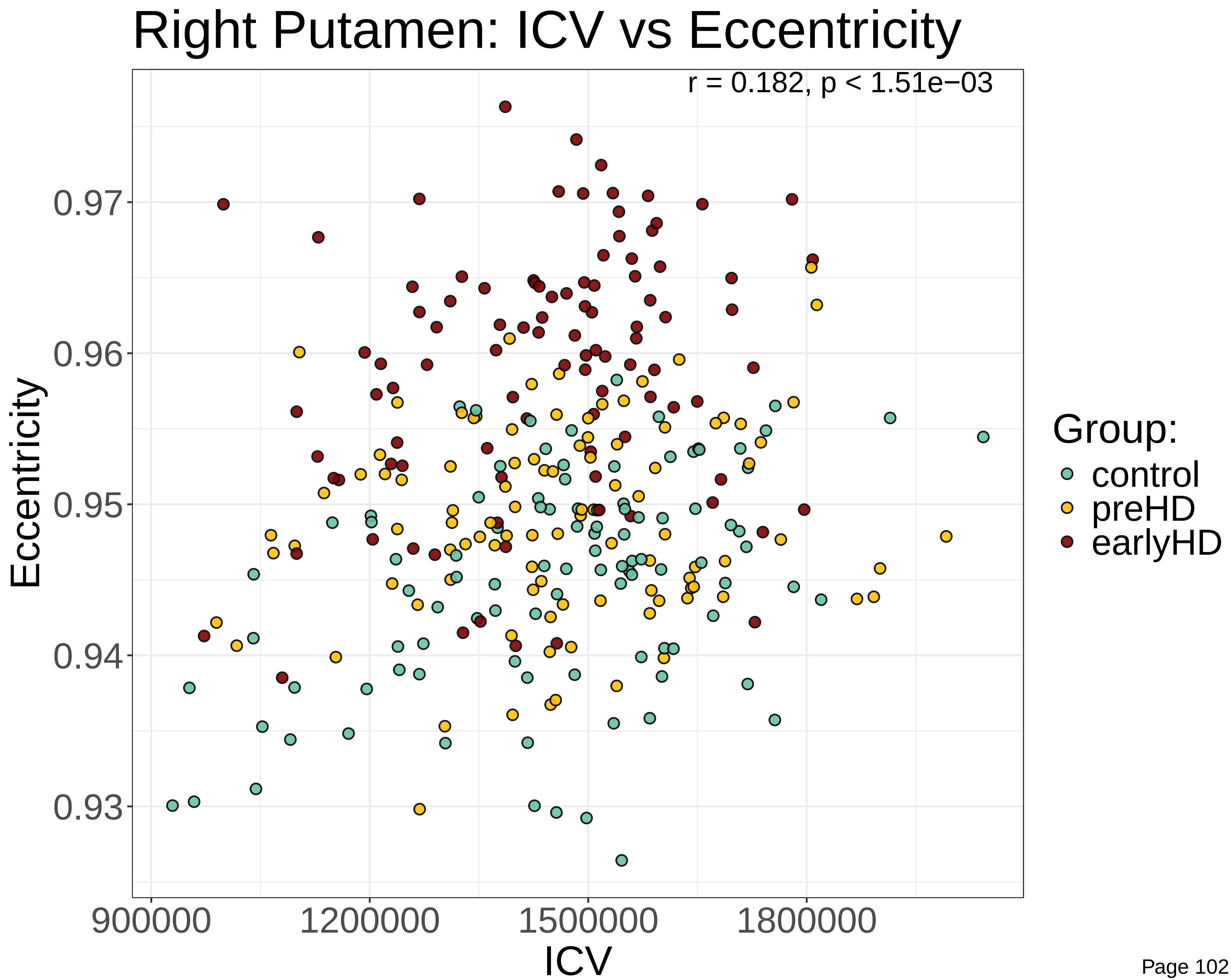
Right Putamen: ICV vs Eccentricity
r = 0.182, p < 1.51e−03
Eccentricity
0.97
0.96
0.95
0.94
0.93
900000
1200000
1500000
1800000
ICV
Group:
control
preHD
earlyHD

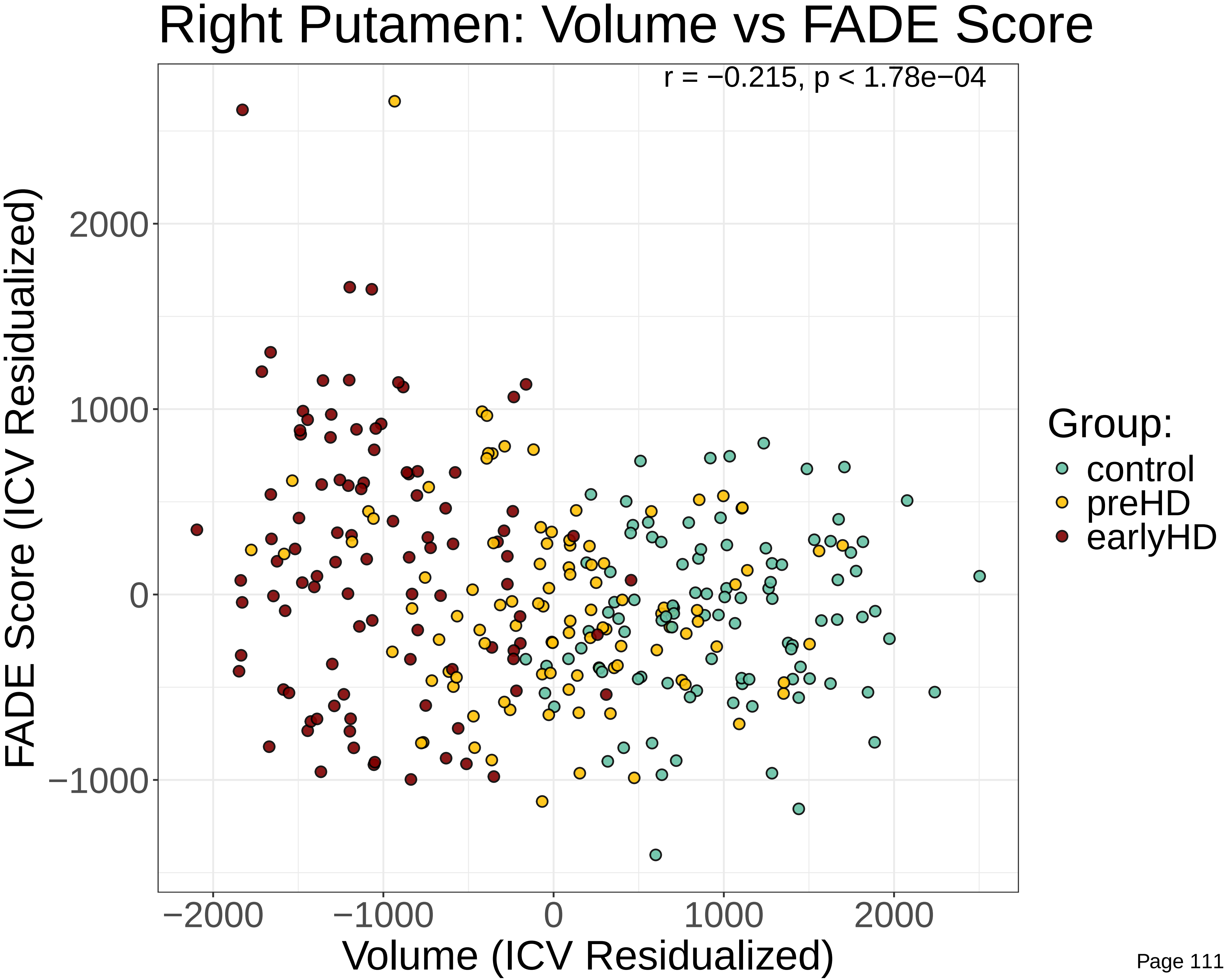
Right Putamen: Volume vs FADE Score
r = −0.215, p < 1.78e−04
FADE Score (ICV Residualized)
2000
1000
0
−1000
−2000
−1000
0
1000
2000
Volume (ICV Residualized)
Group:
control
preHD
earlyHD

Supplementary File - RH Violin Plots

Right Hemisphere Caudate and Putamen: **Cross Sectional**

*Violin plots with overlaid boxplots. Scores are adjusted for age and intracranial volume (ICV). Horizontal bars above the plots show the effect size marked by asterisks for Wilcoxon tests with * p < 0.05, ** p < 0.01, and *** p < 0.001 (uncorrected, see manuscript for FDR corrected table).*

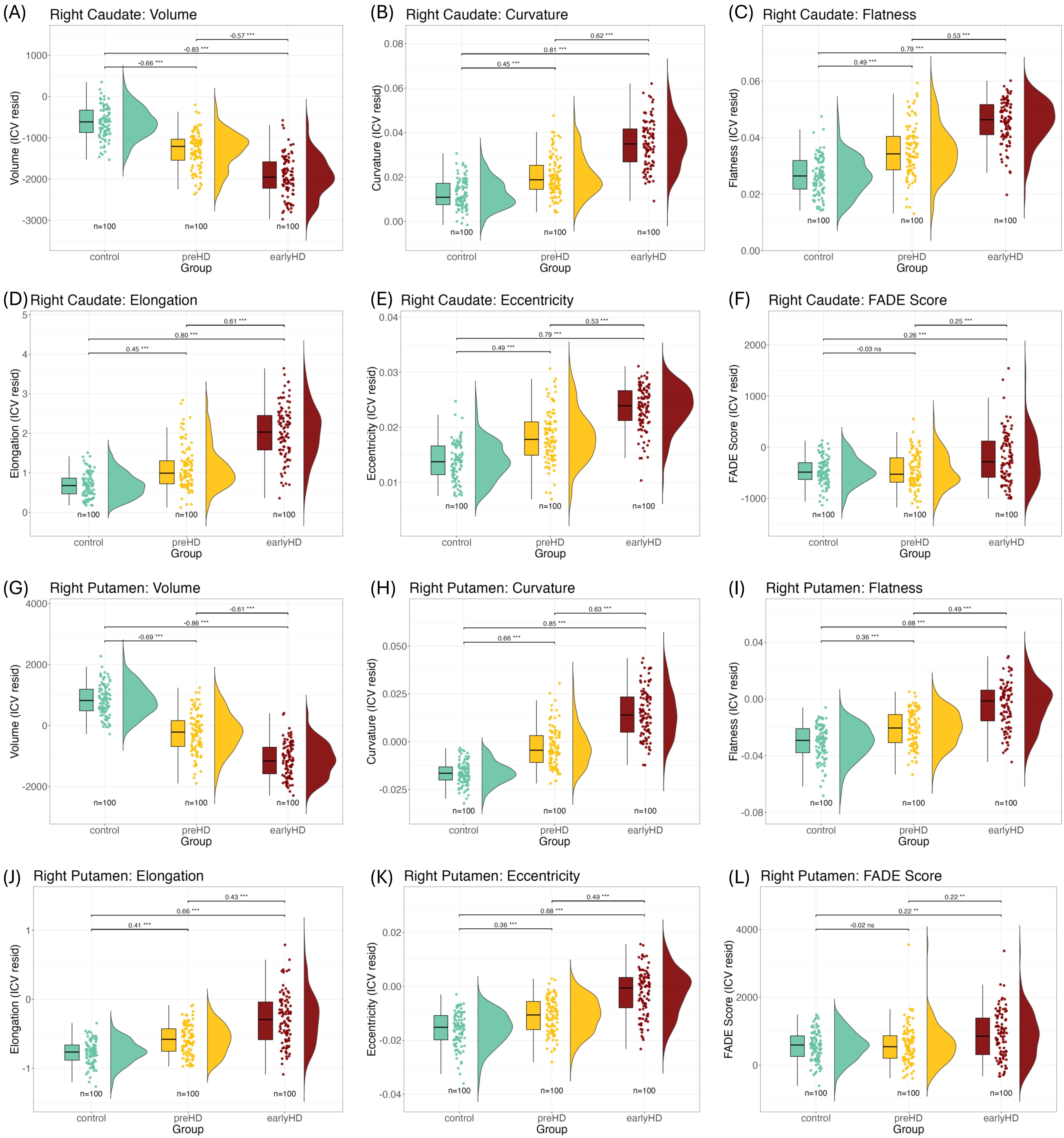

Right Hemisphere Caudate and Putamen: ***Longitudinal***

*Violin plots with overlaid boxplots. Scores are adjusted for age and intracranial volume (ICV). Horizontal bars above the plots show the effect size marked by asterisks when Wilcoxon tests had * p < 0.05, ** p < 0.01, and *** p < 0.001 (uncorrected, see manuscript for FDR corrected table).*

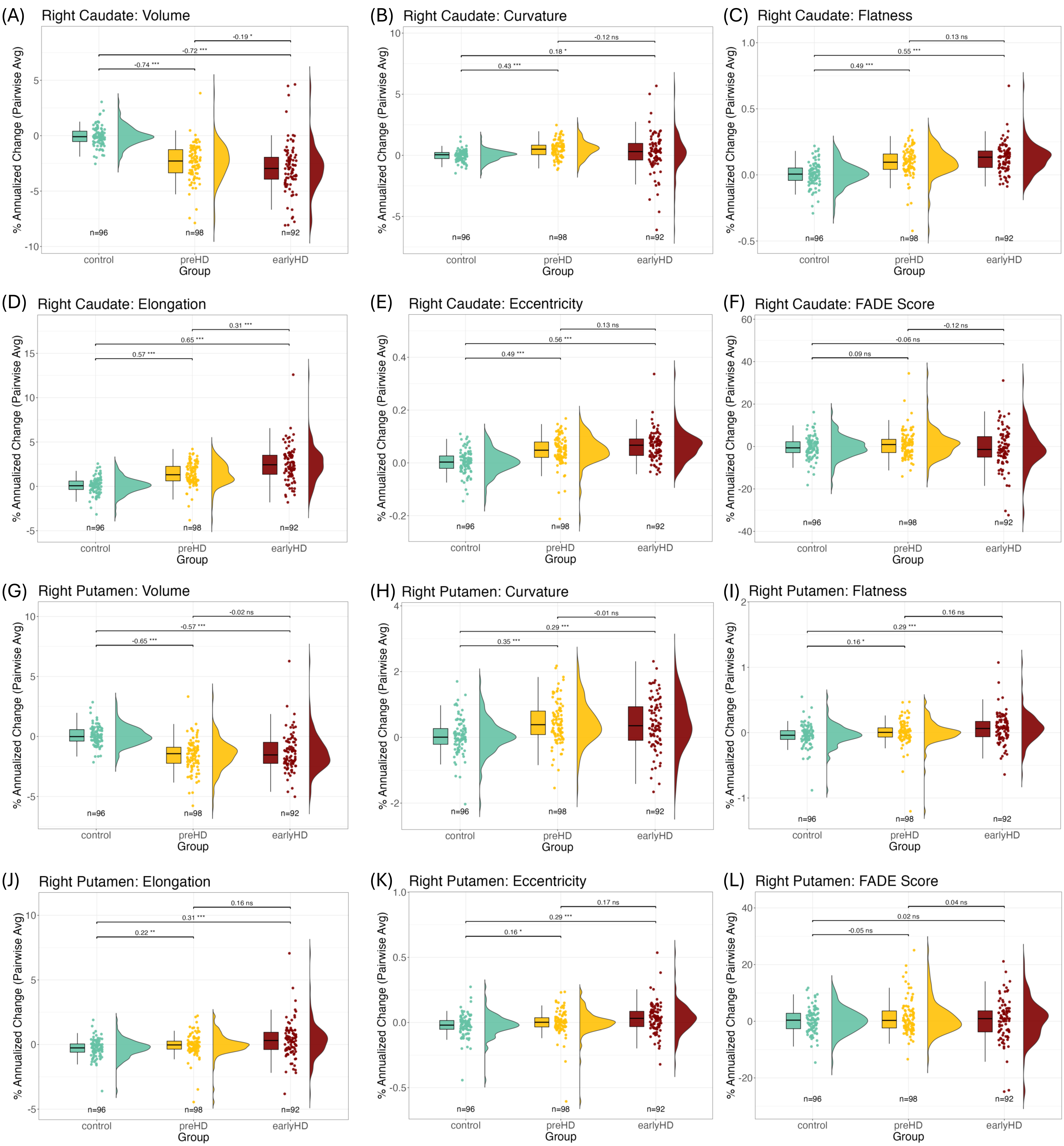

Supplementary File - Caudate vs. CaudVentWall

↓

Tables of Rank Biserial effect sizes for the whole caudate and caudate-ventricle wall. Color bars are added for easy visualization. The "Difference" columns show the change in effect size when moving from whole caudate to caudate-ventricle wall, and are color coded to expose gain (blue) or loss (yellow). Color bars in the "Difference" column fill the cell at +/- 0.3. Values of "volume" and "FADE" are not applicable for caudate-ventricle wall.

Cross-sectional		Whole Caudate		Caud-Vent Wall		Difference	
		Left	*Right*	*Left*	*Right*	*Left*	*Right*
**HC vs. earlyHD**	Volume	0.83	0.83				
	Curvature	0.82	0.81	0.66	0.60	-0.16	-0.21
	Flatness	0.78	0.79	0.76	0.75	-0.02	-0.04
	Elongation	0.79	0.80	0.77	0.75	-0.02	-0.05
	Eccentricity	0.78	0.79	0.76	0.75	-0.02	-0.04
	FADE	0.27	0.26				
**HC vs. preHD**	Volume	0.64	0.66				
	Curvature	0.47	0.45	0.28	0.19	-0.19	-0.26
	Flatness	0.46	0.49	0.49	0.50	0.03	0.01
	Elongation	0.45	0.45	0.49	0.49	0.04	0.04
	Eccentricity	0.46	0.49	0.49	0.50	0.03	0.01
	FADE	0.01	0.03				
**preHD vs. earlyHD**	Volume	0.58	0.57				
	Curvature	0.62	0.62	0.46	0.45	-0.16	-0.17
	Flatness	0.54	0.53	0.50	0.42	-0.04	-0.11
	Elongation	0.61	0.61	0.52	0.44	-0.09	-0.17
	Eccentricity	0.54	0.53	0.49	0.42	-0.05	-0.11
	FADE	0.28	0.25				

Longitudinal		Whole Caudate		Caud-Vent Wall		Difference	
		Left	*Right*	*Left*	*Right*	*Left*	*Right*
**HC vs. earlyHD**	Volume	0.72	0.72				
	Curvature	0.32	0.18	0.13	0.03	-0.19	-0.15
	Flatness	0.39	0.55	0.22	0.41	-0.17	-0.14
	Elongation	0.53	0.65	0.32	0.46	-0.21	-0.19
	Eccentricity	0.38	0.56	0.22	0.41	-0.16	-0.15
	FADE	0.04	0.06				
**HC vs. preHD**	Volume	0.78	0.74				
	Curvature	0.40	0.43	0.16	0.14	-0.24	-0.29
	Flatness	0.51	0.49	0.31	0.40	-0.20	-0.09
	Elongation	0.59	0.57	0.39	0.45	-0.20	-0.12
	Eccentricity	0.51	0.49	0.31	0.40	-0.20	-0.09
	FADE	0.03	0.09				
**preHD vs. earlyHD**	Volume	0.23	0.19				
	Curvature	0.02	0.12	0.01	0.05	-0.01	-0.07
	Flatness	0.08	0.13	0.05	0.10	-0.03	-0.03
	Elongation	0.09	0.31	0.07	0.19	-0.02	-0.12
	Eccentricity	0.08	0.13	0.05	0.10	-0.03	-0.03
	FADE	0.08	0.12				

Supplementary File - Spaghetti Plots by Age

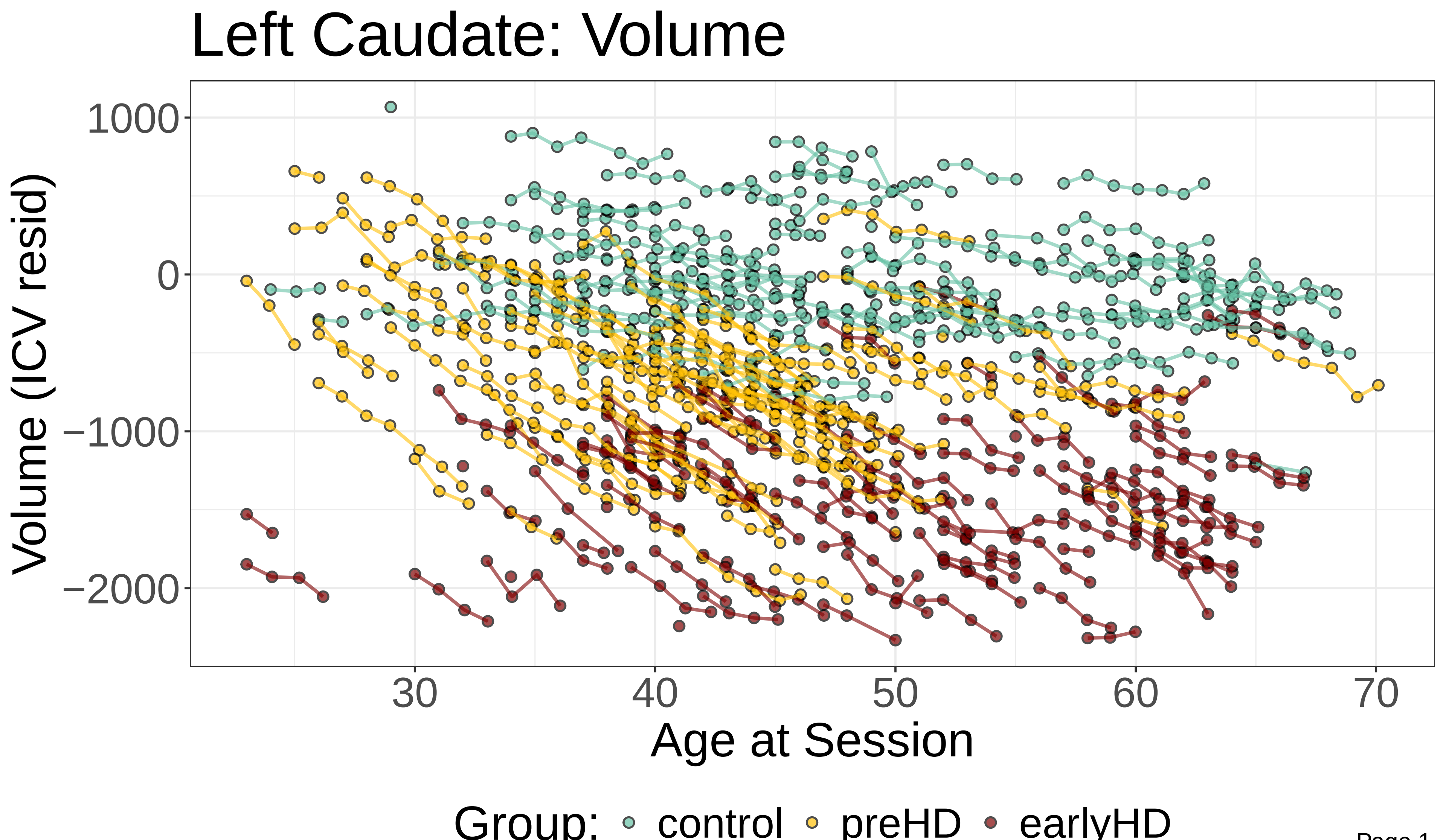

Left Caudate: Volume
1000
0
−1000
−2000
30
40
50
60
70
Volume (ICV resid)
Age at Session
Group: control preHD earlyHD

Right Caudate: Volume

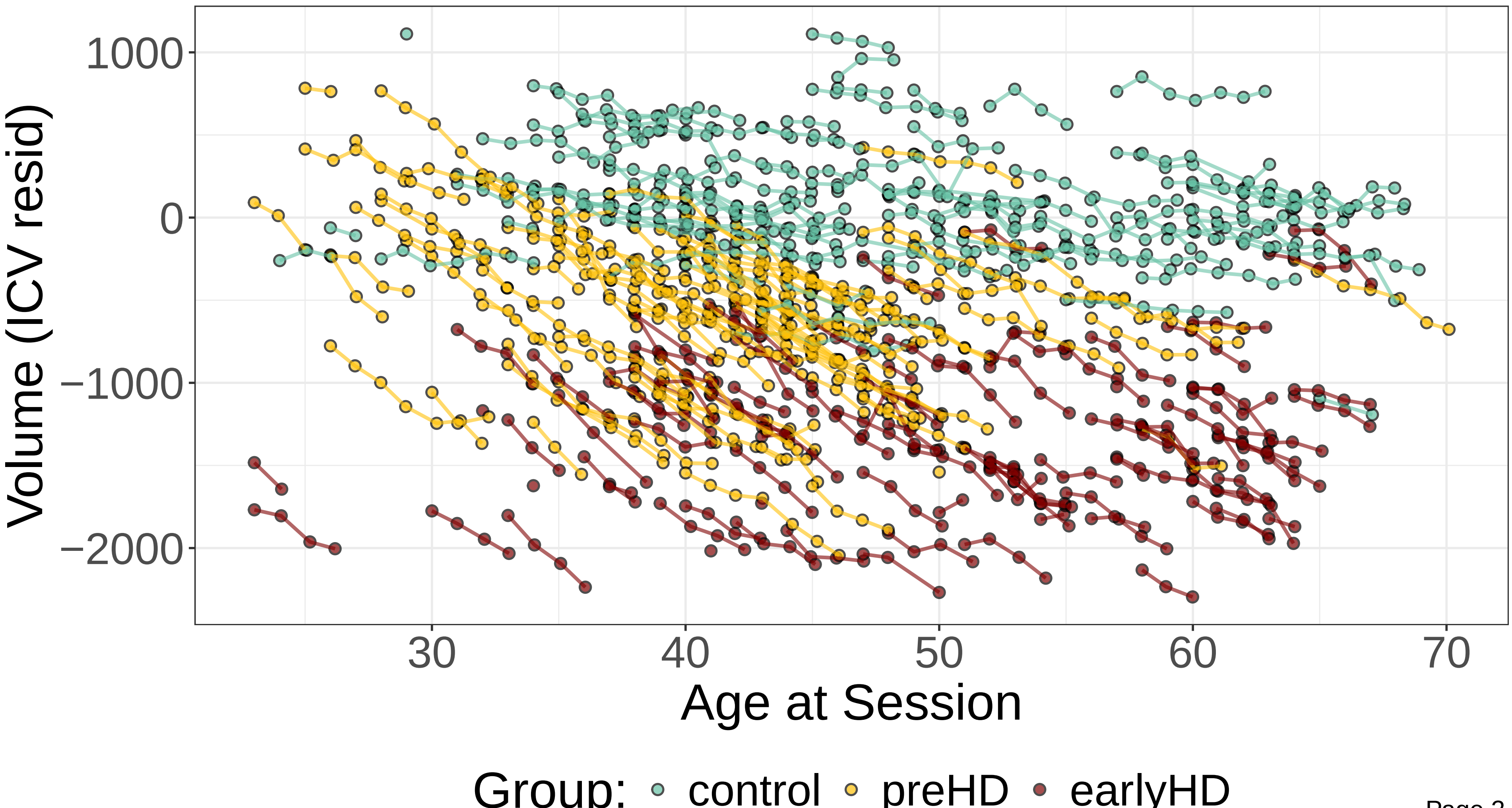

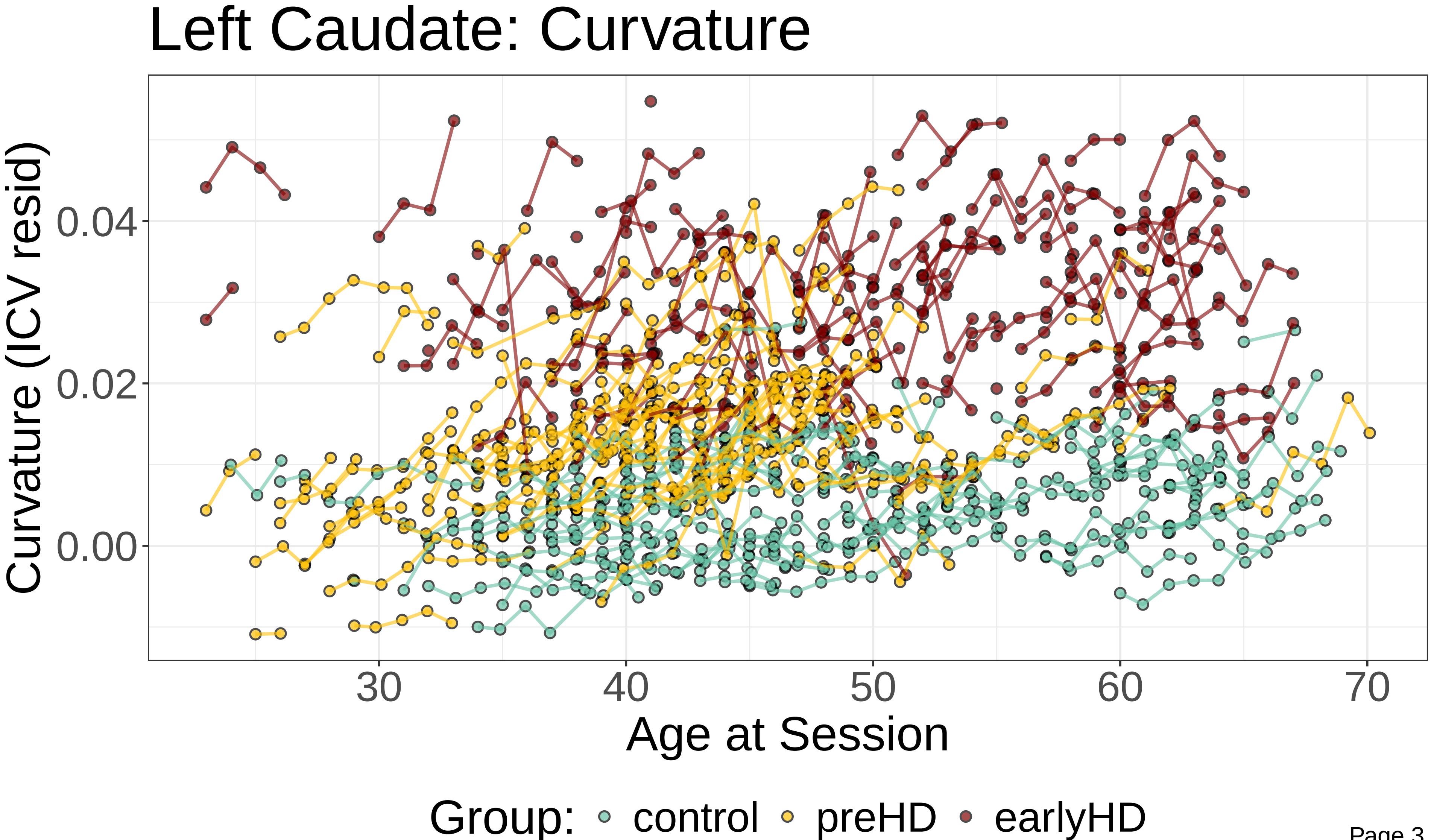

Left Caudate: Curvature
Curvature (ICV resid)
0.04
0.02
0.00
30
40
50
60
70
Age at Session
Group: control preHD earlyHD

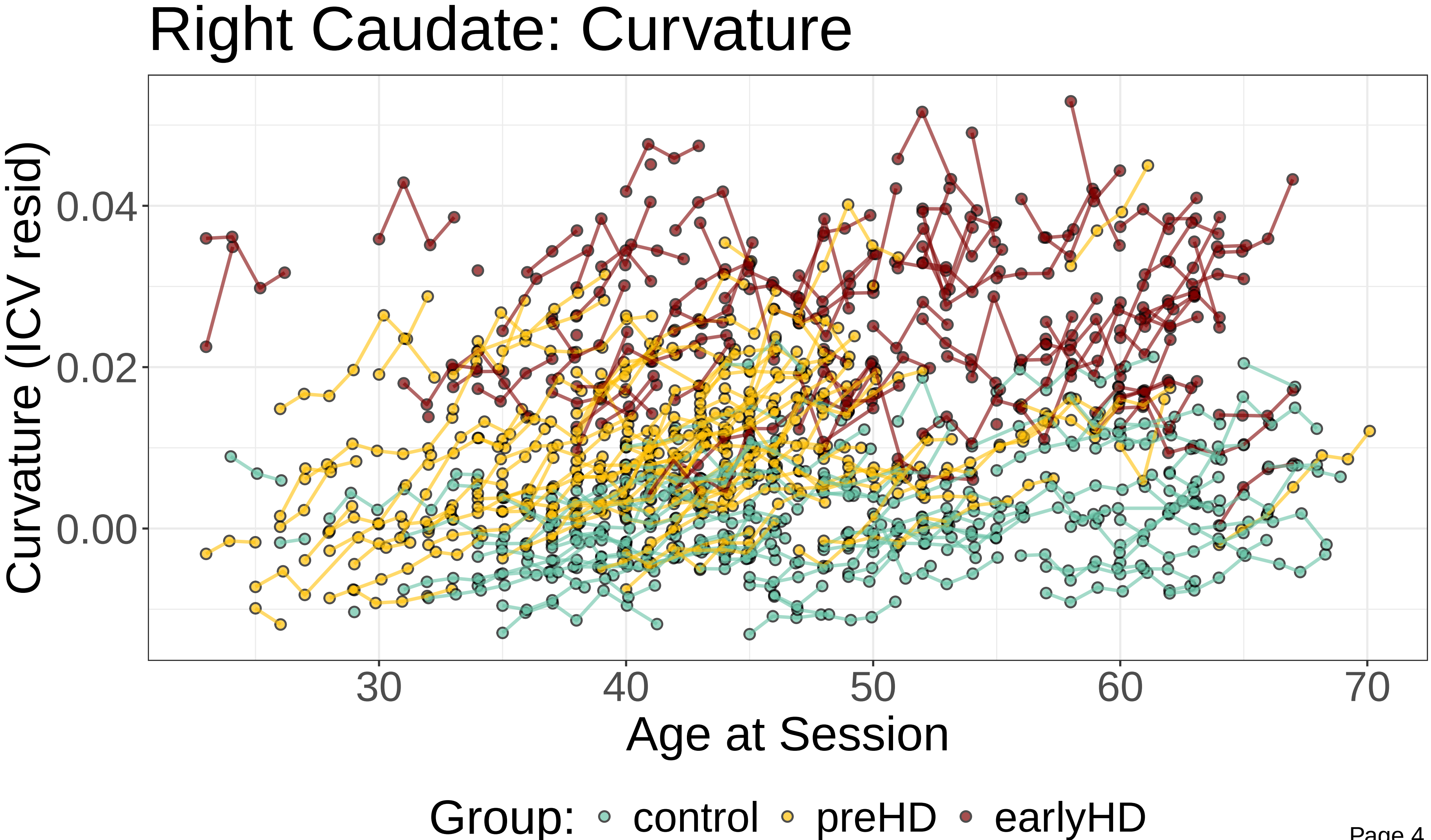
Right Caudate: Curvature
Curvature (ICV resid)
0.04
0.02
0.00
30
40
50
60
70
Age at Session
Group:
control
preHD
earlyHD

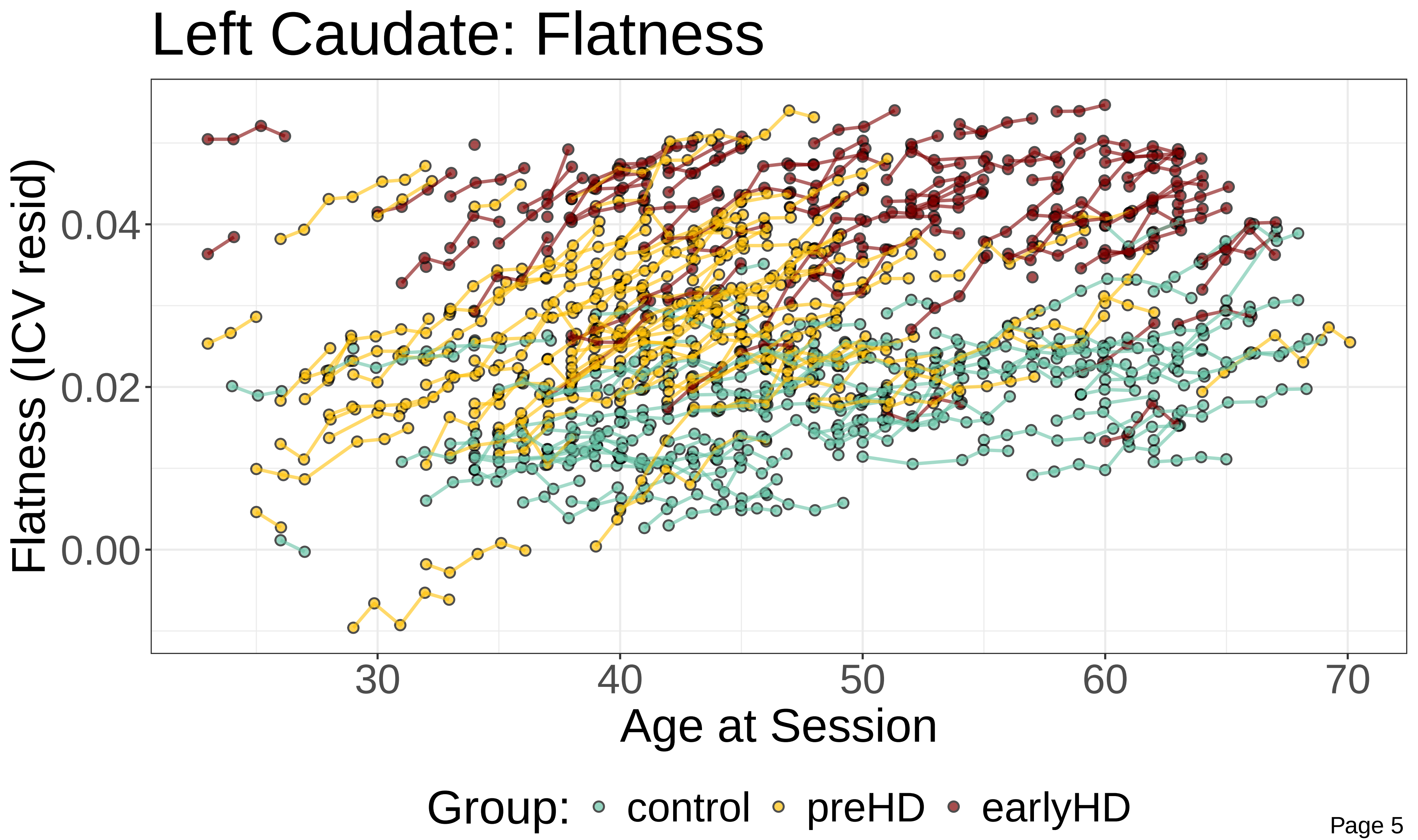
Left Caudate: Flatness
Flatness (ICV resid)
0.04
0.02
0.00
30
40
50
60
70
Age at Session
Group:
control
preHD
earlyHD

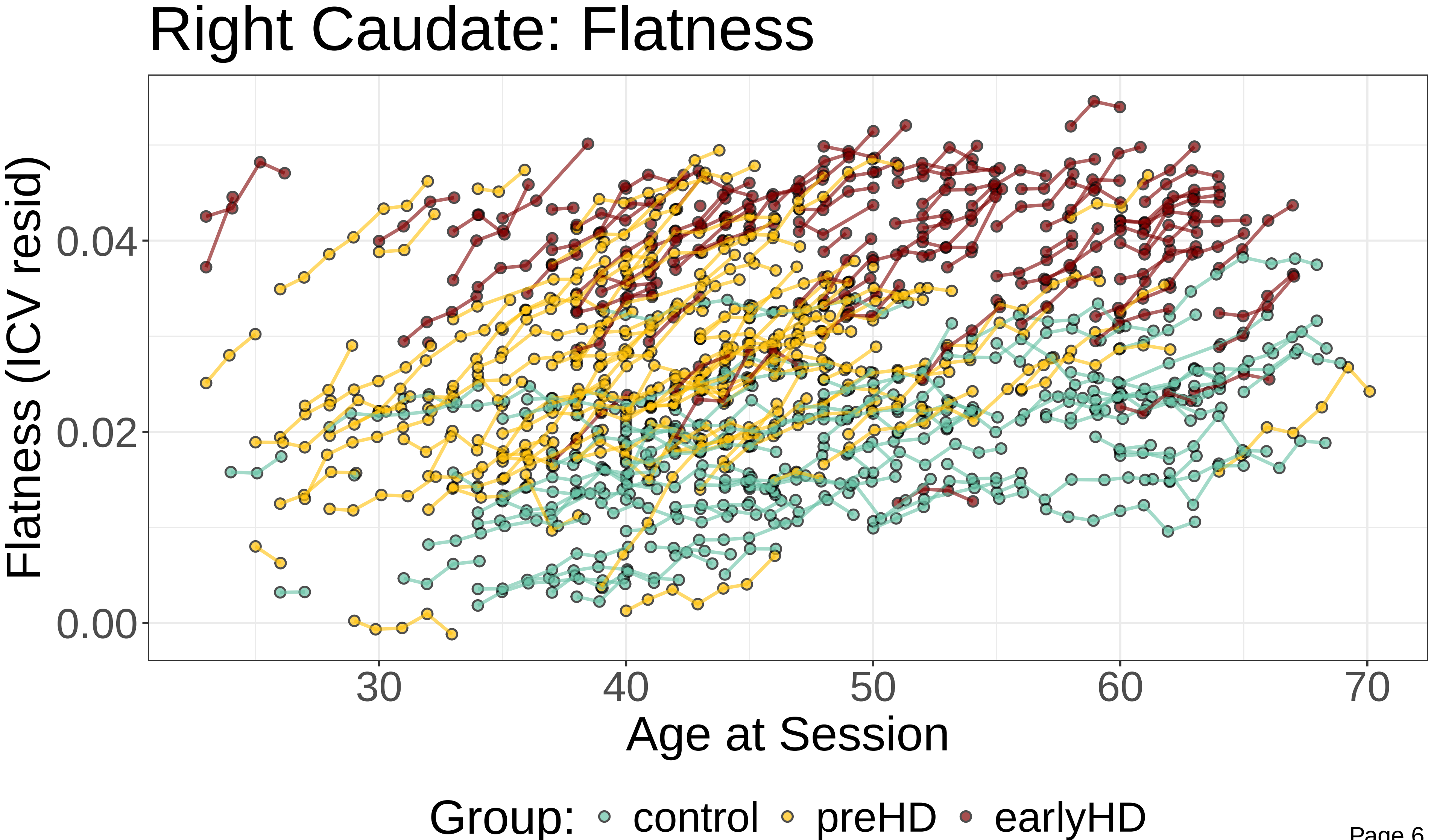
Right Caudate: Flatness
Flatness (ICV resid)
0.00
0.02
0.04
30
40
50
60
70
Age at Session
Group:
control
preHD
earlyHD

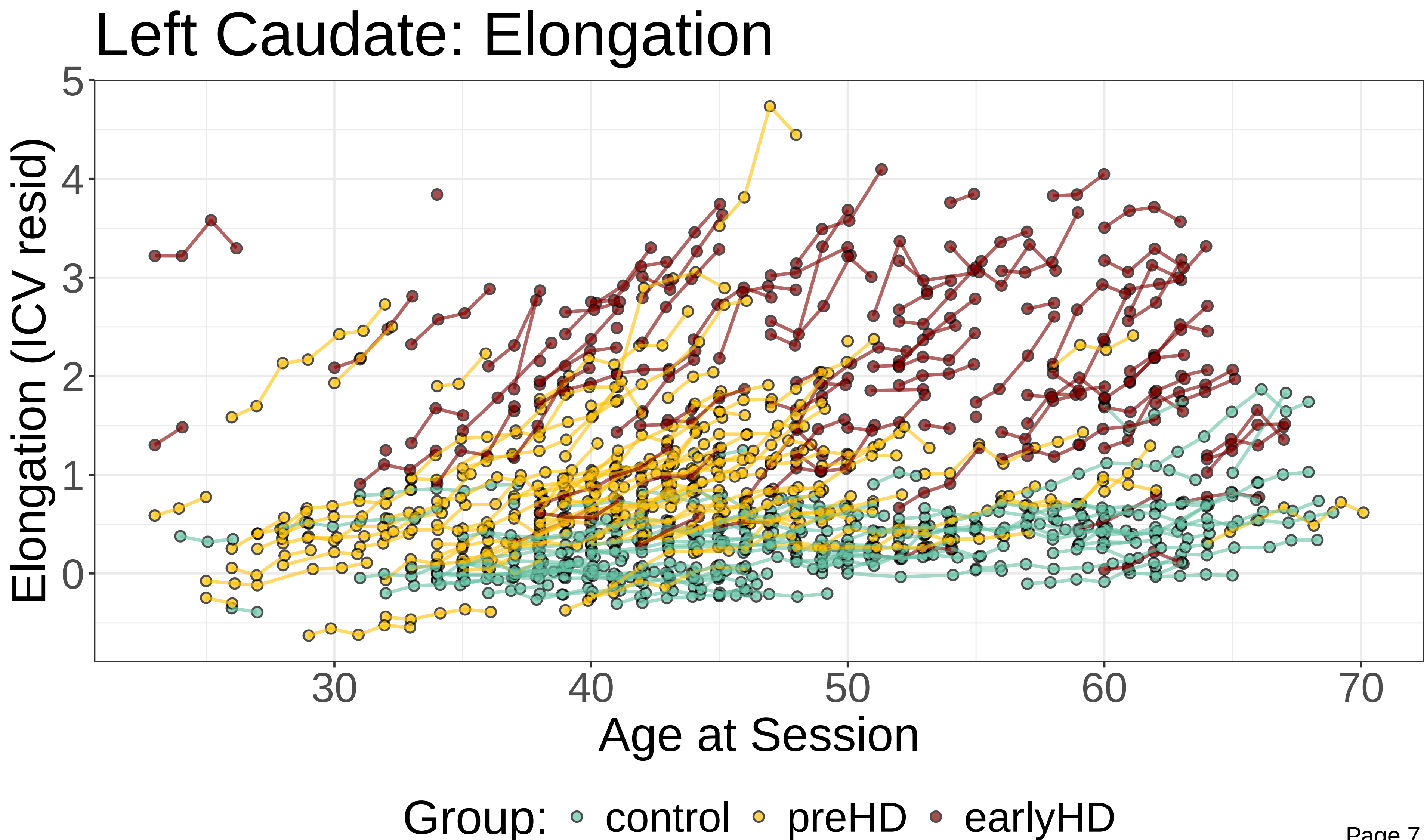
Left Caudate: Elongation
Elongation (ICV resid)
5
4
3
2
1
0
30
40
50
60
70
Age at Session
Group: control preHD earlyHD

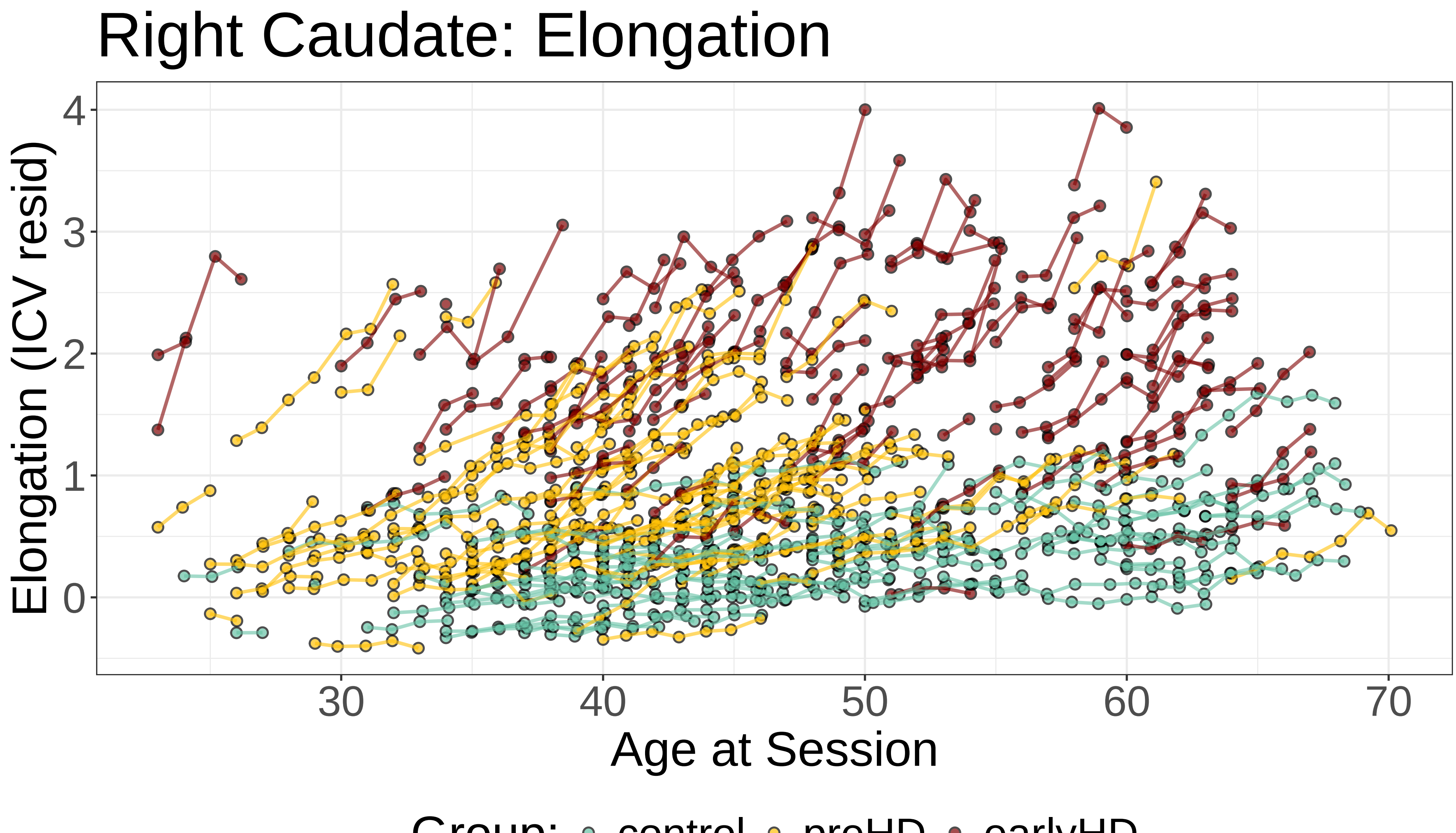
Right Caudate: Elongation
Elongation (ICV resid)
4
3
2
1
0
30
40
50
60
70
Age at Session
Group:
control
preHD
earlyHD

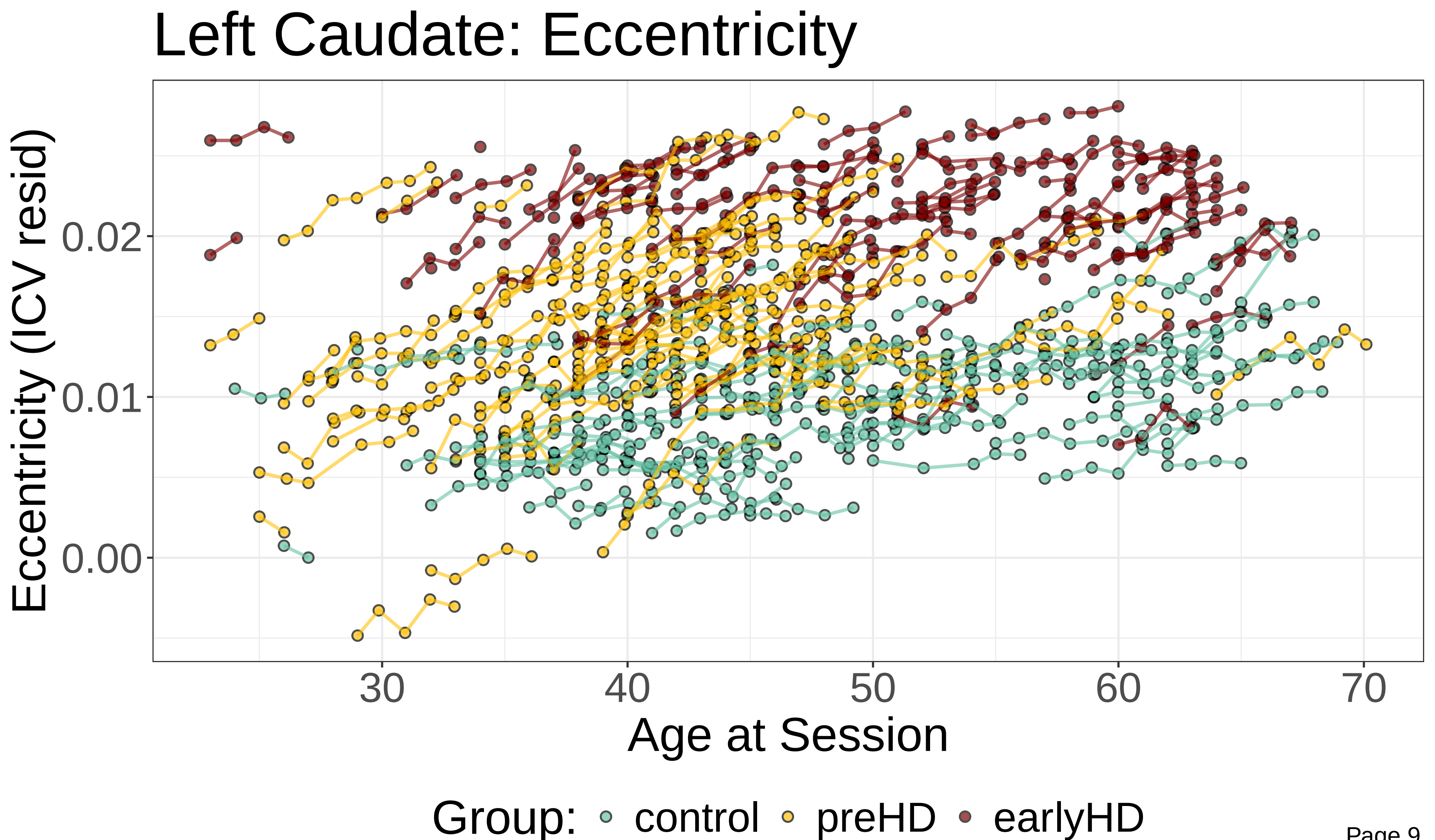
Left Caudate: Eccentricity
Eccentricity (ICV resid)
0.02
0.01
0.00
30
40
50
60
70
Age at Session
Group: control preHD earlyHD

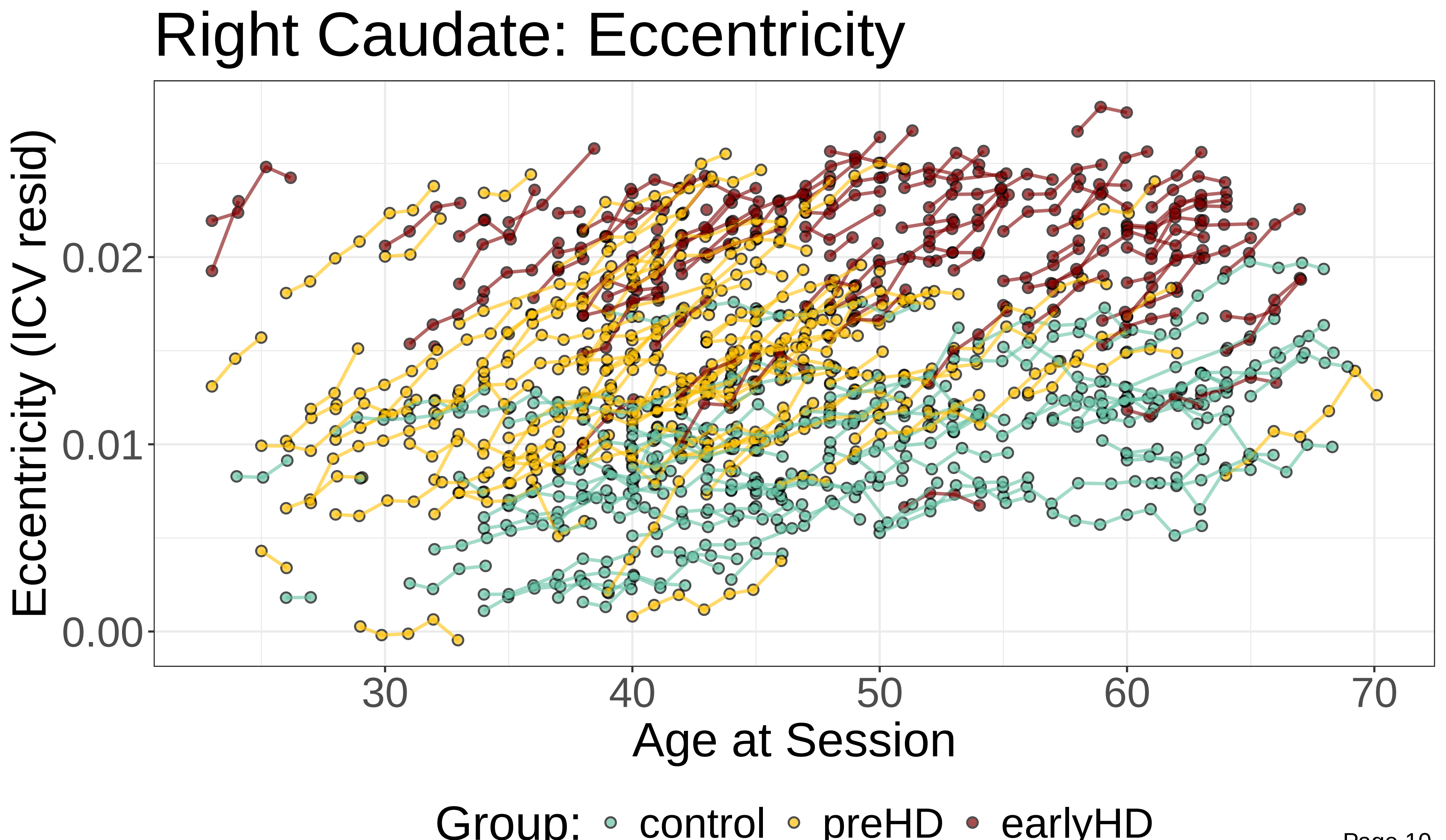

Right Caudate: Eccentricity
Eccentricity (ICV resid)
0.00
0.01
0.02
30
40
50
60
70
Age at Session
Group: control preHD earlyHD

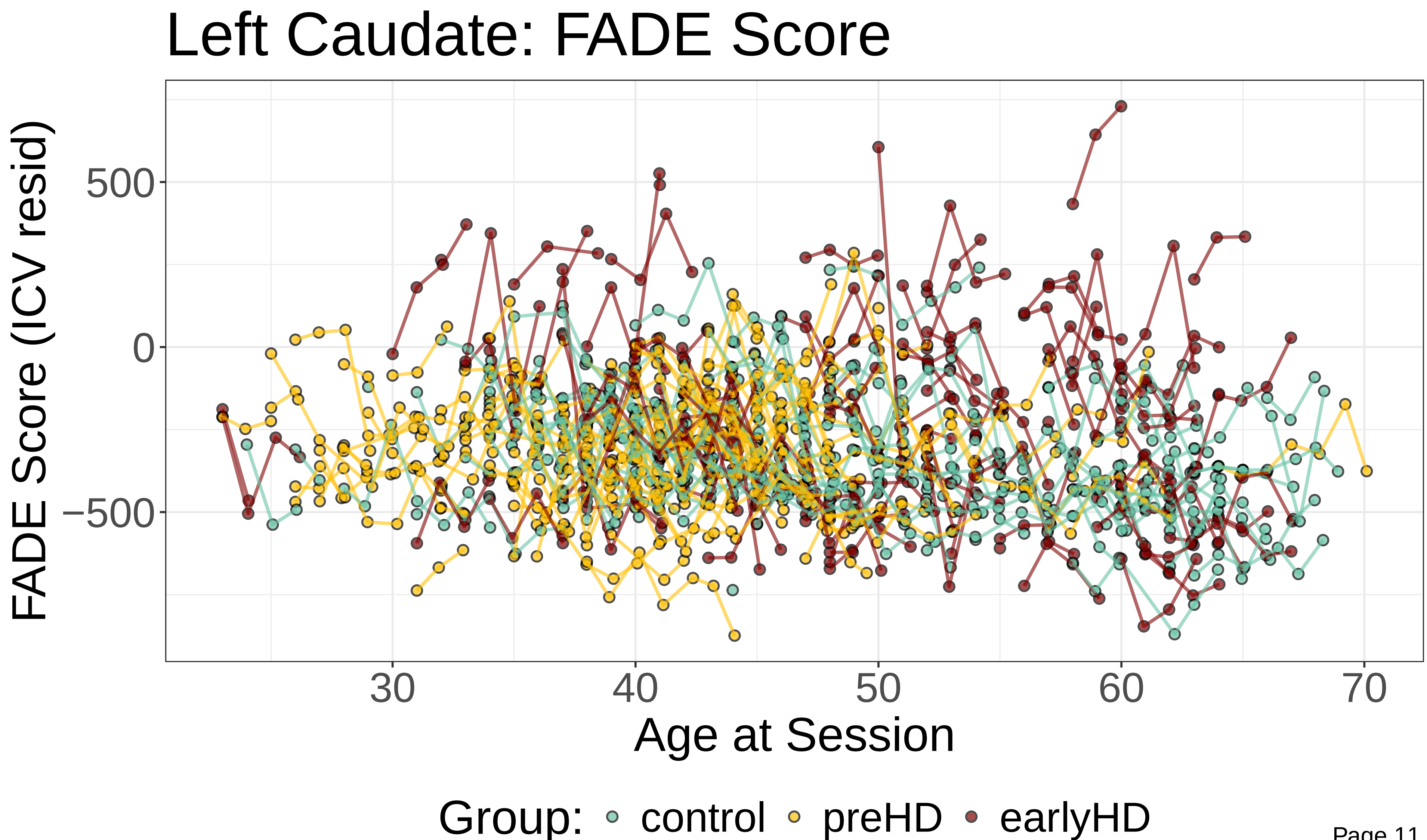

Left Caudate: FADE Score
FADE Score (ICV resid)
500
0
−500
30
40
50
60
70
Age at Session
Group:
control
preHD
earlyHD

Right Caudate: FADE Score

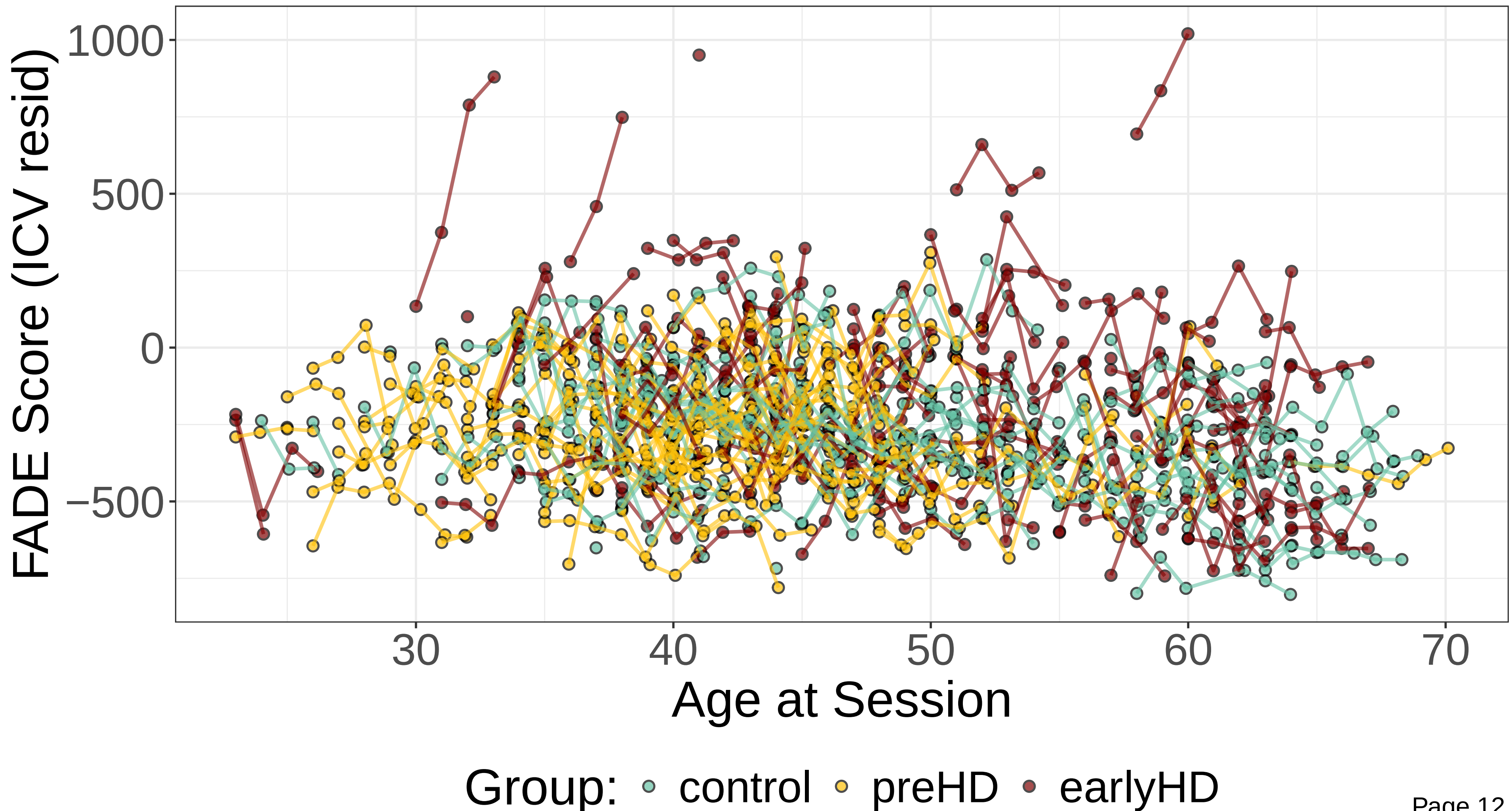

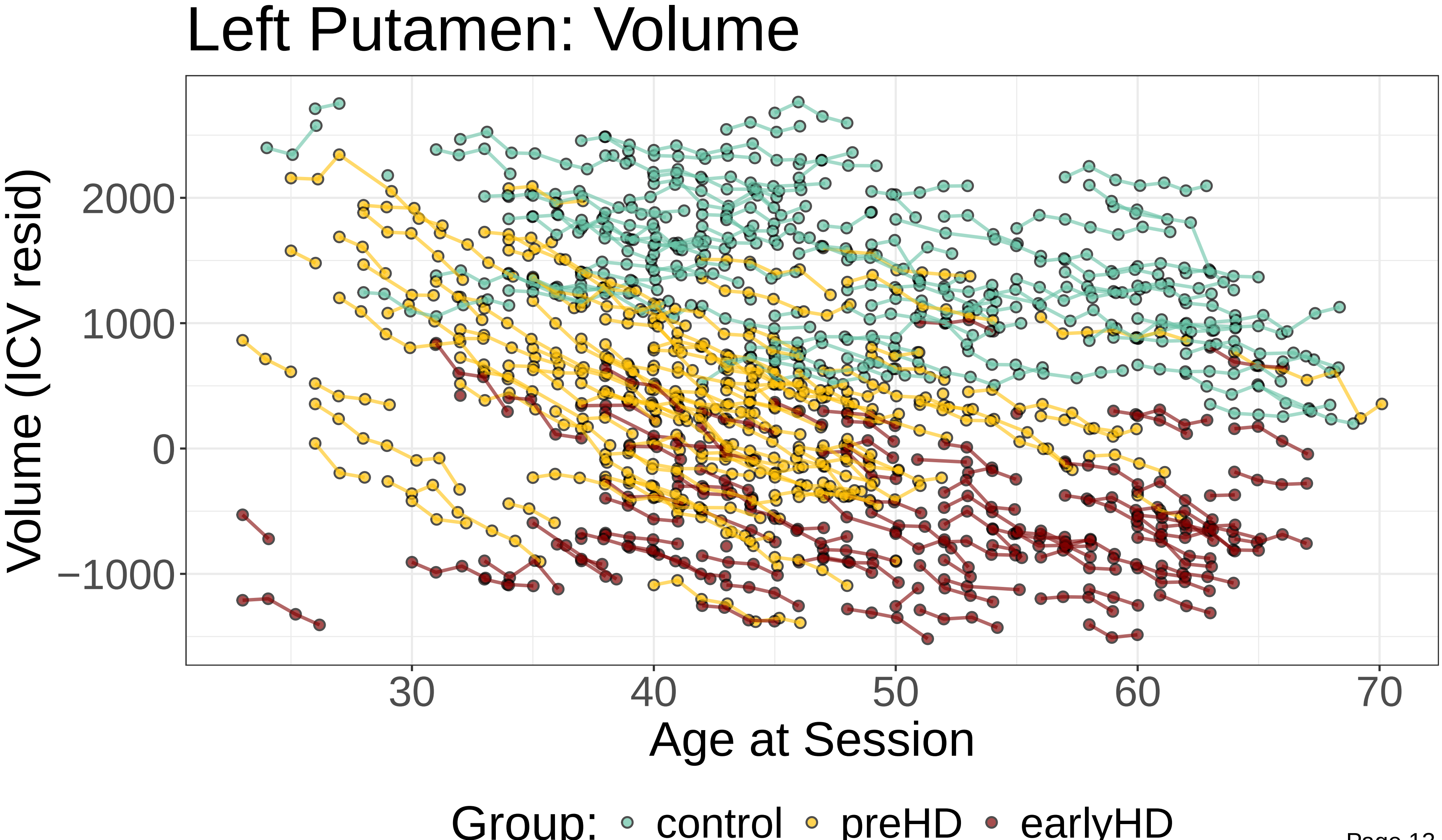
Left Putamen: Volume
Volume (ICV resid)
2000
1000
0
−1000
30
40
50
60
70
Age at Session
Group: control preHD earlyHD

Right Putamen: Volume

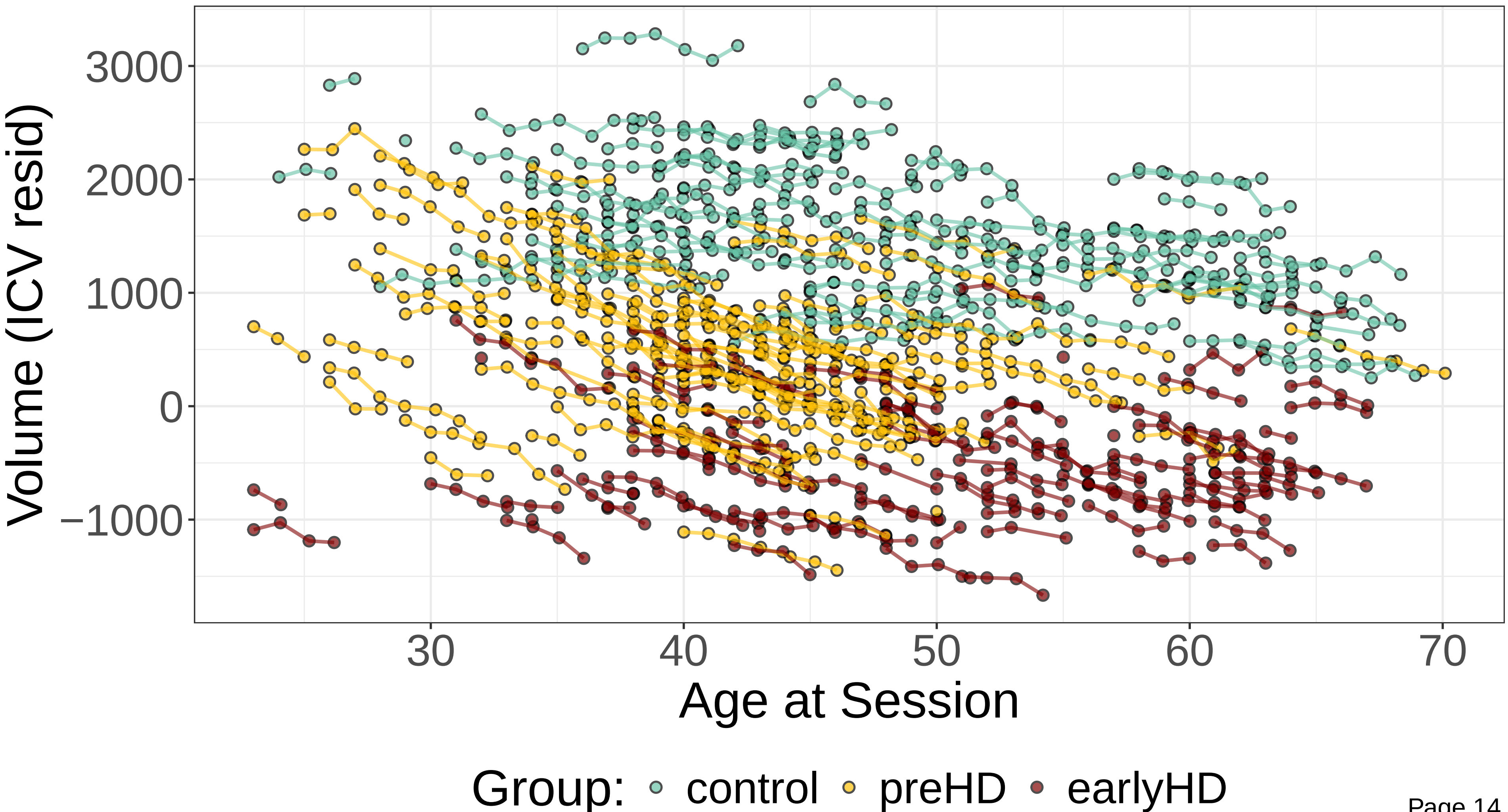

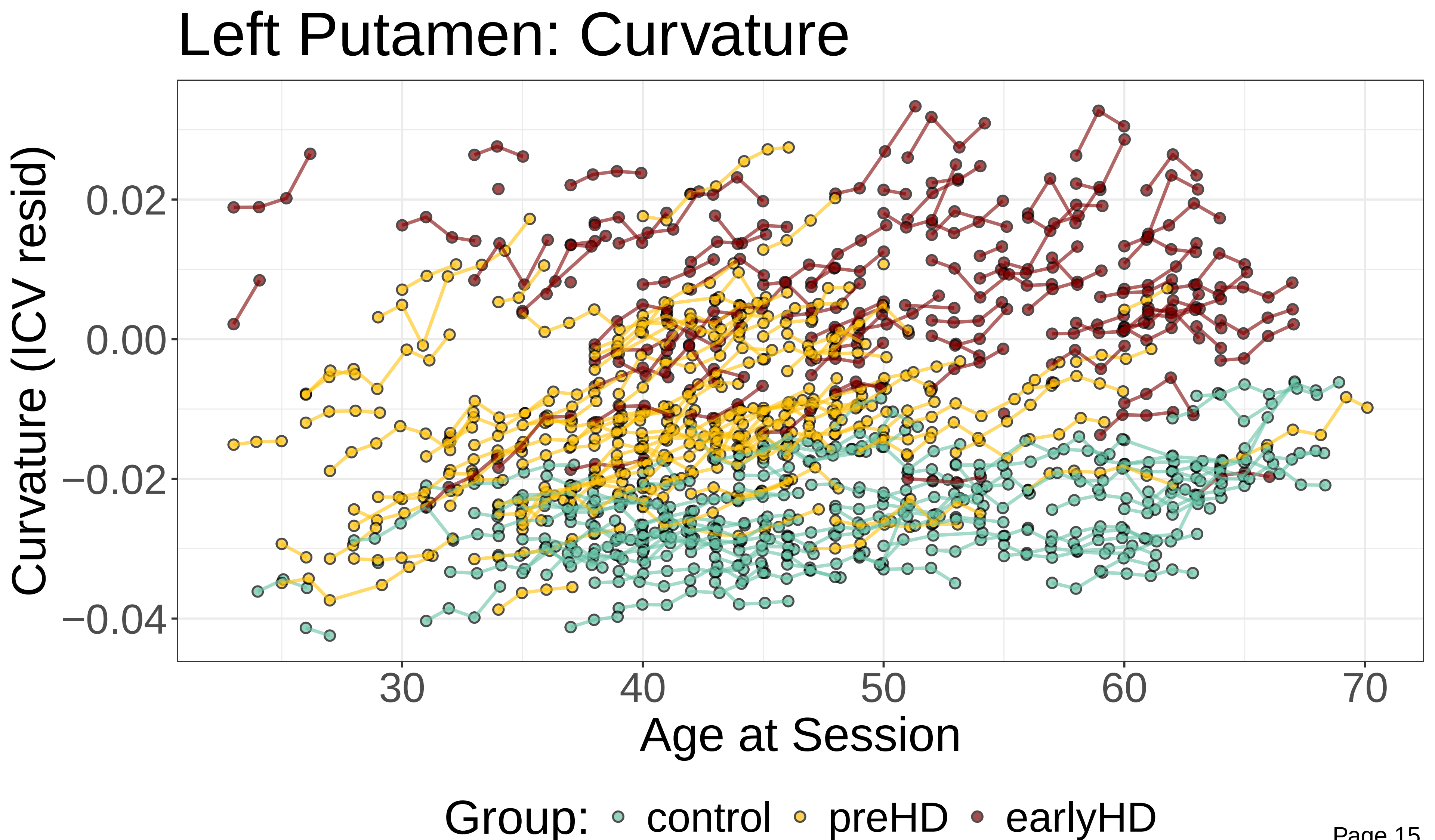

Left Putamen: Curvature
Curvature (ICV resid)
0.02
0.00
−0.02
−0.04
30
40
50
60
70
Age at Session
Group: control preHD earlyHD

Right Putamen: Curvature

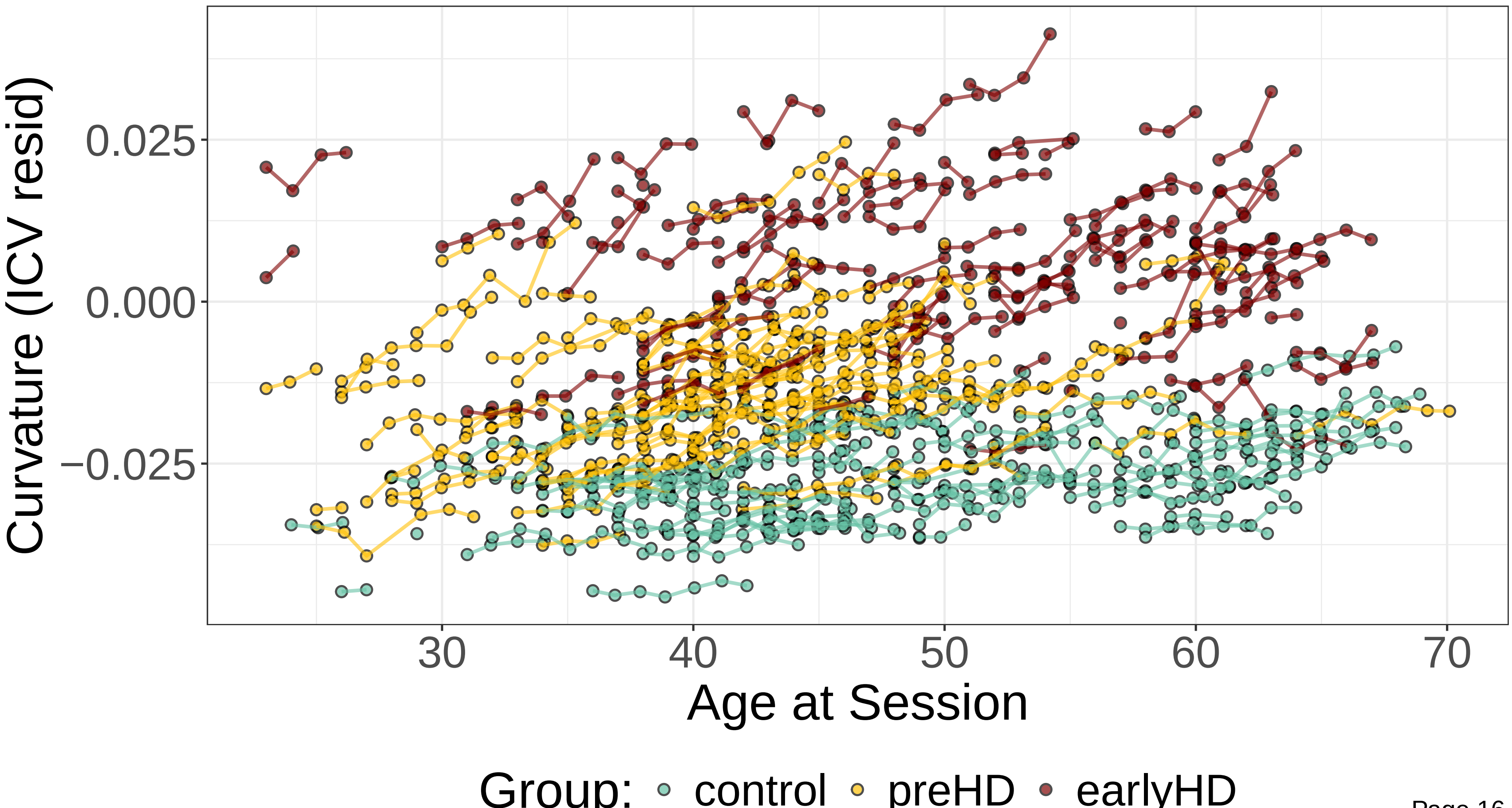

Left Putamen: Flatness

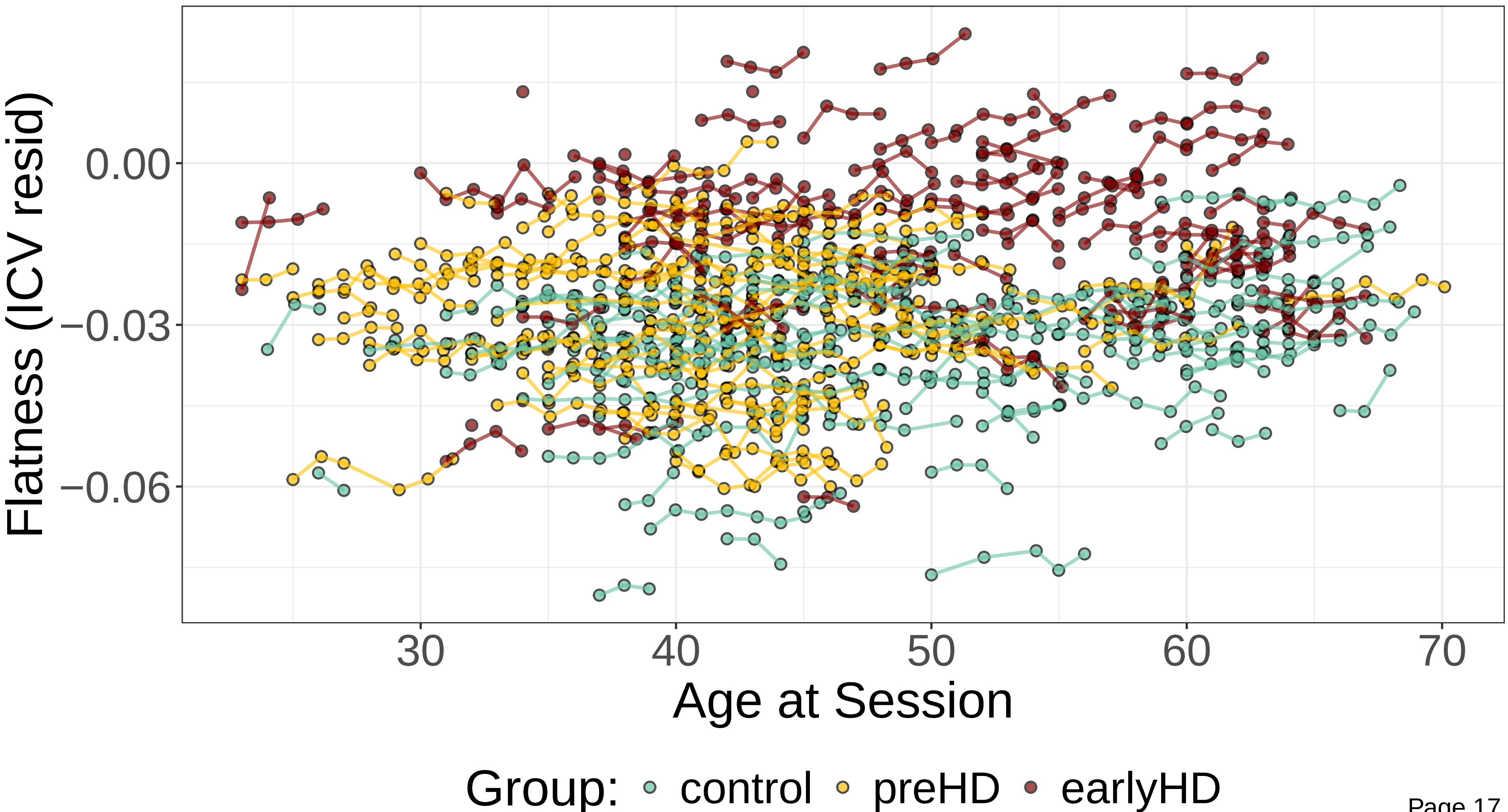

Right Putamen: Flatness

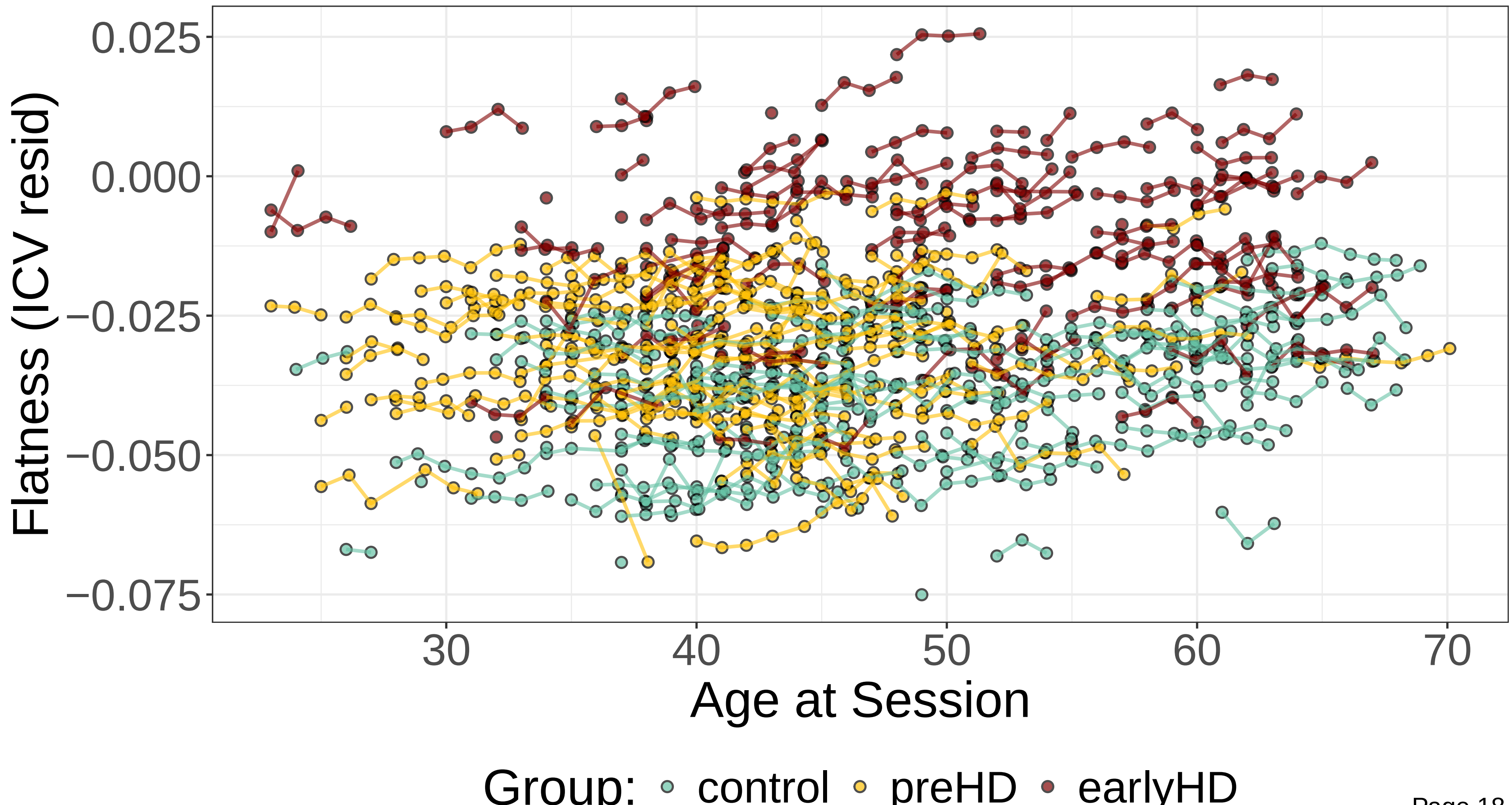

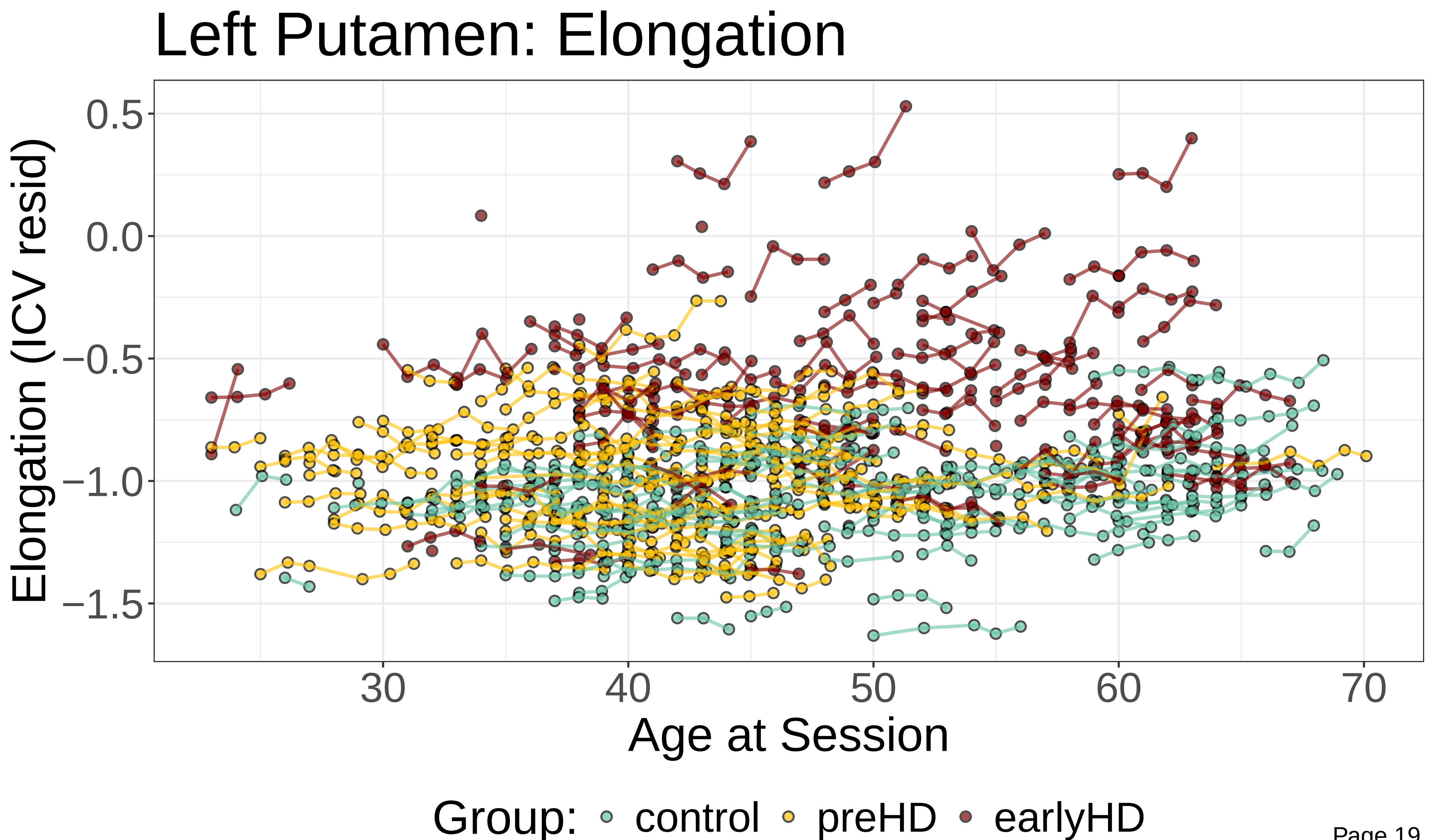
Left Putamen: Elongation
Elongation (ICV resid)
0.5
0.0
−0.5
−1.0
−1.5
30
40
50
60
70
Age at Session
Group:
control
preHD
earlyHD

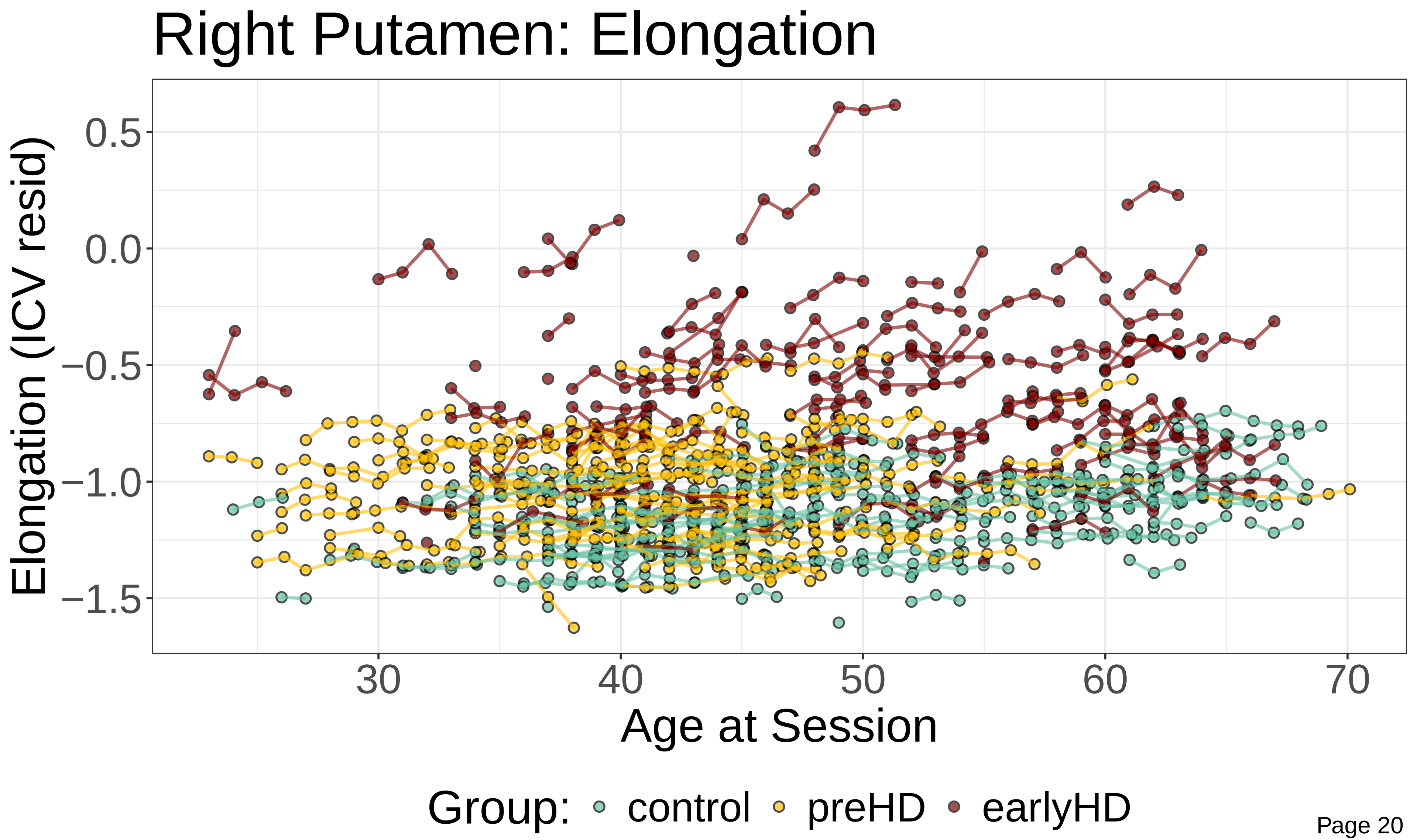
Right Putamen: Elongation
Elongation (ICV resid)
0.5
0.0
−0.5
−1.0
−1.5
30
40
50
60
70
Age at Session
Group:
control
preHD
earlyHD

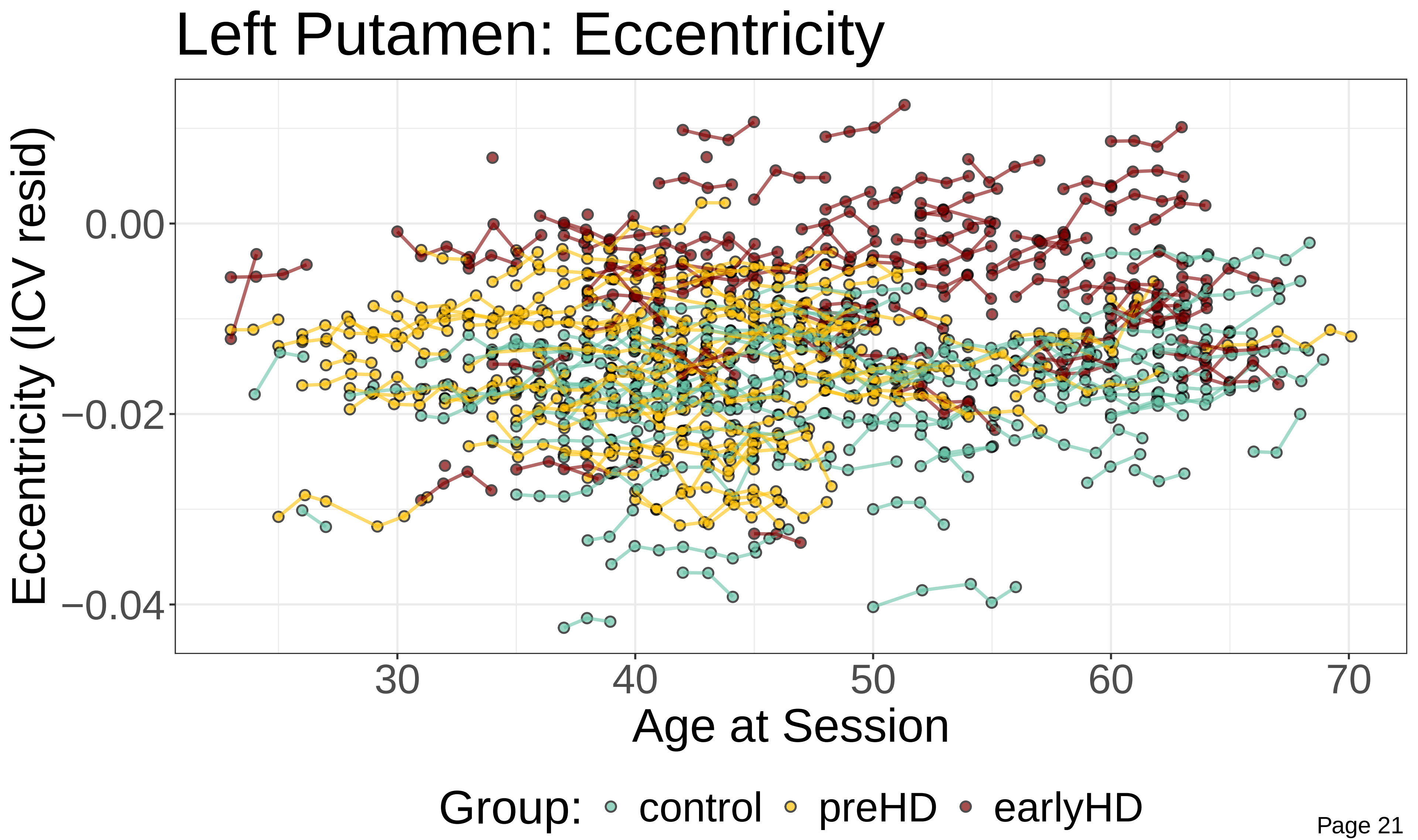

Left Putamen: Eccentricity
Eccentricity (ICV resid)
0.00
−0.02
−0.04
30
40
50
60
70
Age at Session
Group:
control
preHD
earlyHD

Right Putamen: Eccentricity

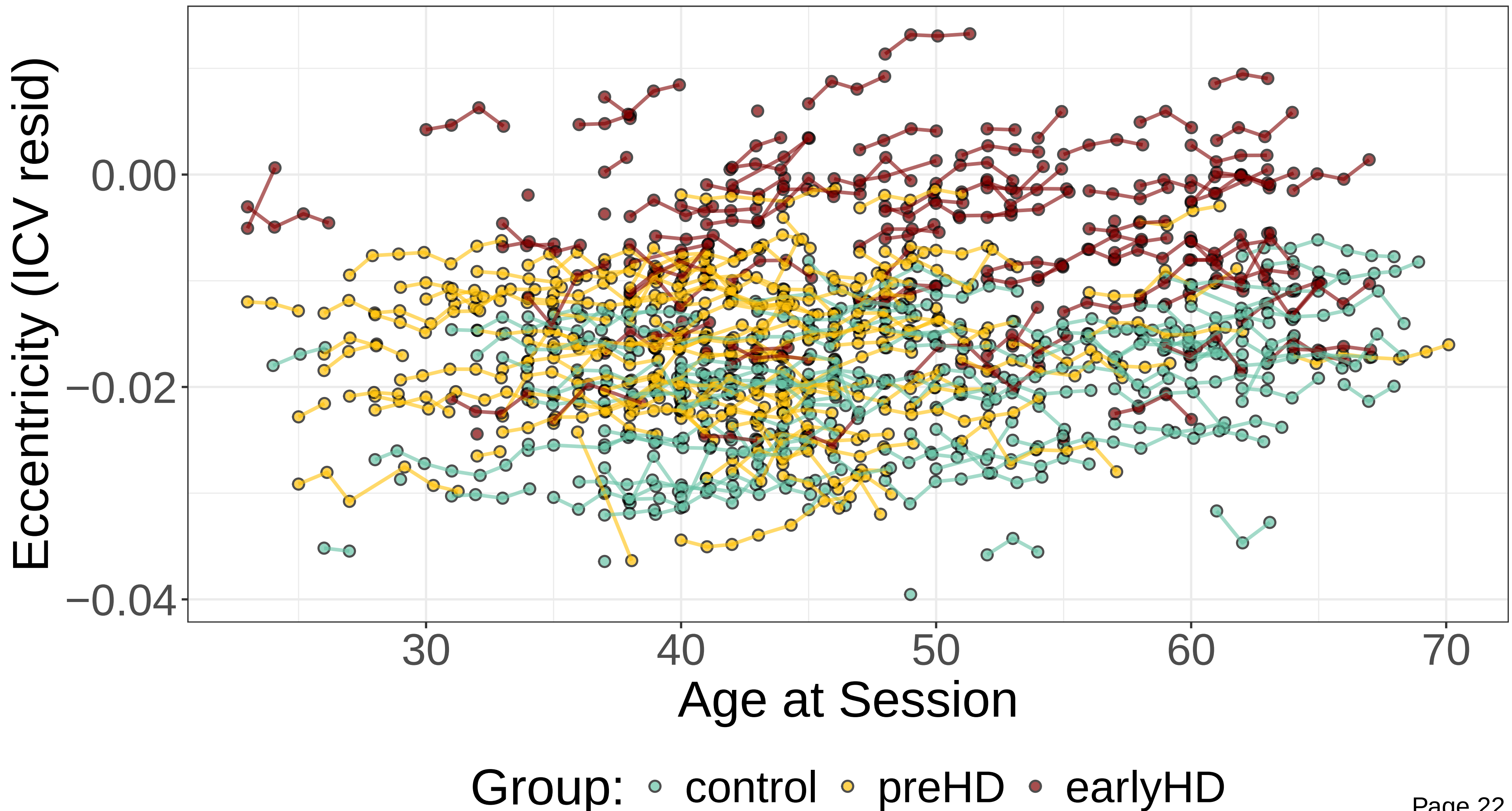

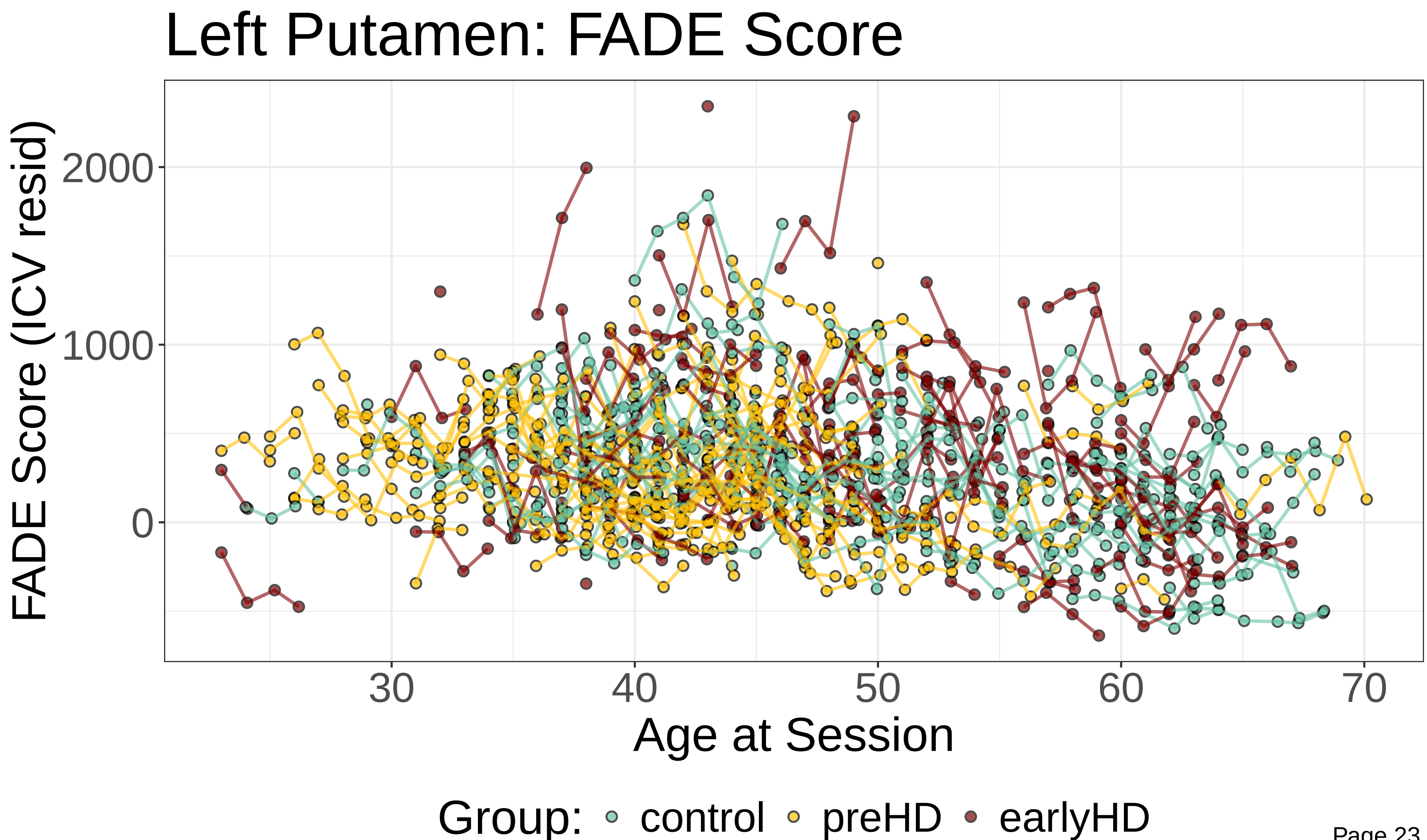

Left Putamen: FADE Score
FADE Score (ICV resid)
2000
1000
0
30
40
50
60
70
Age at Session
Group:
control
preHD
earlyHD

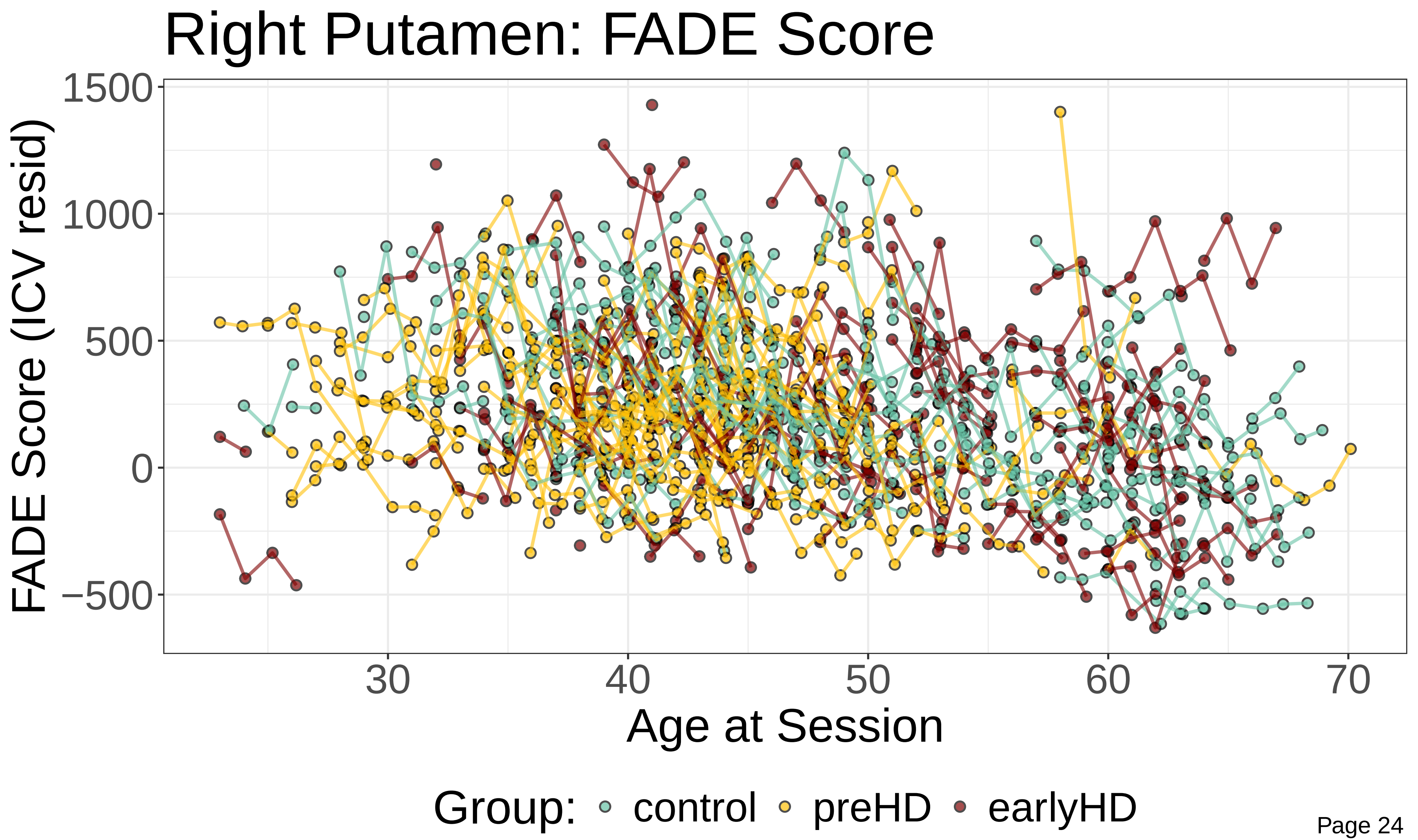

Right Putamen: FADE Score
FADE Score (ICV resid)
1500
1000
500
0
−500
30
40
50
60
70
Age at Session
Group: control preHD earlyHD

Supplementary File - Spaghetti Plots by CAP score

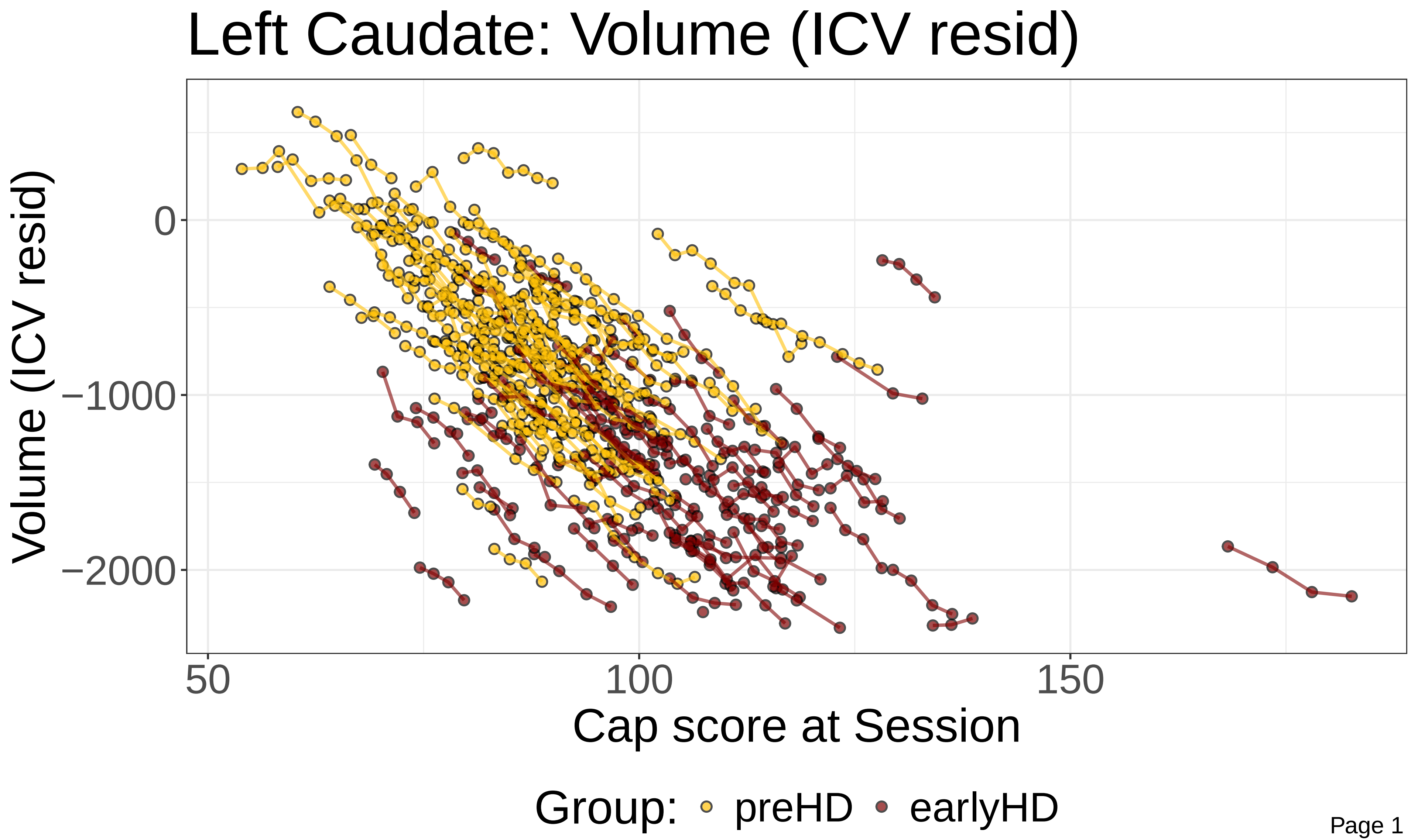
Left Caudate: Volume (ICV resid)
Volume (ICV resid)
0
−1000
−2000
50
100
150
Cap score at Session
Group: preHD earlyHD

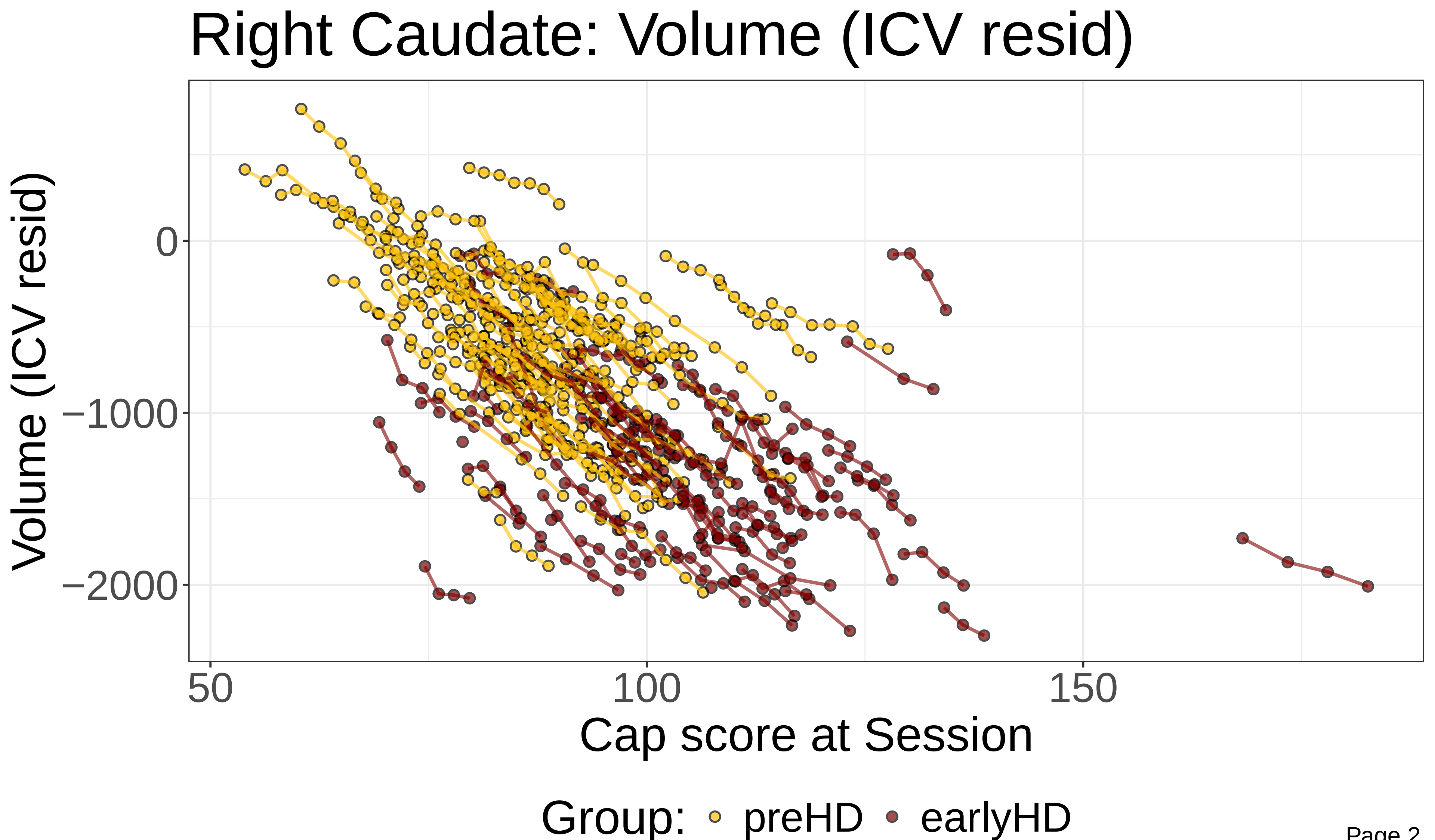

Right Caudate: Volume (ICV resid)
Volume (ICV resid)
0
−1000
−2000
50
100
150
Cap score at Session
Group: preHD earlyHD

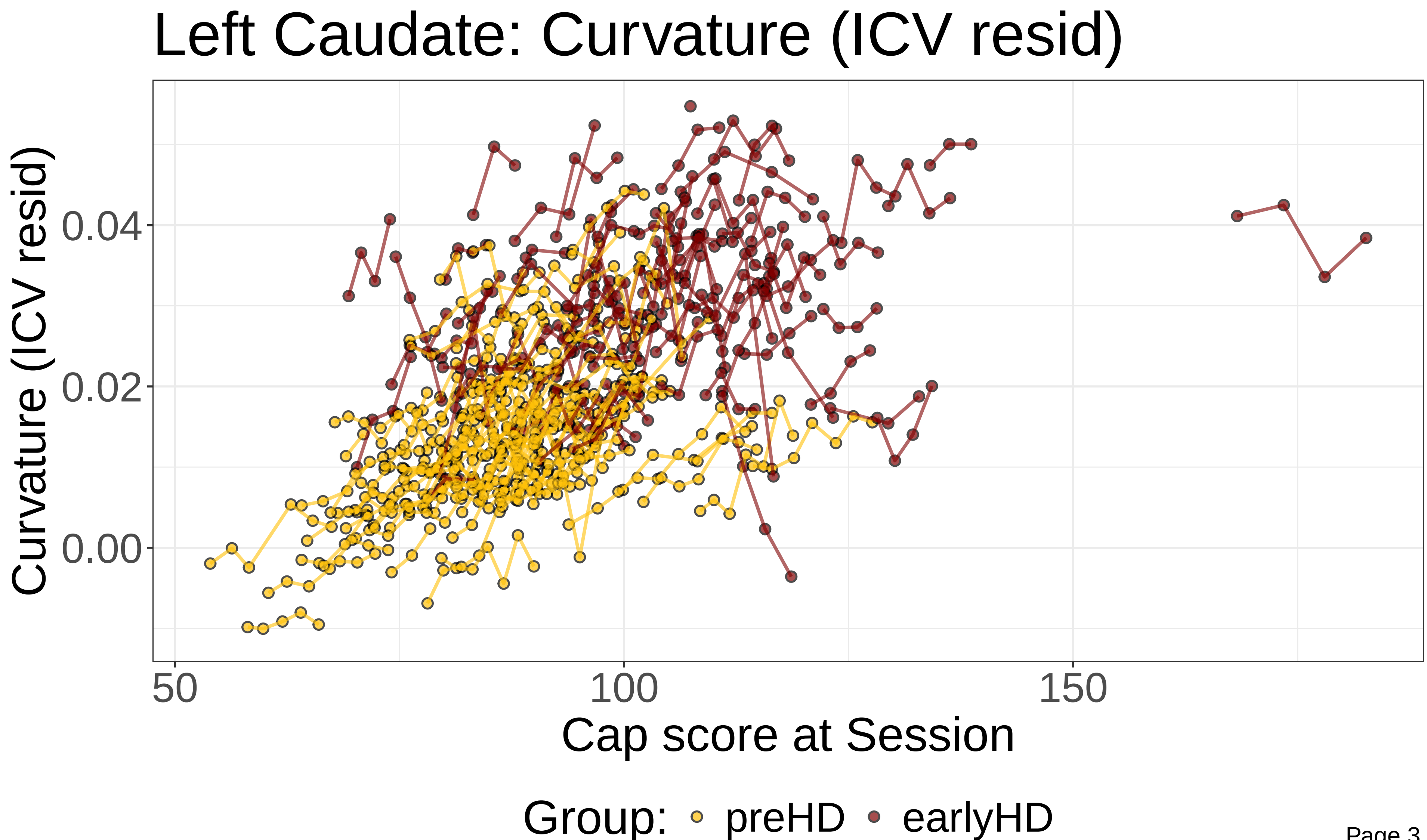

Left Caudate: Curvature (ICV resid)
Curvature (ICV resid)
0.04
0.02
0.00
50
100
150
Cap score at Session
Group: preHD earlyHD

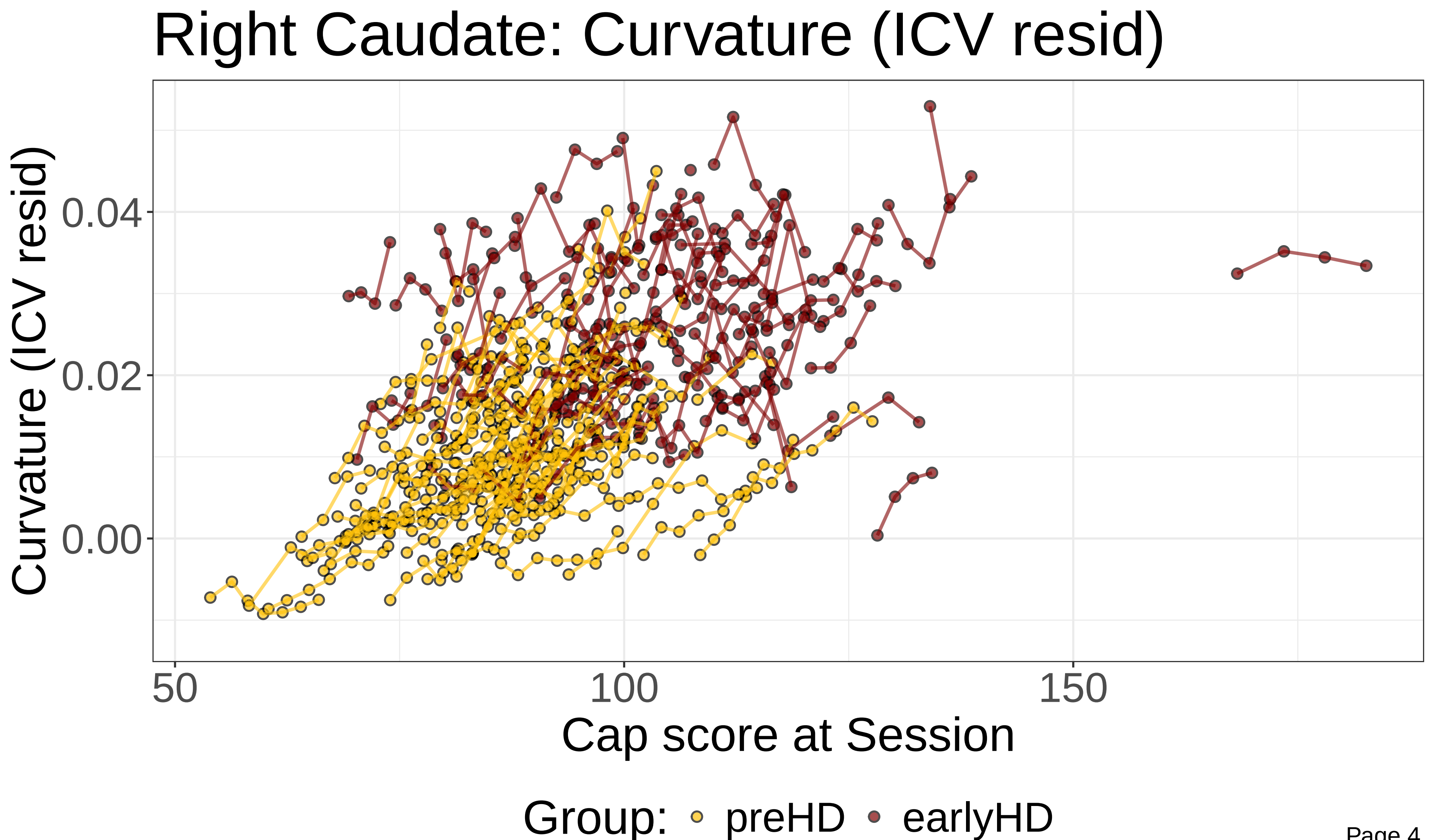

Right Caudate: Curvature (ICV resid)
Curvature (ICV resid)
0.04
0.02
0.00
50
100
150
Cap score at Session
Group: preHD earlyHD

Left Caudate: Flatness (ICV resid)

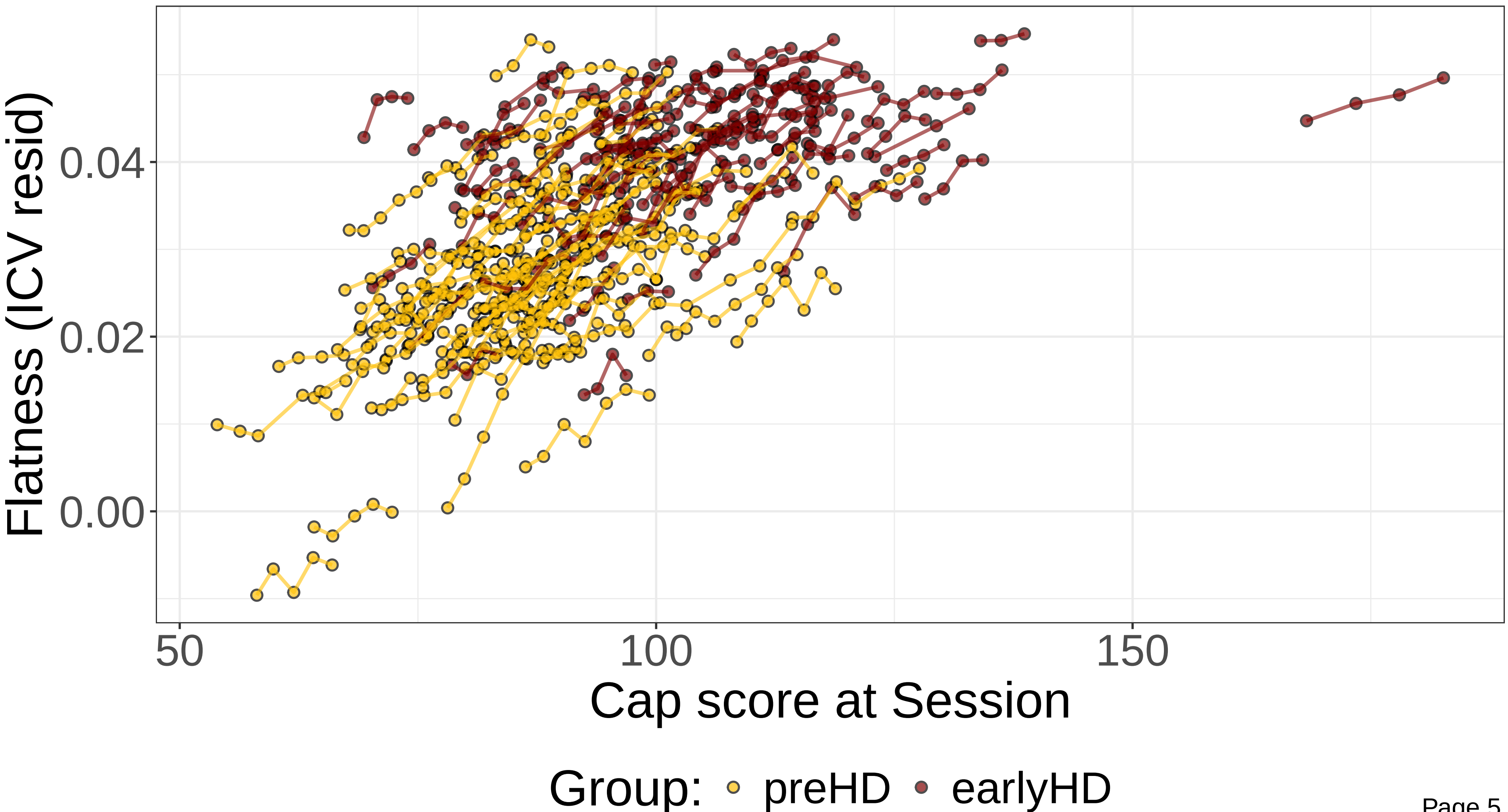

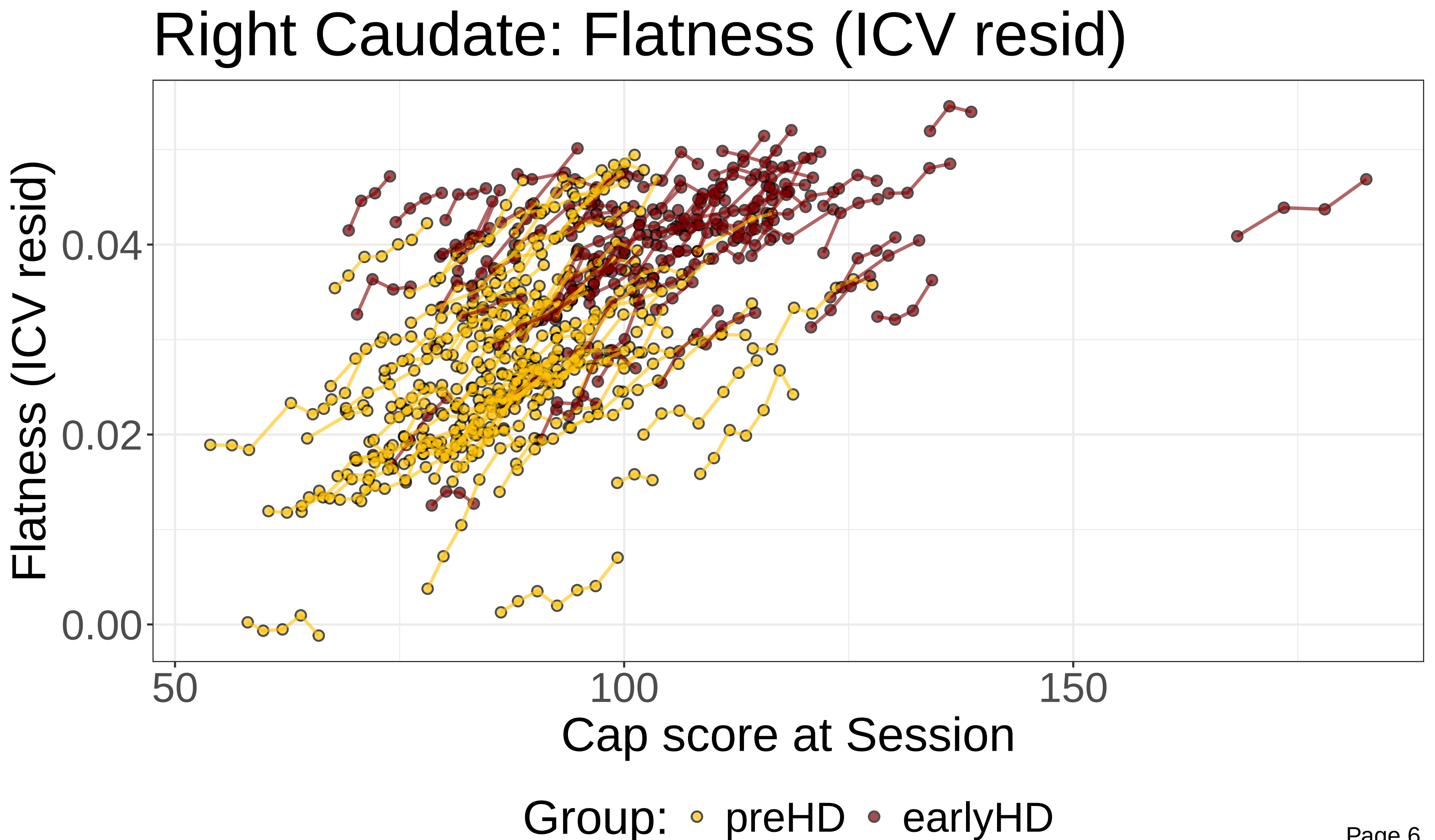
Right Caudate: Flatness (ICV resid)
Flatness (ICV resid)
0.00
0.02
0.04
50
100
150
Cap score at Session
Group: preHD earlyHD

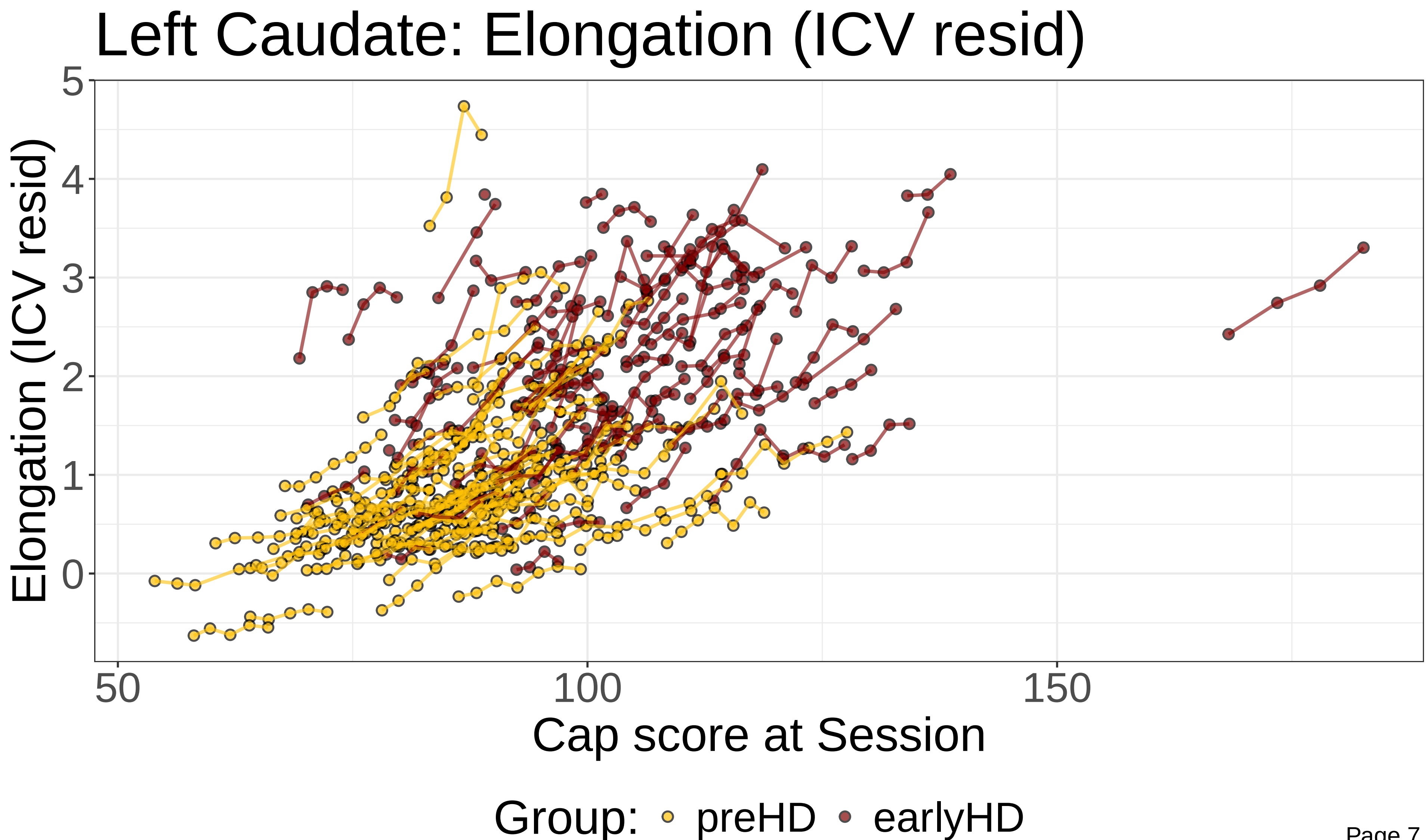
Left Caudate: Elongation (ICV resid)
Elongation (ICV resid)
5
4
3
2
1
0
50
100
150
Cap score at Session
Group:
preHD
earlyHD

Right Caudate: Elongation (ICV resid)

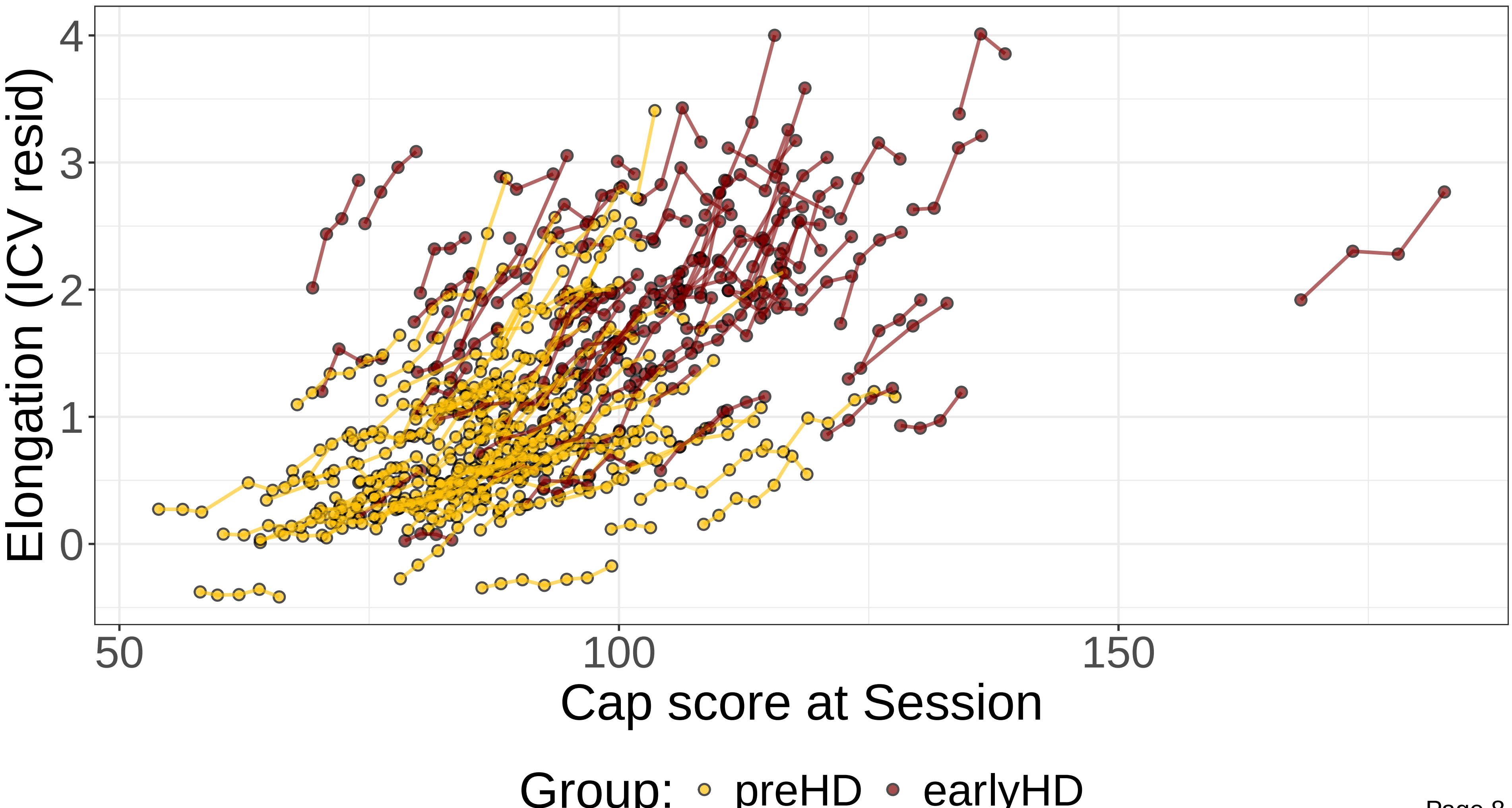

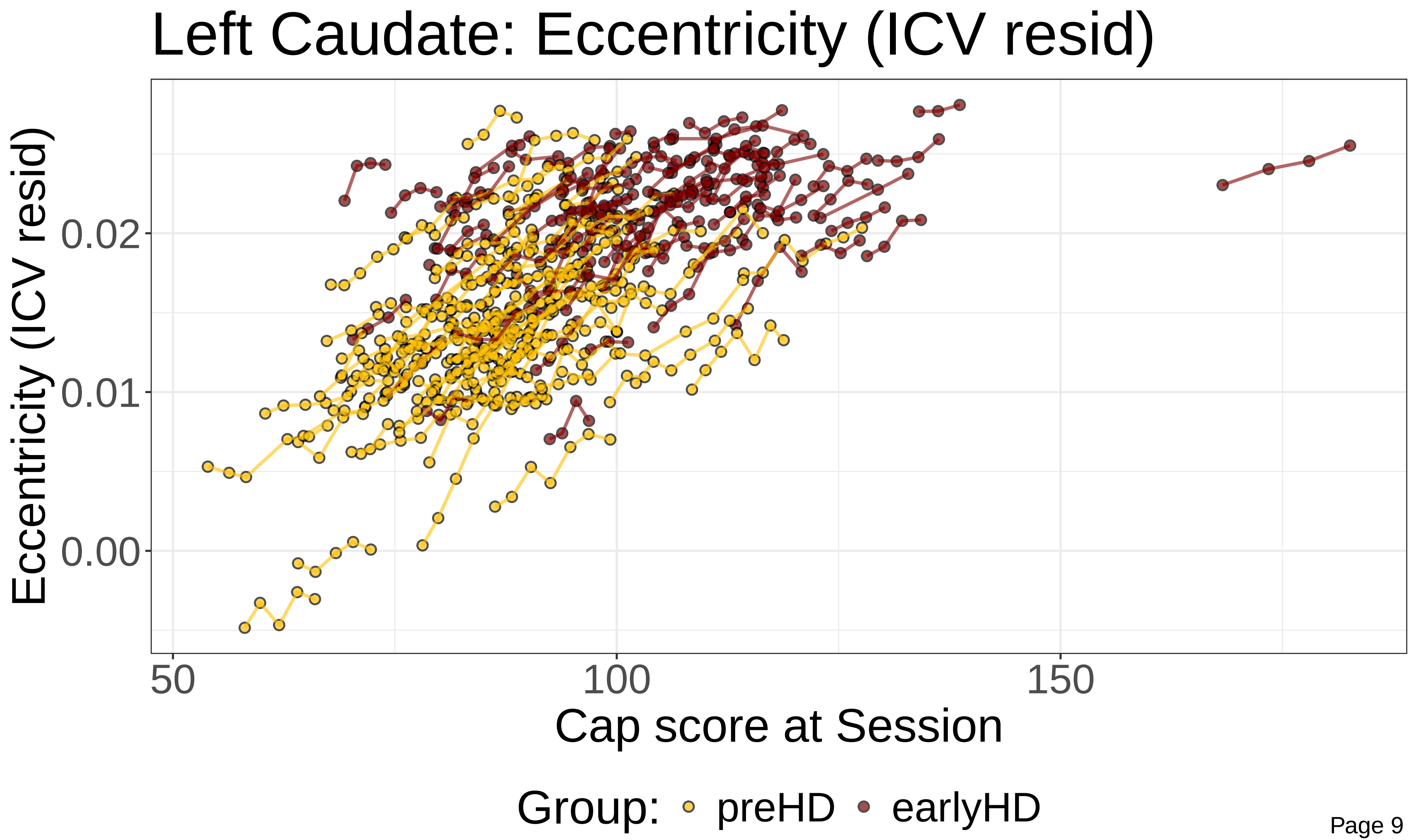
Left Caudate: Eccentricity (ICV resid)
Eccentricity (ICV resid)
0.00
0.01
0.02
50
100
150
Cap score at Session
Group:
preHD
earlyHD

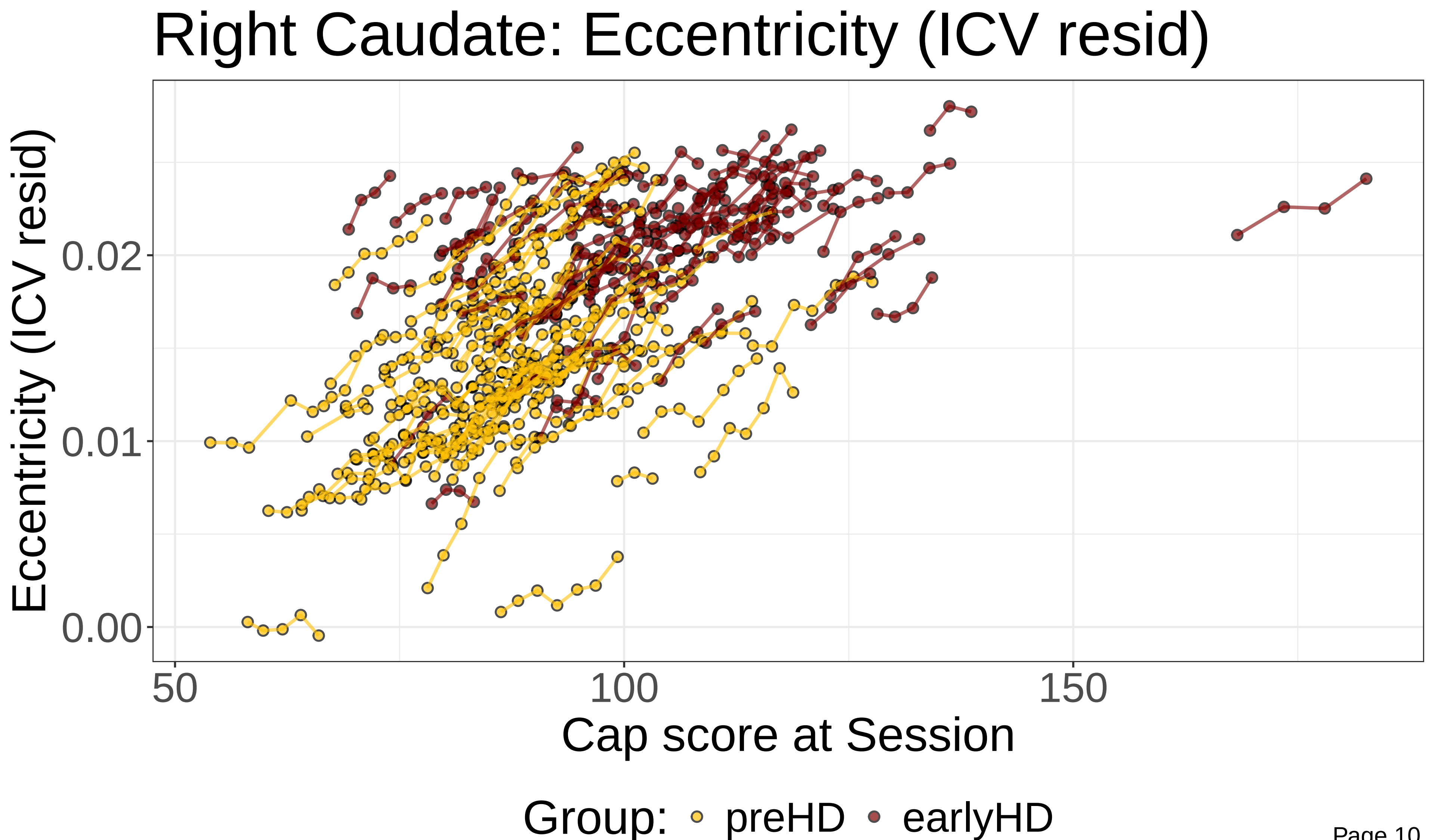
Right Caudate: Eccentricity (ICV resid)
Eccentricity (ICV resid)
0.00
0.01
0.02
50
100
150
Cap score at Session
Group: preHD earlyHD

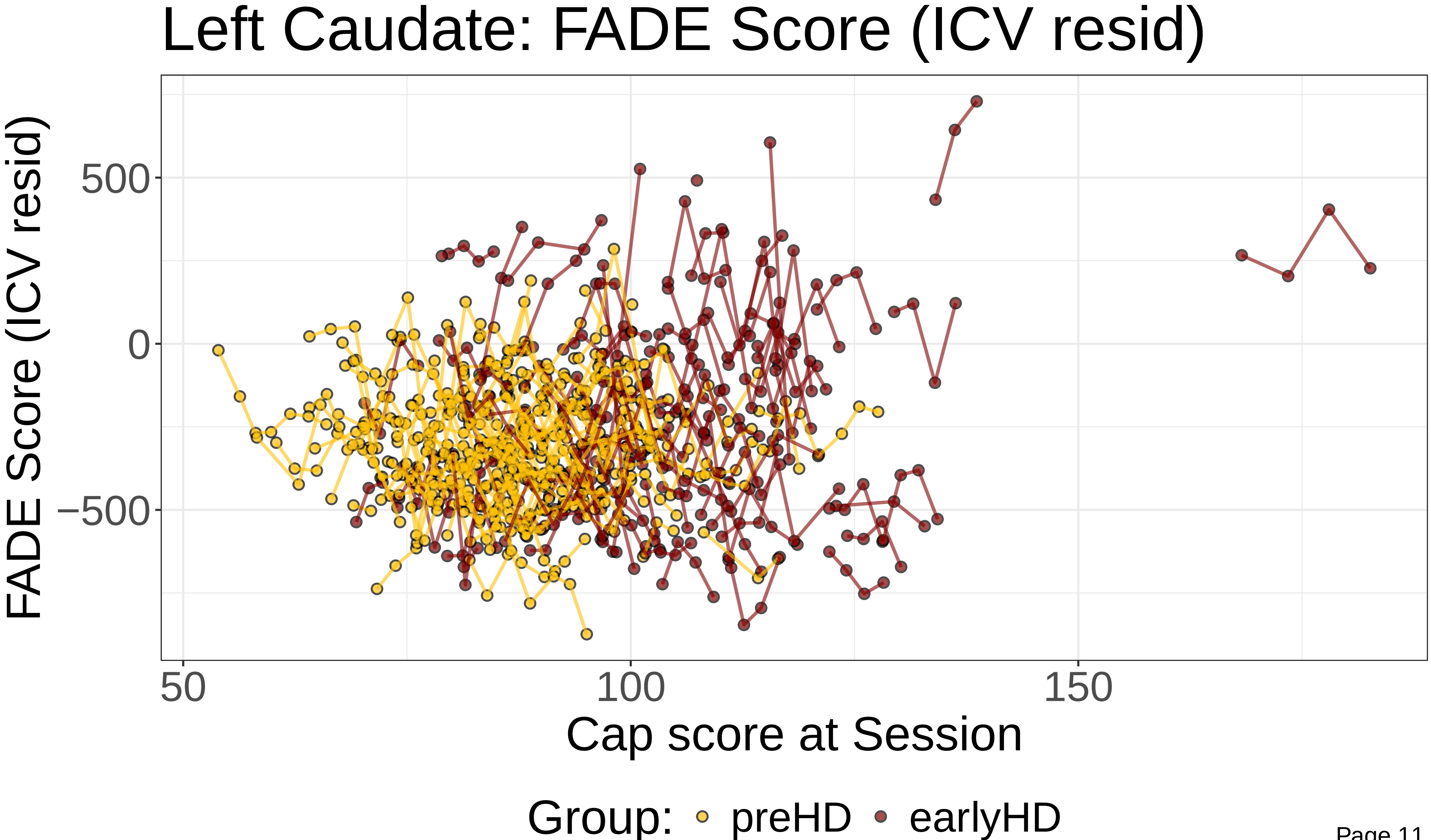

Left Caudate: FADE Score (ICV resid)
FADE Score (ICV resid)
500
0
−500
50
100
150
Cap score at Session
Group:
preHD
earlyHD

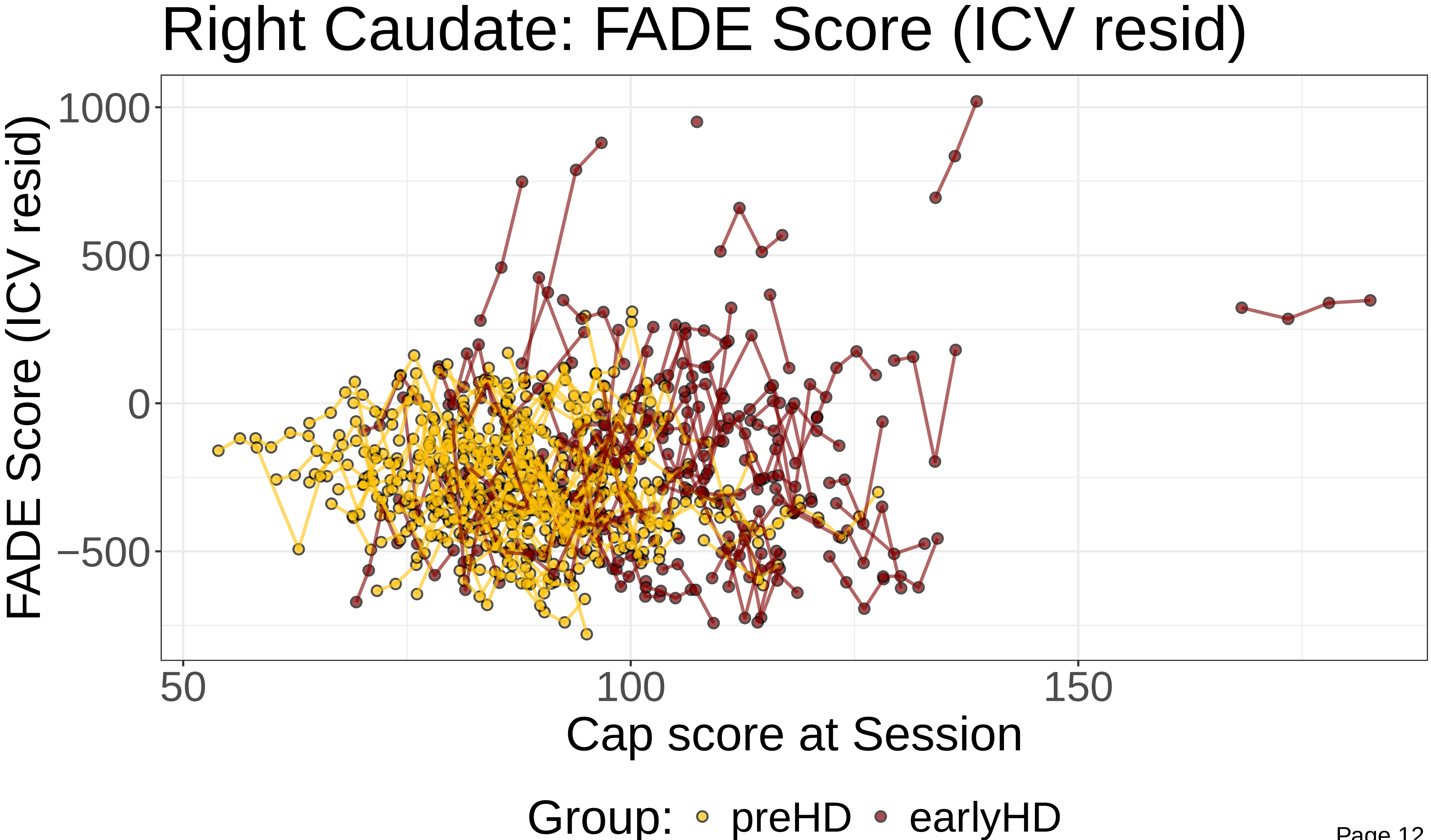

Right Caudate: FADE Score (ICV resid)
FADE Score (ICV resid)
1000
500
0
−500
50
100
150
Cap score at Session
Group: preHD earlyHD

Left Putamen: Volume (ICV resid)

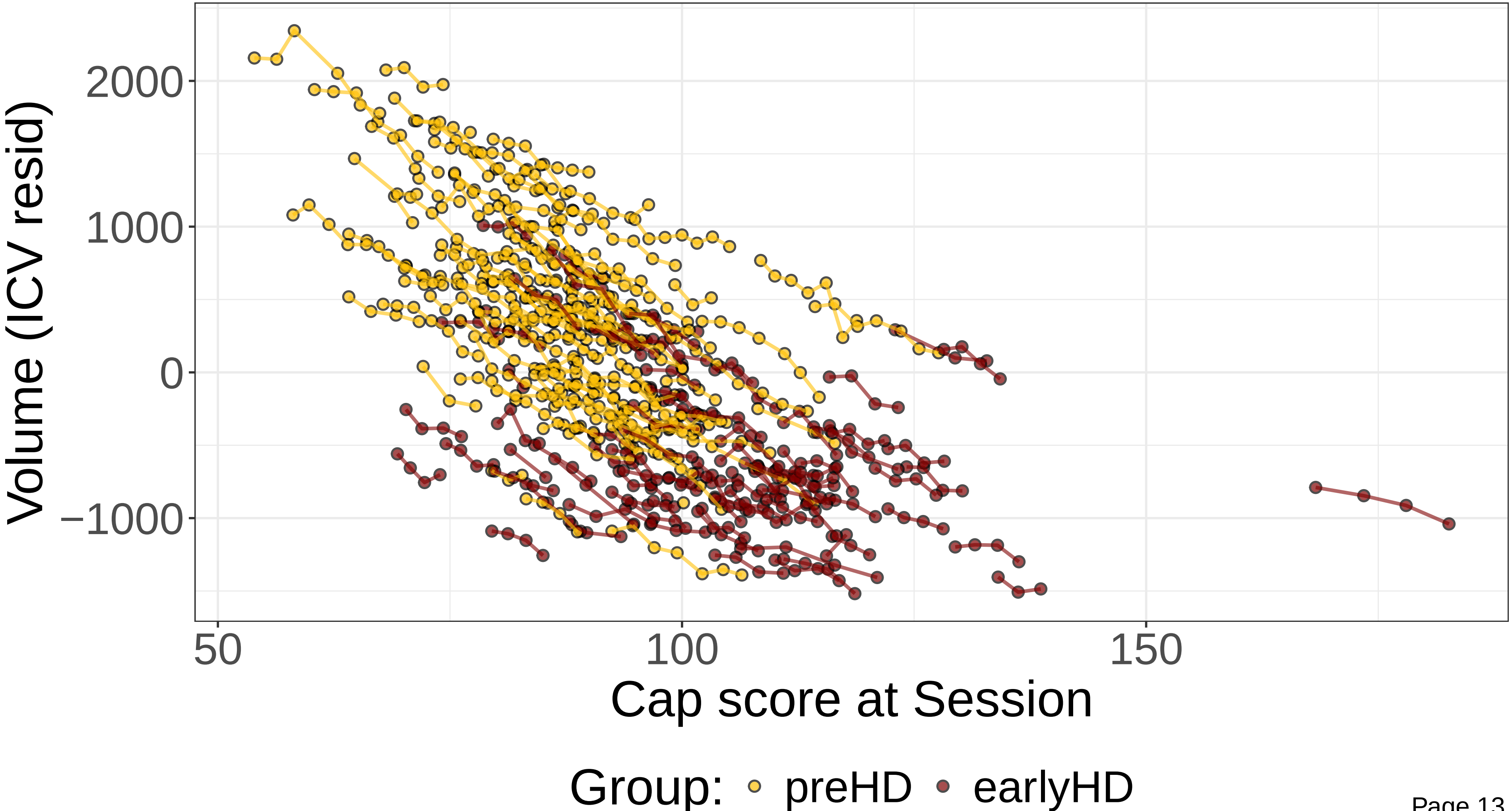

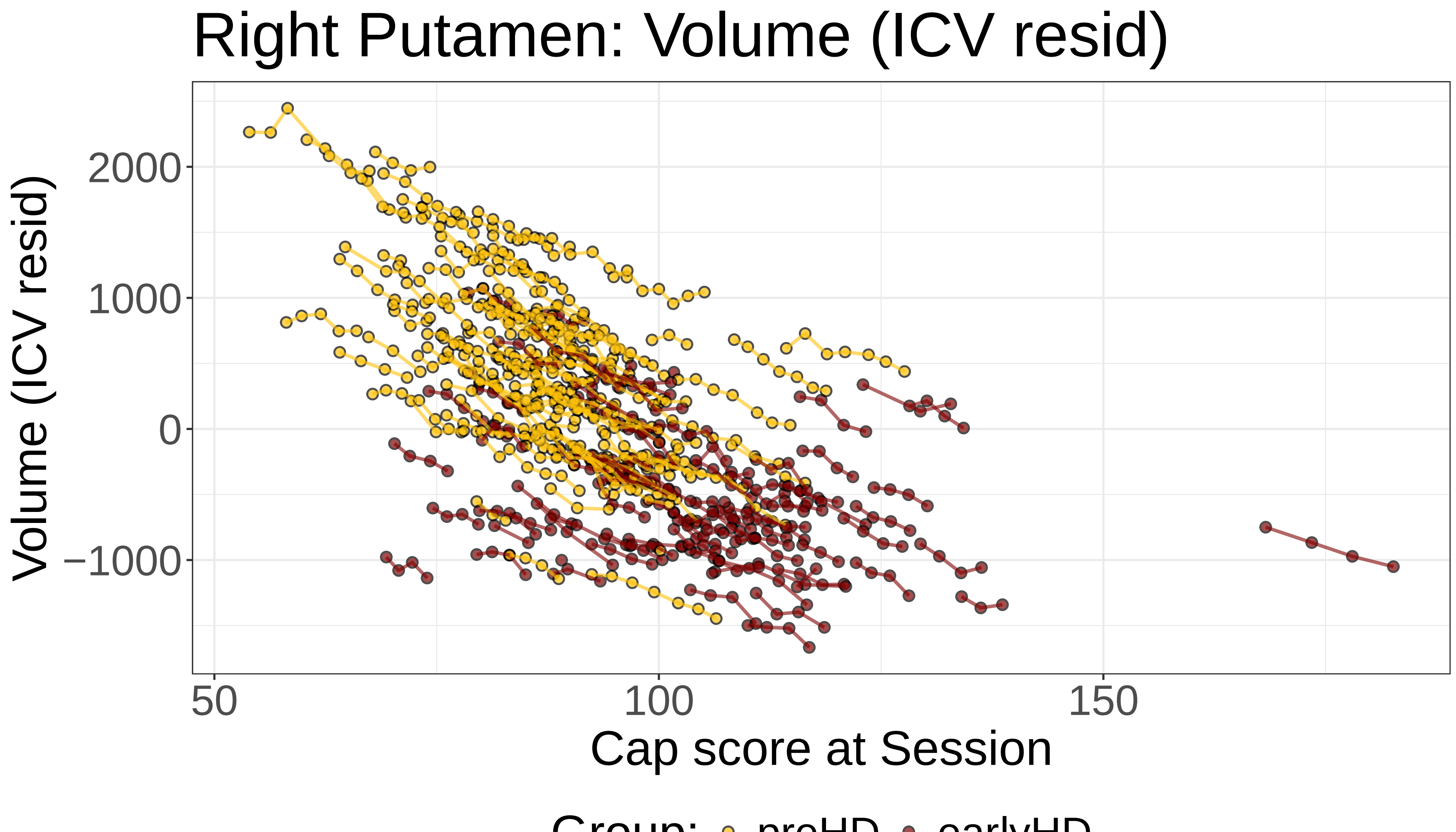

Right Putamen: Volume (ICV resid)
Volume (ICV resid)
2000
1000
0
−1000
50
100
150
Cap score at Session
Group:
preHD
earlyHD

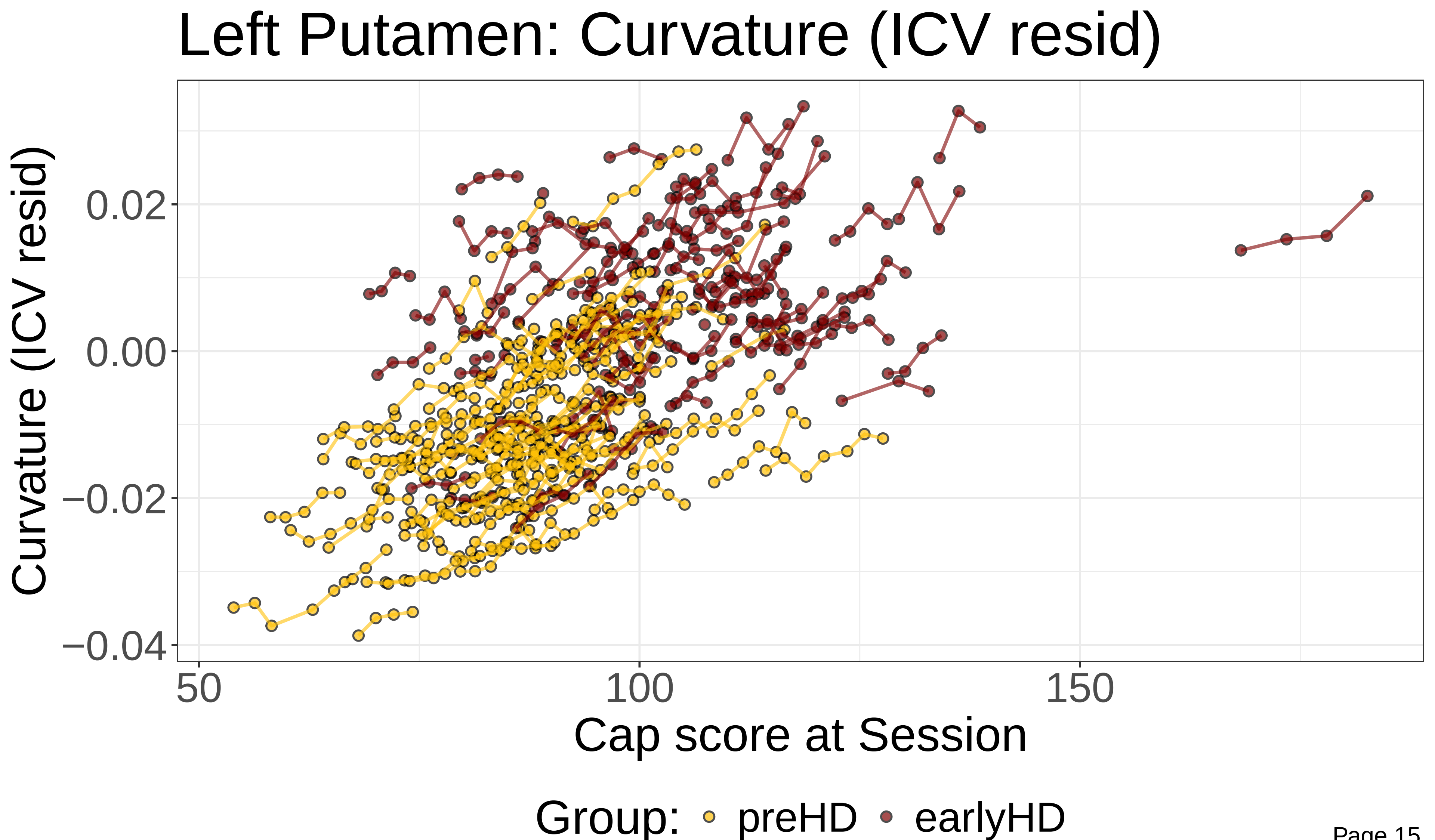
Left Putamen: Curvature (ICV resid)
Curvature (ICV resid)
0.02
0.00
−0.02
−0.04
50
100
150
Cap score at Session
Group:
preHD
earlyHD

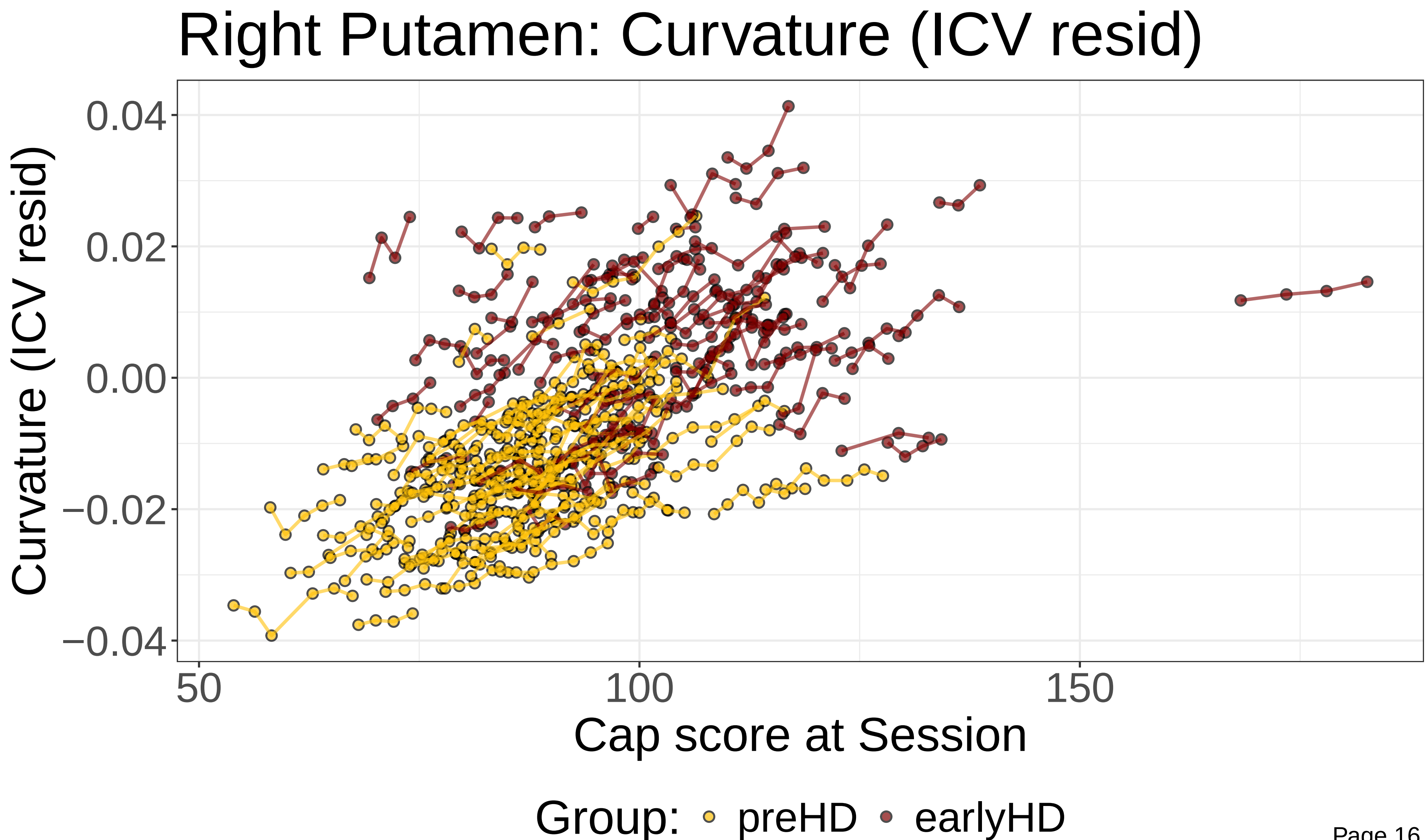
Right Putamen: Curvature (ICV resid)
0.04
0.02
0.00
−0.02
−0.04
Curvature (ICV resid)
50
100
150
Cap score at Session
Group: preHD earlyHD

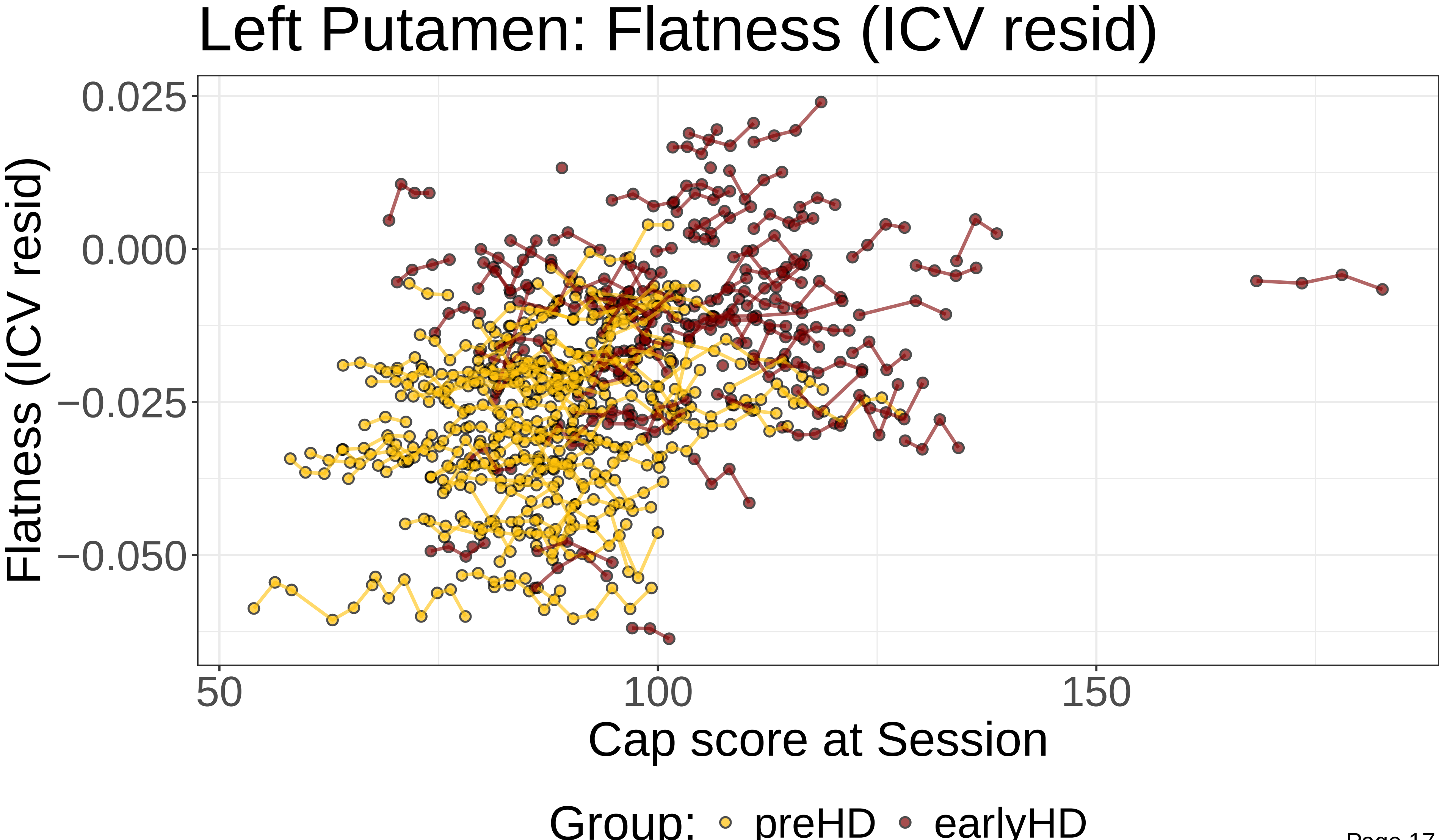
Left Putamen: Flatness (ICV resid)
0.025
0.000
−0.025
−0.050
Flatness (ICV resid)
50
100
150
Cap score at Session
Group: preHD earlyHD

Right Putamen: Flatness (ICV resid)

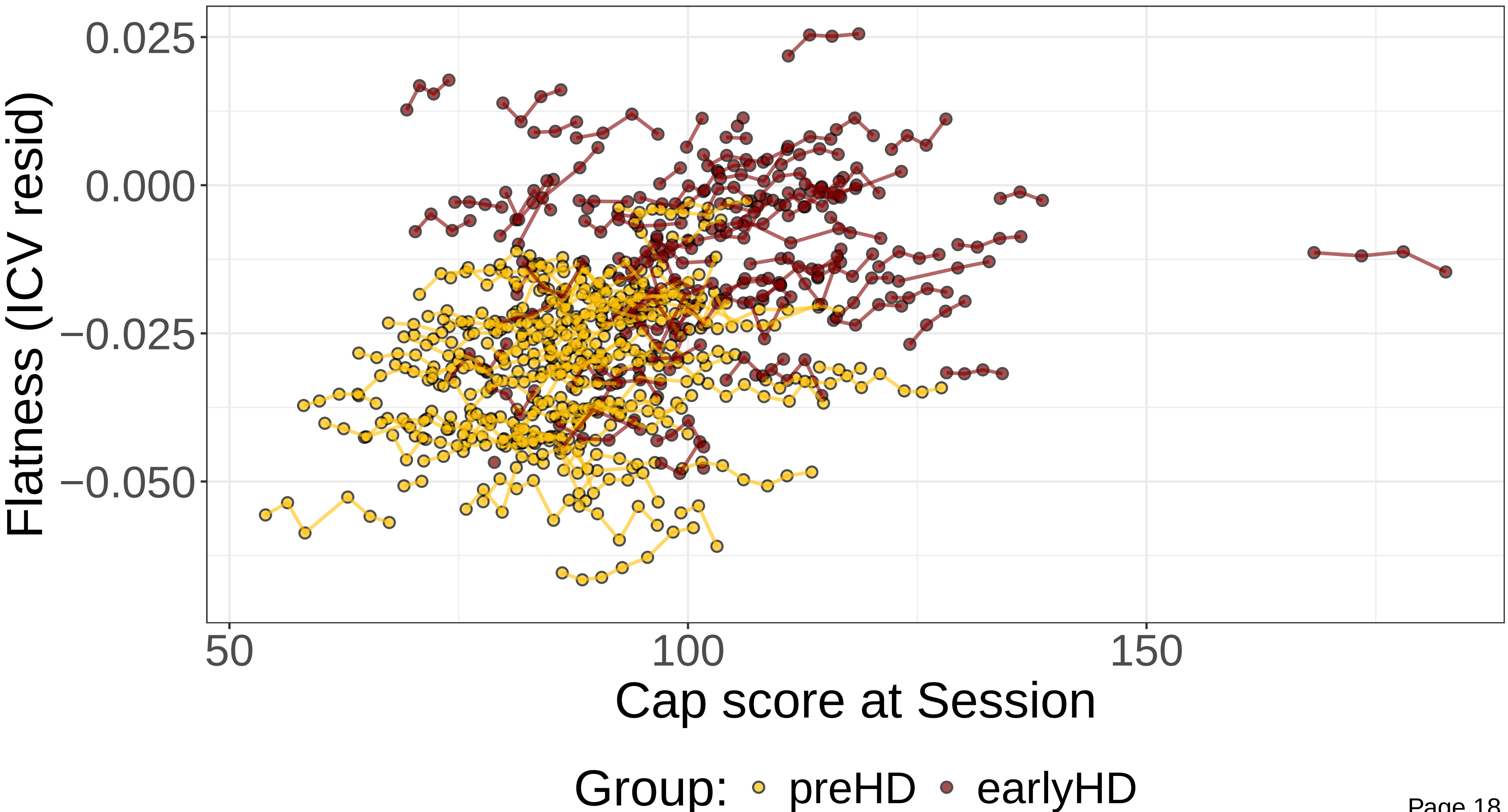

Left Putamen: Elongation (ICV resid)

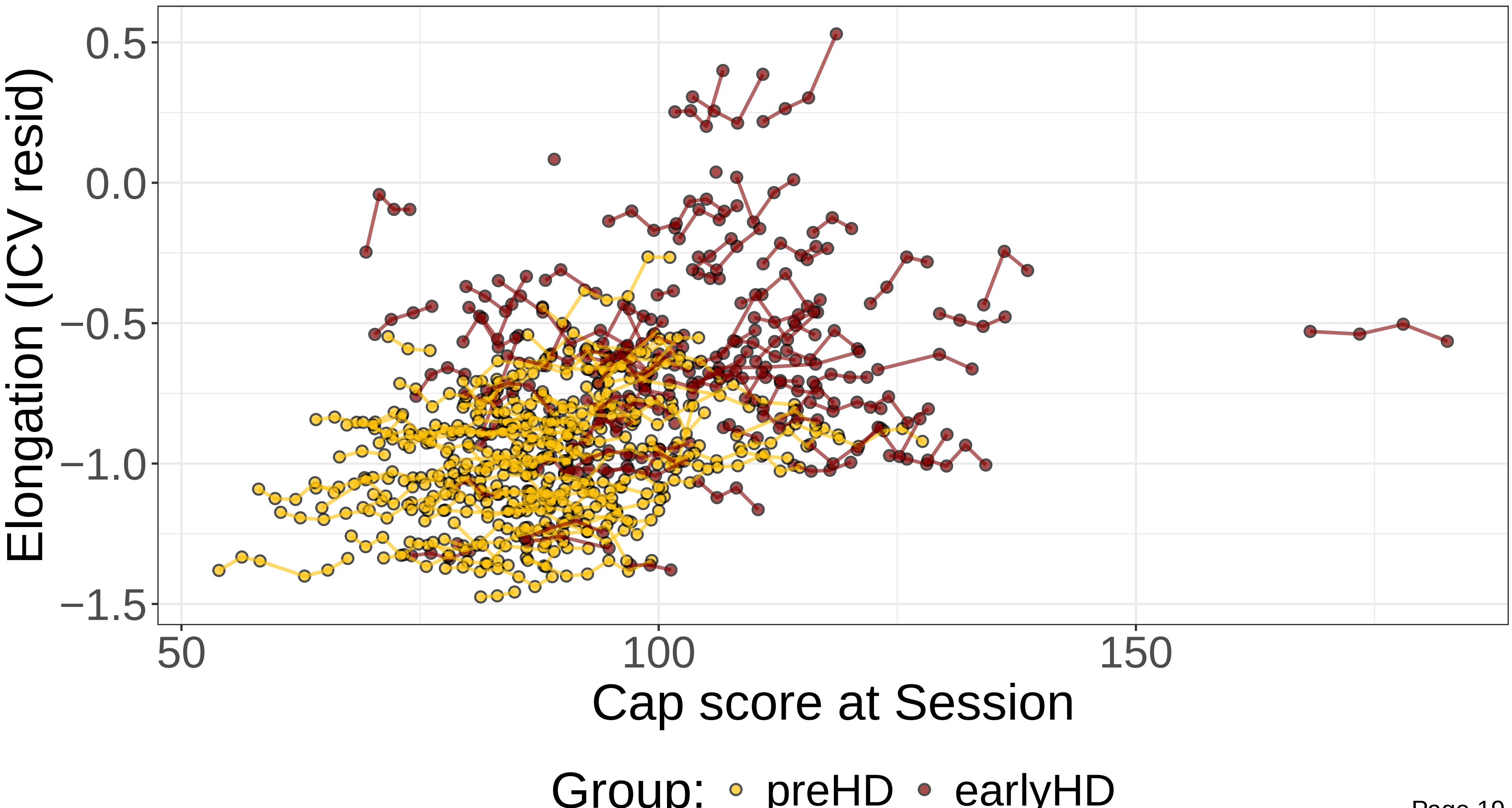

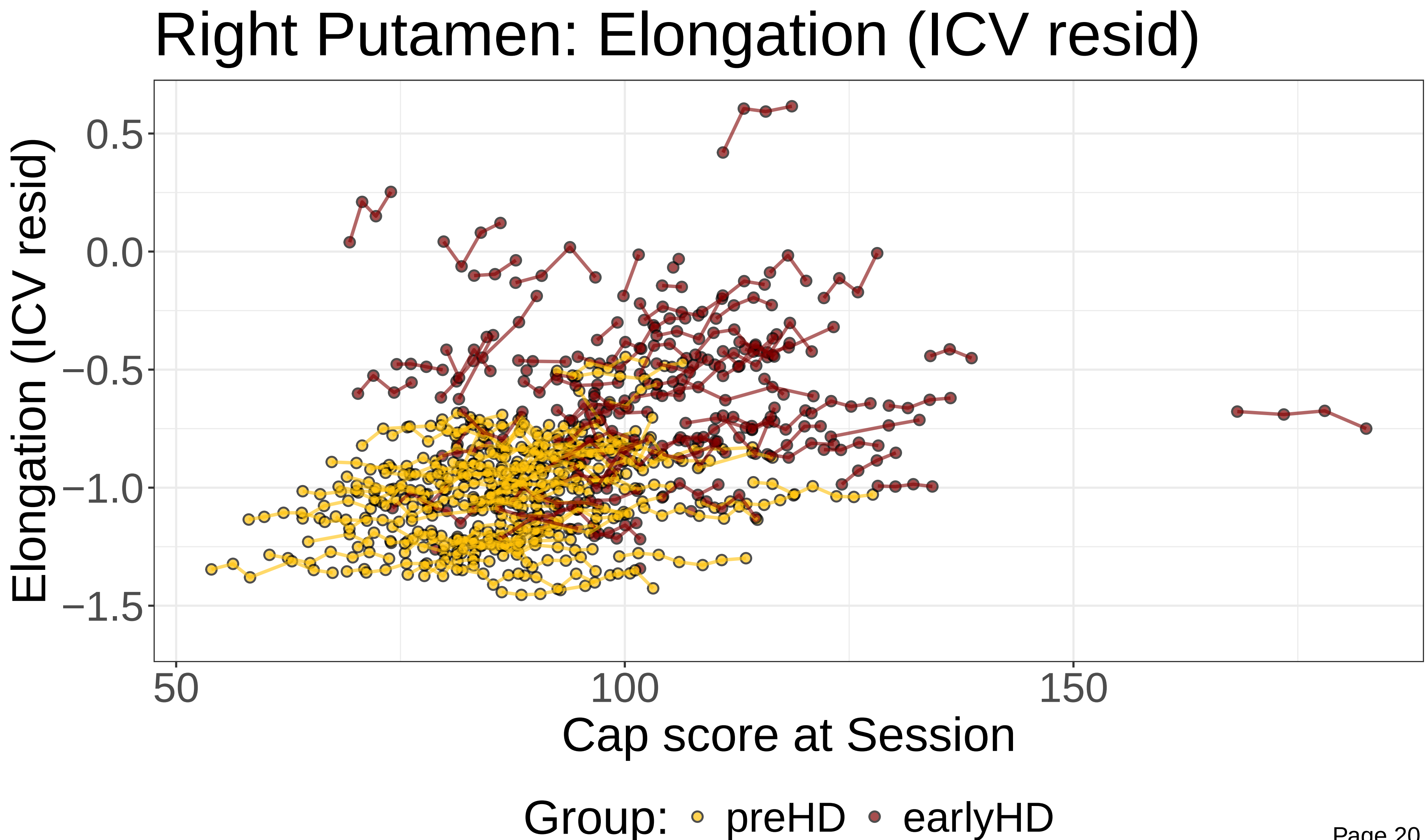

Right Putamen: Elongation (ICV resid)
Elongation (ICV resid)
0.5
0.0
−0.5
−1.0
−1.5
50
100
150
Cap score at Session
Group:
preHD
earlyHD

Left Putamen: Eccentricity (ICV resid)

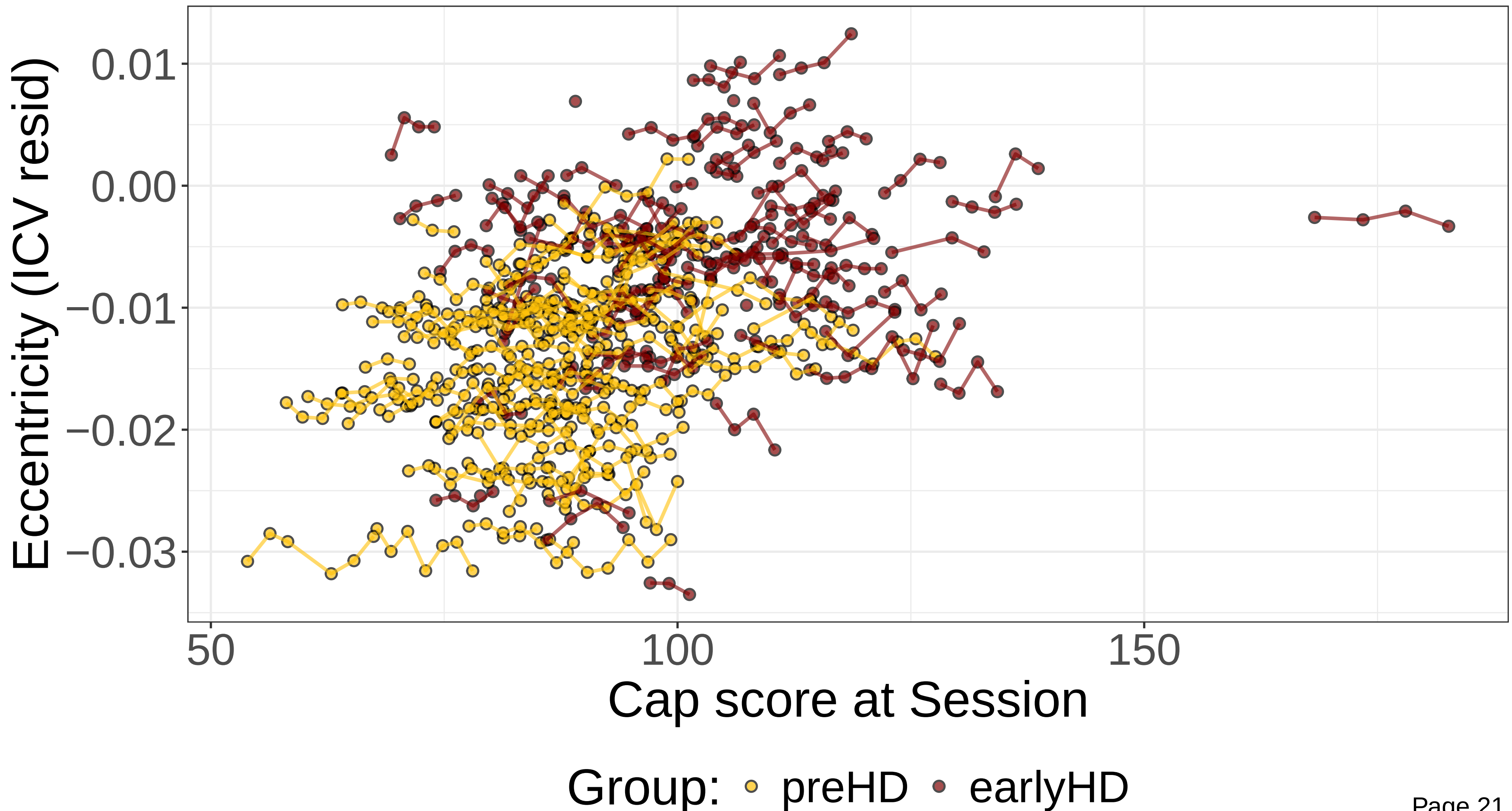

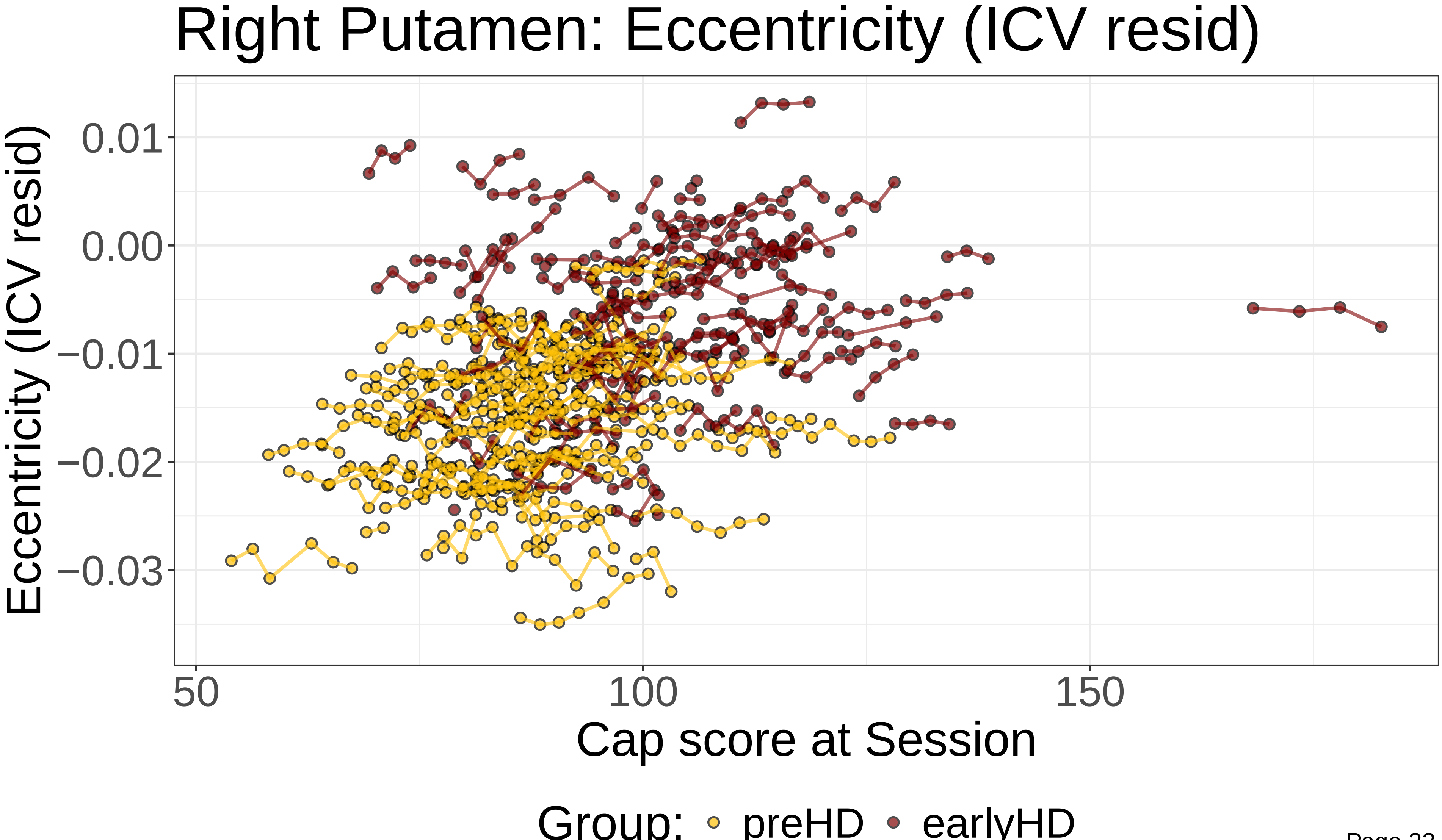
Right Putamen: Eccentricity (ICV resid)
Eccentricity (ICV resid)
0.01
0.00
−0.01
−0.02
−0.03
50
100
150
Cap score at Session
Group: preHD earlyHD

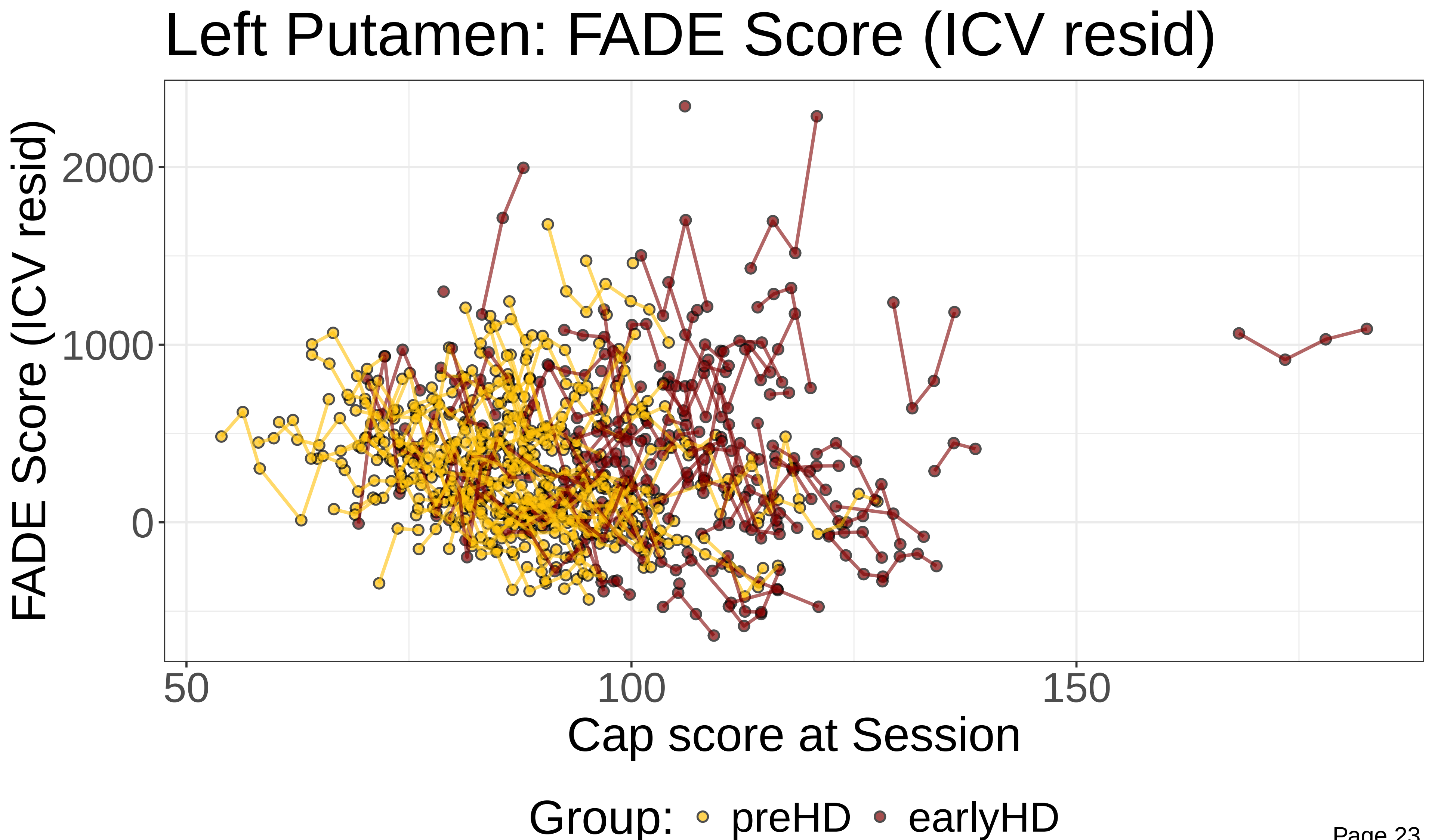
Left Putamen: FADE Score (ICV resid)
FADE Score (ICV resid)
2000
1000
0
50
100
150
Cap score at Session
Group:
preHD
earlyHD

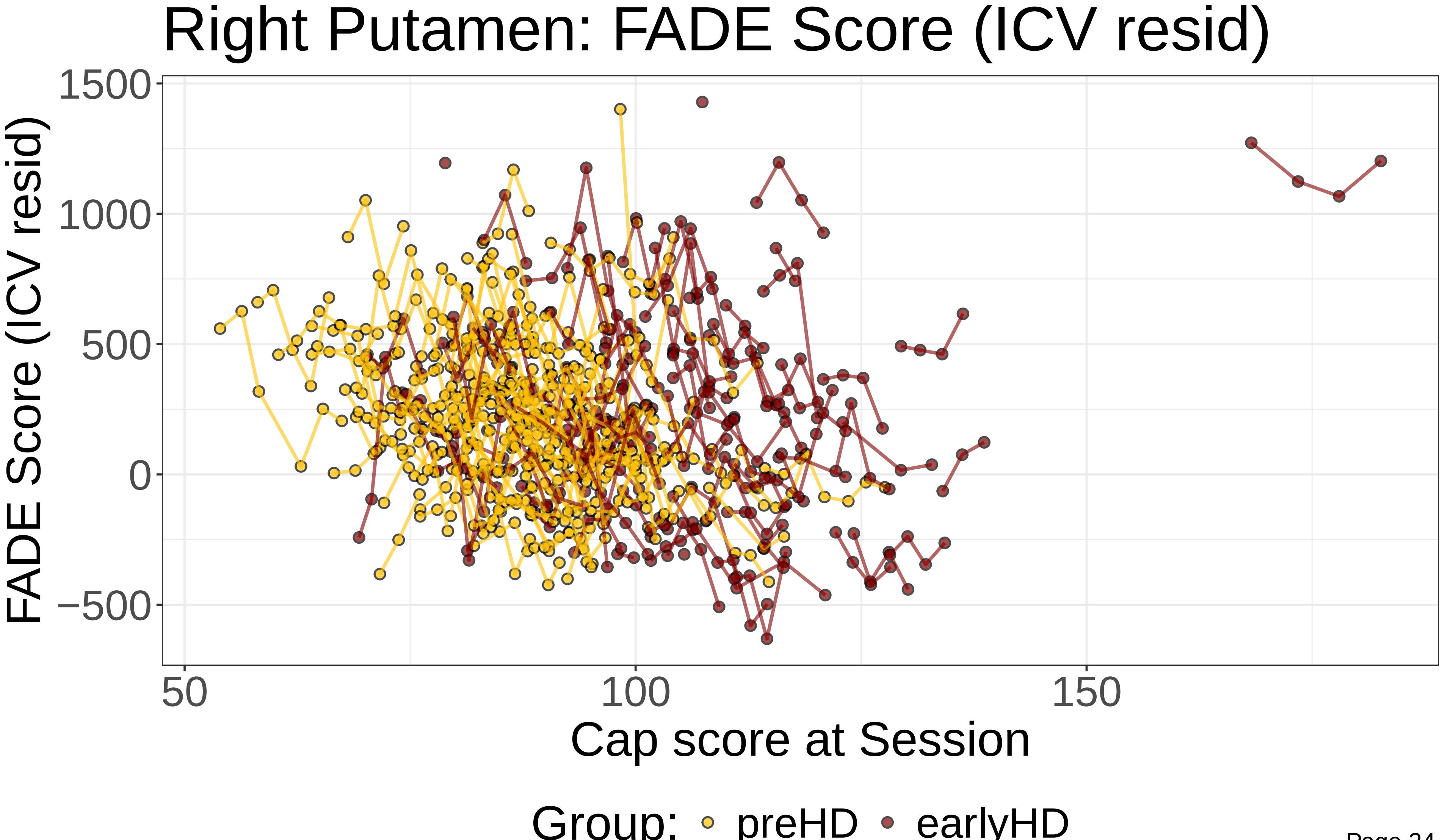

Right Putamen: FADE Score (ICV resid)
FADE Score (ICV resid)
1500
1000
500
0
−500
50
100
150
Cap score at Session
Group:
preHD
earlyHD

Supplementary File - Linear Mixed Models

Table of beta coefficients for the time x group interaction derived from individual linear mixed models for each group comparison and each morphometric measure.

		Longitudinal LMM			
		Caudate		Putamen	
		Left	*Right*	*Left*	*Right*
**HC vs. earlyHD**	Volume	-57.964778	-62.573502	-35.859977	-40.586936
	Curvature	0.000442	0.000566	0.000797	0.001084
	Flatness	0.000797	0.001044	*0.000226*	0.000616
	Elongation	0.124709	0.143937	0.008265	0.016598
	Eccentricity	0.000402	0.000528	*0.000115*	0.000319
	FADE	-17.431165	-17.184835	*10.667268*	*8.421254*
**HC vs. preHD**	Volume	-56.066297	-56.325407	-47.112702	-49.207177
	Curvature	0.0008	0.000915	0.000755	0.000766
	Flatness	0.000837	0.00083	*-0.000068*	*0.000134*
	Elongation	0.068594	0.067736	*-0.000193*	*0.002984*
	Eccentricity	0.000426	0.000422	*-0.000037*	*0.000069*
	FADE	*8.104877*	*4.912391*	*21.293956*	22.76962
**preHD vs. earlyHD**	Volume	*-1.898481*	*-6.248095*	11.252725	*8.620241*
	Curvature	*-0.000358*	-0.000349	*0.000041*	0.000318
	Flatness	*-0.00004*	0.000214	0.000293	0.000482
	Elongation	0.056115	0.076201	0.008458	0.013614
	Eccentricity	*-0.000024*	0.000106	0.000152	0.00025
	FADE	-25.536042	-22.097226	*-10.626688*	*-14.348366*

Non-significant cells after FDR correction (Benjamini-Hochberg, applied within each comparison) are italicized.

Model:

measure ~ years_from_baseline × group_label + (1 + years_from_baseline | subject), fitted separately for each region and hemisphere. All models converged with full random intercept and slope structure.

Notes:

The b coefficient represents how many additional units of change per year the HD group shows relative to the reference group. Positive = HD group increases faster. Negative = HD group

LMM b coefficients are in the original units of each ICV-residualized measure and are not directly comparable across measures due to different scales.
